# Heterogeneity in extracellular vesicle secretion by single human macrophages revealed by super‐resolution microscopy

**DOI:** 10.1002/jev2.12215

**Published:** 2022-04-12

**Authors:** Susanne Dechantsreiter, Ashley R. Ambrose, Jonathan D. Worboys, Joey M. E. Lim, Sylvia Liu, Rajesh Shah, M. Angeles Montero, Anne Marie Quinn, Tracy Hussell, Gillian M. Tannahill, Daniel M. Davis

**Affiliations:** ^1^ The Lydia Becker Institute of Immunology and Inflammation Faculty of Biology, Medicine and Health University of Manchester Manchester UK; ^2^ Department of Cardiothoracic Surgery and Cellular Pathology Wythenshawe Hospital Manchester University NHS Foundation Trust Manchester UK; ^3^ Cellular Pathology Wythenshawe Hospital Manchester University NHS Foundation Trust Manchester UK; ^4^ Department of Anatomic Pathology University Hospital Galway Galway Ireland; ^5^ GSK Medicines Research Centre Stevenage UK

**Keywords:** class II MHC protein, extracellular vesicles, macrophages, single‐cell analysis, super‐resolution microscopy

## Abstract

The diverse origins, nanometre‐scale and invasive isolation procedures associated with extracellular vesicles (EVs) mean they are usually studied in bulk and disconnected from their parental cell. Here, we used super‐resolution microscopy to directly compare EVs secreted by individual human monocyte‐derived macrophages (MDMs). MDMs were differentiated to be M0‐, M1‐ or M2‐like, with all three secreting EVs at similar densities following activation. However, M0‐like cells secreted larger EVs than M1‐ and M2‐like macrophages. Proteomic analysis revealed variations in the contents of differently sized EVs as well as between EVs secreted by different MDM phenotypes. Super resolution microscopy of single‐cell secretions identified that the class II MHC protein, HLA‐DR, was expressed on ∼40% of EVs secreted from M1‐like MDMs, which was double the frequency observed for M0‐like and M2‐like EVs. Strikingly, human macrophages, isolated from the resected lungs of cancer patients, secreted EVs that expressed HLA‐DR at double the frequency and with greater intensity than M1‐like EVs. Quantitative analysis of single‐cell EV profiles from all four macrophage phenotypes revealed distinct secretion types, five of which were consistent across multiple sample cohorts. A sub‐population of M1‐like MDMs secreted EVs similar to lung macrophages, suggesting an expansion or recruitment of cells with a specific EV secretion profile within the lungs of cancer patients. Thus, quantitative analysis of EV heterogeneity can be used for single cell profiling and to reveal novel macrophage biology.

## 1 INTRODUCTION

Macrophages reside within tissues throughout the body where they play a key role in innate immunity and in the coordination of adaptive immunity (Gentek et al., 2014). They have the capacity to respond to numerous environmental stimuli which lead to differing activation states that fulfil specialised functions (Gentek et al., 2014). While macrophage phenotypes are highly heterogeneous, there are two major activated phenotypes; classically activated M1 macrophages and alternatively activated M2 macrophages. In addition, M0 is used to describe pre‐activated macrophages. M1 macrophages are typically pro‐inflammatory whereas M2 display a more regenerative phenotype (Hesketh et al., 2017; Vogel et al., 2014). Alongside M1 and M2 macrophages, environmental cues lead to further specialisation, such as alveolar macrophages in the lung and Kupffer cells in the liver with variable M1 and M2 phenotypes.

Macrophages have multiple roles during immune responses. Detection and phagocytosis of foreign particles opsonized with IgG occurs via ligation of Fcγ receptors (FcγRs) that triggers cellular activation, phagocytosis and the release of inflammatory mediators (Jaumouillé & Grinstein, 2011; Lin et al., 2016; Lopes et al., 2017). Upon encountering opsonized particles, macrophages form a phagocytic synapse before engulfing their target (Goodridge et al., 2011). Beyond directly phagocytosing pathogens, macrophages also interact with cells of the adaptive immune system through the formation of immune synapses, which, similarly to the phagocytic synapse, are structured areas of cellular signalling (Niedergang et al., 2016). Pathogen‐derived antigens can be presented at the macrophage‐T cell interface via MHC class II protein to the T cell receptor (TCR) resulting in T cell activation (Roche & Furuta, 2015). Additionally, macrophages secrete cytokines and extracellular vesicles (EVs) that prime T cells and regulate the immune response in myriad ways (Cianciaruso et al., 2019). Antigen presentation and cytokine release are well‐investigated means by which macrophages modulate the activation and differentiation of the immune system. However, until relatively recently, EVs were understudied and considered a waste removal mechanism rather than essential for intercellular communication and the modulation of recipient cell function (Tetta et al., 2013).

Multiple EV subtypes have been identified, such as exosomes, microvesicles and apoptotic bodies, and these can be characterised by their origin and size (Tetta et al., 2013; Théry et al., 2019). The role of EVs can be both physiological and pathological dependent upon their cargo and membrane composition. Tkach et al. demonstrated that small and large EVs from immature dendritic cells differentially induced CD4^+^ T cell activation (Tkach et al., 2017) and macrophage‐derived EVs have been shown to affect the function of other cells (Lan et al., 2019; Wang et al., 2020). For example, LPS‐induced EV secretion from alveolar macrophages promotes ICAM‐1 expression on epithelial cells (Soni et al., 2016).

Despite much progress, our understanding of the heterogeneity and diverse functions of EV sub‐populations is still in its infancy. Understanding the nuances of EVs has proved challenging because EVs are almost exclusively studied in bulk, which can only reveal the average effects of an entire population (Margolis & Sadovsky, 2019). Therefore, these studies provide limited information with regards to the heterogeneity in EV composition, size and cargo and their cell‐by‐cell variability. One approach that has provided greater insight into individual EVs is super‐resolution microscopy (Colombo et al., 2021; Jiang et al., 2021; Nizamudeen et al., 2018), which has been utilised to identify biomarkers of pancreatic cancer in isolated EVs (Lennon et al., 2019) and to track the intercellular transfer of cancer‐derived EVs (Chen et al., 2016). However, whilst indicating the potential of super‐resolution microscopy in EV research, these studies still disconnect EVs from the individual cells that produce them, making it impossible to understand the contribution and heterogeneity of single cell secretions.

Here, we utilise super‐resolution microscopy to directly observe and characterise the heterogeneity of EVs secreted from individual macrophages and reveal that HLA‐DR is significantly enriched on M1‐like and lung macrophage‐derived EVs. Quantitative single‐cell secretion profile analysis enabled us to classify individual macrophages into five sub‐types according to the EVs they secrete. M0‐ and M2‐like EV secretion profiles were very similar but separated into two types defined by low or very low HLA‐DR expression. The EVs released from individual lung macrophages formed two types that were enriched in HLA‐DR with around 20% of M1‐like macrophages secreting EVs that matched lung macrophages. Other M1‐like cells exhibited a fifth secretion sub‐type. Thus, super‐resolution microscopy can quantitatively categorise EVs on a single cell basis to define macrophage sub‐populations according to their EV secretions.

## 2 METHODS

### 2.1 Primary human macrophage culture

Peripheral blood was acquired from the NHS blood service (Ethics licence: 05/Q0401/108) and density gradient centrifugation was used to isolate peripheral blood mononuclear cells (PBMC). Briefly, peripheral blood was layered over Ficoll–Paque Plus (GE Healthcare) and centrifuged at 1600 rpm for 40 min with no brake. The PBMC layer was carefully aspirated, washed twice in RPMI and red blood cells lysed during a 10 min incubation (Red Blood Cell Lysing Buffer, Sigma Aldrich). Cells were washed and CD14^+^ cells were positively selected using magnetic beads (Miltenyi Biotec) with 1.5 × 10^6^ (Lin et al., 2016) CD14^+^ monocytes seeded into each well of a 6‐well plate. For M0‐like macrophages, monocytes were incubated in X‐Vivo 10 media (Lonza) with 1% human serum (Sigma Aldrich) for 3 days, the media was then exchanged for DMEM (Sigma Aldrich) with 10% Foetal Calf Serum (FCS, Invitrogen), 1% L‐Glutamine, 1% Penicillin, 1% Streptomycin (all Gibco) and 10 mM HEPES Buffered Saline (Sigma Aldrich) for 5 days. M1‐ and M2‐like macrophages were cultured for 8 days in RPMI‐1640 (Sigma Aldrich) supplemented with 10% FCS, 1% L‐Glutamine, 1% Penicillin, 1% Streptomycin and 10 mM HEPES Buffered Saline with 5 ng/ml GM‐CSF (Biolegend) or 50 ng/ml M‐CSF (Peprotech) added for M1‐ and M2‐like macrophages, respectively. Media was replaced every 3 days and cells were used 8–10 days post‐isolation. Before experiments, macrophages were washed with 1 ml of PBS, incubated in non‐enzymatic cell dissociation solution (Sigma Aldrich) for 5 min, collected using a cell scraper (Sarstedt) and resuspended in indicated media. Where indicated, cells were then incubated with 2 μM Cambinol (Sigma Aldrich) for 2 h at 37°C before plating.

### 2.2 Lung macrophage isolation

Human lung tissue was obtained from Manchester Allergy, Respiratory and Thoracic Surgery biobank, collected under National Health Service (NHS) Research Ethics Committee approval (20/NW/0302). Healthy lung tissue from >100 mm proximal to tumour resections was perfused within 24 h of surgery with phosphate‐buffered saline (PBS) using a 0.8‐mm needle equipped syringe (BD Biosciences). The perfusate was layered over Ficoll–Paque Plus and centrifuged (400 × *g*, 35 min), immune cells were collected from the upper surface of the Ficoll–Paque, washed twice with PBS and counted. This process isolated all lung airway immune cells, including macrophages from alveolar spaces and larger airways, these cells were used immediately and macrophages selected via adhesion for experiments. The demographics for all patients that provided tissue for this study are detailed in Supplementary Table S1.

### 2.3 Supported planar lipid bilayers

Bilayers were prepared as previously described (Crites et al., 2015). Briefly, chambered glass coverslips (#1.5 LabTek II; Nunc) were incubated with 1 M HCl in 70% ethanol for 20 min, washed five times with ddH_2_O, incubated with 10 M NaOH for 15 min, washed with ddH_2_O five times and then dried under a stream of argon. Bilayers formed immediately with the addition of 200 μl of liposomes, comprising 99.8% 0.4 mM 1,2‐dioleoyl‐sn‐glycero‐3‐phosphocholine and 0.2% 0.4 mM 1,2‐dioleoyl‐sn‐glycero‐3‐phosphoethanolamine‐N‐cap biotinyl (Avanti Polar Lipids). Without removing the 200 μl of lipids, chambers were washed five times with 500 μl PBS, blocked with 1% Bovine Serum Albumin (BSA; Gibco) for 20 min, incubated with 1 μg/ml streptavidin (Life Technologies) for 20 min, washed five times with PBS, incubated with biotinylated human IgG (Jackson Immuno Research) at indicated concentrations for 20 min and washed five times with PBS. To analyse bilayer mobility, bilayers were stained with goat anti‐human IgG F(ab')2 conjugated to AF488 (Life Technologies) and fluorescence analysed using a Leica TCS SP8 confocal microscope. A 2 × 2 μm region was bleached and recovery assessed over 60 s with frames acquired every second (Supplementary Figure S1). Planar lipid bilayers were immobile to enable the in situ capture of macrophage secretions and to enable the shadow staining for identifying the secretions of individual cells after their detachment.

### 2.4 Flow cytometry

2 × 10^5^ (Lopes et al., 2017) cells were pelleted at 500 × *g* for 3 min, incubated in 48 μl of PBS with 2 μl of the viability dye, Live/Dead Zombie Aqua (Biolegend) for 30 min, washed three times in PBS, blocked in 4% human serum for 15 min, stained with anti‐CD16‐Bv421 (2 μg/ml; 3G8), anti‐CD86‐PE (2 μg/ml; IT2.2), anti‐HLA‐DRα‐AF647 (5 μg/ml; L243), anti‐CD163‐APC‐Cy7 (2 μg/ml; GHI/61; All BioLegend), anti‐CD206‐FITC (2 μg/ml; 19.2; BD), or appropriate isotype controls in 0.5% BSA in PBS for 30 min, washed three times in PBS and fixed in 4% paraformaldehyde for 20 min (all at 4°C). Flow cytometry was performed using a BD FACS Canto II and data analysis carried out using FlowJo v10 (BD Biosciences). An example gating strategy is shown in Supplementary Figure S2.

### 2.5 Enzyme‐linked immunosorbent assay

Supernatants were collected from 5 × 10^4^ (Jaumouillé & Grinstein, 2011) macrophages incubated for 20 h on chambered slides coated with 0.01% poly‐L‐lysine (PLL) and 0–100 μg/ml IgG from human serum (Sigma Aldrich) or bilayers generated using 0–100 μg/ml IgG from human serum, centrifuged at 350 × *g* for 10 min and supernatants retained. The concentration of TNFα and IL‐10 was measured using enzyme‐linked immunosorbent assays (DuoSet, R & D Systems), according to the manufacturer's protocol.

### 2.6 Sample preparation

5 × 10^4^ (Jaumouillé & Grinstein, 2011) macrophages were plated onto bilayers or 0.01% PLL coated chambered glass coverslips with 0 or 10 μg/ml IgG from human serum. Cells were incubated for 20 or 15 min on bilayers or glass slides, respectively, at 37°C. Macrophages were then either detached by incubation with 10 mM lidocaine hydrochloride and 0.5 mM EDTA (both Sigma Aldrich) for 20 min before three washes with PBS using forceful pipetting or fixed in situ with 4% paraformaldehyde in PBS for 20 min. Slides were washed three times with PBS, blocked in 3% BSA with 1% human serum in PBS for 1 h and then stained for 1 h with specified antibodies in blocking solution before three washes with PBS, all at room temperature (RT). Intracellular staining was carried out after permeabilization with 0.1% Triton X‐100 (Sigma Aldrich) for 5 min and blocking with 3% BSA with 1% Human serum for 1 h at RT. Cells were stained with anti‐CD9‐AF488 (10 μg/ml; M‐L13; BD), anti‐CD81‐AF647 or ‐AF488 (10 μg/ml; 5A6), anti‐CD63‐AF488 (10 μg/ml; H5C6), anti‐HLA‐DRα‐AF647 (10 μg/ml; L243; all BioLegend), phalloidin‐AF647 (3 U/mL, Life Technologies) or matched isotype controls (10 μg/ml; MOPC‐21/ MOPC‐173; Biolegend) (Supplementary Figure S3).

Where indicated, key experiments were repeated using blinded samples. Briefly, monocyte‐ or lung‐derived macrophages were detached from tissue culture plates, pelleted by centrifugation at 500 × *g* for 5 min and resuspended in 1 ml of media. These samples were then randomised into coded tubes by colleagues before being used in experiments as previously described. Samples were decoded following data analysis.

### 2.7 Image acquisition

Brightfield images for cell morphology were acquired using a Leica DM IL LED microscope (Leica Biosystems) equipped with a Leica DFC3000 G camera using a 20 × 0.35 NA air objective. Confocal, Stimulated Emission Depletion (STED) and interference reflection microscopy (IRM) were carried out using a Leica TCS SP8 3x STED microscope (Leica Biosystems) equipped with HyD hybrid detectors using a 100 × 1.40 NA oil objective. Sample excitation was achieved using a pulsed white light laser and STED depletion using a 592‐nm continuous wave laser. Stochastic Optical Reconstruction Microscopy (STORM) was performed utilising total internal reflection on a super‐resolution ground state depletion (SR GSD) microscope (Leica Biosystems) using a 160× 1.43 NA oil objective. Samples were incubated with oxygen‐scavenging buffer (560 μg/ml glucose oxidase, 34 μg/ml catalase, 1% β‐mercaptoethanol, 25 mM glucose, and 5% glycerol (all Sigma‐Aldrich), 25 mM HEPES and PBS (pH 8; 0.22‐μm filter sterilised; Thermo Fisher Scientific) and imaged with a 642‐nm or a 488‐nm continuous wave laser for at least 10000 frames (11 ms). A 405‐nm laser was used to reactivate AF647 and AF488 fluorescence.

The location of individual cells following detachment was identified using shadow imaging (Ambrose et al., 2020). Briefly, after activation, wells were washed three times with PBS and then 2 μg/ml of streptavidin‐AF488 (Invitrogen) was added as a pulsed stain for 1 min at RT, wells were washed three times with PBS and cells detached as described above.

### 2.8 Image analysis

Following STORM acquisition, all datasets were background corrected using a custom FIJI (Schindelin et al., 2012) script, as previously described (Ambrose et al., 2020). This removed out‐of‐focus fluorescence and auto‐fluorescence from lung macrophages to improve localisation precision and reconstruction accuracy. Briefly, a grouped z‐projection was created using median values from 200‐frame groupings of each dataset, the grouped z‐projection was then expanded to the number of frames in the original dataset using bicubic interpolation and subtracted from the original dataset.

The corrected dataset was then reconstructed using the ImageJ plugin ThunderSTORM (Ovesný et al., 2014), as previously described (Ambrose et al., 2020; Oszmiana et al., 2016). Datasets were filtered with a B‐spline wavelet filter, approximate localisations calculated using local maximum and sub‐pixel localisations determined using an integrated Gaussian point spread function with maximum likelihood fitting applied over a five‐pixel radius. Localisations that met the following conditions were retained; ‘intensity > 500 and sigma > 50 and sigma < 200 and uncertainty_xy < 30’. Molecules with multiple detections within 20 frames and 40 nm were merged and the dataset was drift corrected by applying cross correlation over three bins with 5× magnification. Finally, the dataset was visualised using average shifted histograms with 10x magnification utilising a final theoretical pixel size of 10 nm.

In dual‐colour datasets, chromatic aberration was corrected by transforming datasets acquired in the 488 nm channel onto those in the 647 nm channel. This transformation was generated by imaging 100 nm TetraSpek Fluorescent Microspheres (Invitrogen) in both channels and calculating a polynomial transformation function using the MultiStackReg plugin in ImageJ. This transformation was applied to every frame of the 488 nm dataset after background correction and before event detection.

Analysis of reconstructed images was carried out using ImageJ with cell size, EV density and EV size analysed using custom ImageJ macros followed by manual assessment to ensure that all EVs were accurately detected (e.g. that larger EVs were not detected as multiple smaller EVs). A lower limit on EV size was set by calculating image resolution using Fourier ring correlation (FRC) utilising the NanoJ Squirrel plugin for ImageJ (Culley et al., 2018). Briefly, representative background‐corrected datasets of EVs imaged with either AF647 or AF488 conjugated CD9, CD63 or CD81 from samples with cells remaining or detached were divided into odd and even frames and images were reconstructed as previously described, divided into square blocks and the FRC calculated for each block. The image resolution was then taken as the mean resolution of all blocks from an individual dataset (Supplementary Figure S4A and B).

EVs imaged with AF647‐conjugated antibodies had a higher resolution (22.5 ± 1.7 nm with cells detached or 34.2 ± 4.7 nm when imaged with the cell remaining) compared to EVs imaged with AF488 conjugated antibodies (44.1 ± 11.7 nm with cells detached). Further to this, localisation uncertainty was calculated using ThunderSTORM with AF647 images having a lower localisation uncertainty (5.8 ± 0.7 nm for EVs imaged without cells and 7.5 ± 1.0 nm when cells remained) compared to images using AF488‐conjugated antibodies (11.55 ± 1.9 nm when cells were detached). Based on this analysis we took the smallest EVs we could accurately detect to be 40 nm, when using AF647‐conjugated antibodies, and 60 nm when using AF488‐conjugated antibodies. HLA‐DR expression was measured using a custom macro that identified EVs using AF488 conjugated tetraspanin antibodies and then analysed HLA‐DR expression and intensity within those EVs in the AF647 channel. HLA‐DR intensity is reported as the mean pixel intensity from individual EVs to ensure that EV size does not influence the value reported for individual EVs. HLA‐DR positive EVs were those with a mean pixel intensity over 5000 AU. Cross channel correlation was calculated using the Fiji plugin Coloc2 (Schindelin et al., 2012).

### 2.9 Image resolution assessed with DNA nano rulers

DNA Nanorulers (GATTAquant DNA technologies), comprising two AF647 fluorophores separated by 30 nm, were used to assess the resolution of our super‐resolution microscopy system and image analysis workflow. Briefly, BSA‐biotin (1 mg/ml) was incubated in chambered glass slides for 5 min and washed three times with PBS followed by a 5 min incubation with neutravidin (1 mg/ml) and three washes with 10 mM MgCl_2_ in PBS. DNA nano rulers were added at between 20 and 200 pM to achieve a final nano ruler density of ∼2 per μm^2^ and incubated for 5 min. Slides were then washed three times with 10 mM MgCl_2_ in PBS and STORM buffer, supplemented with MgCl_2_ to a final concentration of 10 mM, added to slides before imaging. Microscopy and image reconstruction was carried out as previously described, with the exception of images being visualised using 100× magnification to provide a final theoretical pixel size of 1 nm. Representative images are shown in Supplementary Figure S4C and multiple line profiles demonstrate that the mean distance between fluorophore peaks was 31.1 ± 3.4 nm (Supplementary Figure 4D and E).

### 2.10 Isolation of extracellular vesicles

Activating beads were generated by incubating 200 μl of 0.5% (w/v) 6.7 μm streptavidinated polystyrene beads (Spherotech) with 2 ml of 10 μg/ml biotinylated human IgG in PBS for 1 h at RT under continual movement. The beads were washed three times in 5 ml of PBS (pelleted at 3000 × *g* for 10 min), blocked with 3% BSA in PBS for 30 min and pelleted before resuspension in 1 ml of PBS. Up to 5 × 10^6^ (Lin et al., 2016) cells in 1 ml of serum free DMEM supplemented with 2 mM CaCl_2_ were incubated with 20% (v/v) IgG‐coated beads for 90 min with continual mixing.

EV isolation was carried out as previously described (Théry et al., 2006). Briefly, following stimulation, cells were pelleted by centrifugation at 350 × *g* for 15 min, then the retained supernatant was centrifuged at 2000 × *g* for 15 min to remove cell debris. These supernatants were then used for EV capture experiments or were further centrifuged at 10,000 × *g* for 40 min to pellet ‘large EV’, with ‘small EV’ pelleted by centrifuging the resulting supernatant at 100,000 × *g* for 90 min. EV pellets were washed once in PBS and re‐pelleted by repeating the respective centrifugation steps before re‐suspension in serum free media and plating on 0.01% coated PLL slides for microscopy.

### 2.11 EV capture

Cell and debris‐free supernatants from bead‐activated macrophages were incubated on chambered glass coverslips coated with 1% BSA, 0.01% PLL, protein‐rich planar lipid bilayers generated with 10 μg/ml IgG from human serum or 0.01% PLL with anti‐CD9, anti‐CD63 and anti‐CD81 mAbs (5 μg/ml of each; clones M‐L13, H5C6 and 5a6, respectively) for 1 h at RT. Slides were washed three times with PBS, blocked with 3% BSA with 1% human serum for 1 h at RT before staining with anti‐CD9‐AF647, anti‐CD63‐AF647 and anti‐CD81‐AF647 mAbs (10 μg/ml of each; clones M‐L13, H5C6 and 5a6, respectively) for 1 h at RT and imaged as previously described.

### 2.12 Mass spectrometry sample preparation

EV pellets were re‐suspended in 25 μl lysis buffer (100 mM Glycine, 5% SDS and 8 M Urea at pH 7.55) and then centrifuged at 13,000 × *g* for 8 min. The supernatant was then reduced with 100 mM dithiothreitol and alkylated with 300 mM iodoacetamide, centrifuged at 14,000 × *g* for 10 min and acidified to a final concentration of 1.2% phosphoric acid. For every 50 μl of acidified protein, 300 μl of S‐Trap binding buffer (90% methanol with 100 mM triethylammonium bicarbonate (TEAB), pH 7.1) was added and the solution was loaded onto S‐Trap columns (ProtiFi) by centrifugation at 4000 × *g* for 2 min. Columns were washed four times with 150 μl S‐Trap binding buffer and centrifuged at 4000 × *g* for 2 min, samples were digested in 20 μl 50 mM TEAB and 1 μg trypsin (Sequencing grade modified Trypsin, Promega) for 1 h at 47°C. Peptides were eluted using 65 μl 50 mM TEAB, washed with 65 μl 0.1% formic acid, then washed with 30 μl of 30% acetonitrile with 0.1% formic acid followed by centrifugation at 4000 × *g* for 2 min after each wash. Samples were desalted using FiltrEX desalt filter plates (Corning) with 10 μl of POROS R3 beads (Thermo Fisher Scientific) per sample and then dried using a Heto vacuum centrifuge for 90 min at RT.

Liquid chromatography with tandem mass spectrometry was performed using an UltiMate® 3000 Rapid Separation LC (Dionex Corporation) coupled to a QE HF (Thermo Fisher Scientific) mass spectrometer. Three micro litre of sample was loaded onto a C18 analytical column (250 μm x 75 mm) (Waters) at a flow rate of 300 nl/min for 13 min, after which the flow was reduced to 200 nl/min over 30 s with the column gradient changed linearly from 5 to 18% acetonitrile in 0.1% formic acid over 34.5 min, followed by 18–27% over 8 min and then 27–60% over 1 min with the flow rate increased to 300 nL/min for the final 5 min of the run. Data was acquired in positive mode with data‐dependent analysis selecting for the top‐12 intensity peptides between a range of 300 and 1750 m/z, with a charge state of 2–4 and dynamic exclusion set at 15 s.

Raw data files were produced using XCalibur and peptides were identified with SEQUEST HT and quantified from mass spectra using Proteome Discoverer v2.4 (Thermo Scientific) based on the UniProtKB/Swiss–Prot human database (SwissProt TaxID = 9606; v2017‐10–25 containing 556,006 sequences). Search parameters were set to a maximum of two missed cleavages, for peptides between 6 and 144 amino acids in length with mass tolerances set to 10 ppm for precursors and 0.02 Da for fragment ions. Static modifications were restricted to cysteine carbamidomethylation (+57.021 Da) and dynamic modifications to oxidation of methionine (+15.995 Da), and N‐terminal acetylation (+42.011 Da). Percolator was used to filter spectral identifications to <1% FDR (Käll et al., 2007). Quantification was performed in a label‐free manner, utilising Proteome Discoverer's precursor ion quantifier. Precursor ion features were chromatographically aligned with an error of 3 min, and mass tolerance of 10 ppm. Both unique and razor peptides were used for quantification based on precursor area with all peptides being used for normalisation by total peptide amount. Protein roll‐up and pairwise ratio calculations excluded peptides with dynamic modifications. Protein abundance was calculated through summed abundances for the top N (3). Maximal pairwise ratios were set to 100. Pathway analysis was carried out as an ordered query, weighted on raw protein abundance per sample, using G:Profiler (Müller et al., 2019) and heatmaps were created in R (R Core Team 2020) (version 4.1.0).

### 2.13 Statistical analysis

Data is displayed as mean ± standard deviation, unless otherwise stated, with the number of distinct donors indicated. Datasets were evaluated for distribution normality using a Shapiro–Wilk test and normally distributed data was compared with either a *t*‐test or one way ANOVA, while non‐normally distributed data was compared with either a Mann–Whitney test or Kruskal–Wallis test, as appropriate. A Gaussian fit was applied to histograms to determine modal values with all analysis carried out using GraphPad Prism v8 (GraphPad Prism Software). A power analysis was carried out to determine the number of single cell secretion profiles needed in the second blinded cohort to confidently replicate the findings of the initial cohort. Due to the similarity of the analysis to single cell sequencing, a single cell sequencing sample size calculator was utilised, SCOPIT (Single‐Cell One‐sided Probability Interactive Tool) (Davis et al., 2019). A requirement for 209 cells was calculated based on a probability of 0.9 to identify the rarest population comprising 25 cells at a frequency of 0.1488.

## 3 RESULTS

### 3.1 Different macrophage phenotypes respond to opsonised surfaces

Macrophages are highly heterogeneous, with a variety of specialised roles. To reflect macrophage diversity, we differentiated human monocytes to yield ‘M0‐like’, ‘M1‐like’ (pro‐inflammatory) and ‘M2‐like’ (regenerative) macrophages (Supplementary Figure S5A). When adhered to a flat surface, differentiated M0‐ and M1‐like macrophages appeared rounded, while M2‐like macrophages were elongated (Supplementary Figure S5B), corresponding with their known morphological characteristics (Rostam et al., 2017; Vogel et al., 2014). The expression of phenotypic surface markers on differentiated macrophages was assessed by flow cytometry (Supplementary Figure S5C). All macrophages, regardless of differentiation, expressed both CD86 and MHC class II, in accordance with prior observations (Hesketh et al., 2017; Wang et al., 2020). A small proportion of M0‐, M1‐ and M2‐like macrophages expressed CD16; 17.5 ± 6.1%, 22.9 ± 6.1% and 20.7 ± 1.2%, respectively. CD16 is expressed on a subset of monocytes and a smaller proportion of macrophages with variable M1 or M2 association (Hesketh et al., 2017). CD163 is a well‐established marker of M2 macrophages (Vogel et al., 2014) and CD206 has been identified predominantly on M2‐like macrophages, although some monocyte‐derived macrophage differentiation regimes have also led to reports of its expression on M1‐like macrophages (Bertani et al., 2017). Here we observed CD163 expressed exclusively on M2‐like macrophages while CD206 was highly expressed on both M1‐ and M2‐like macrophages (Supplementary Figure S5C).

**FIGURE 1:**
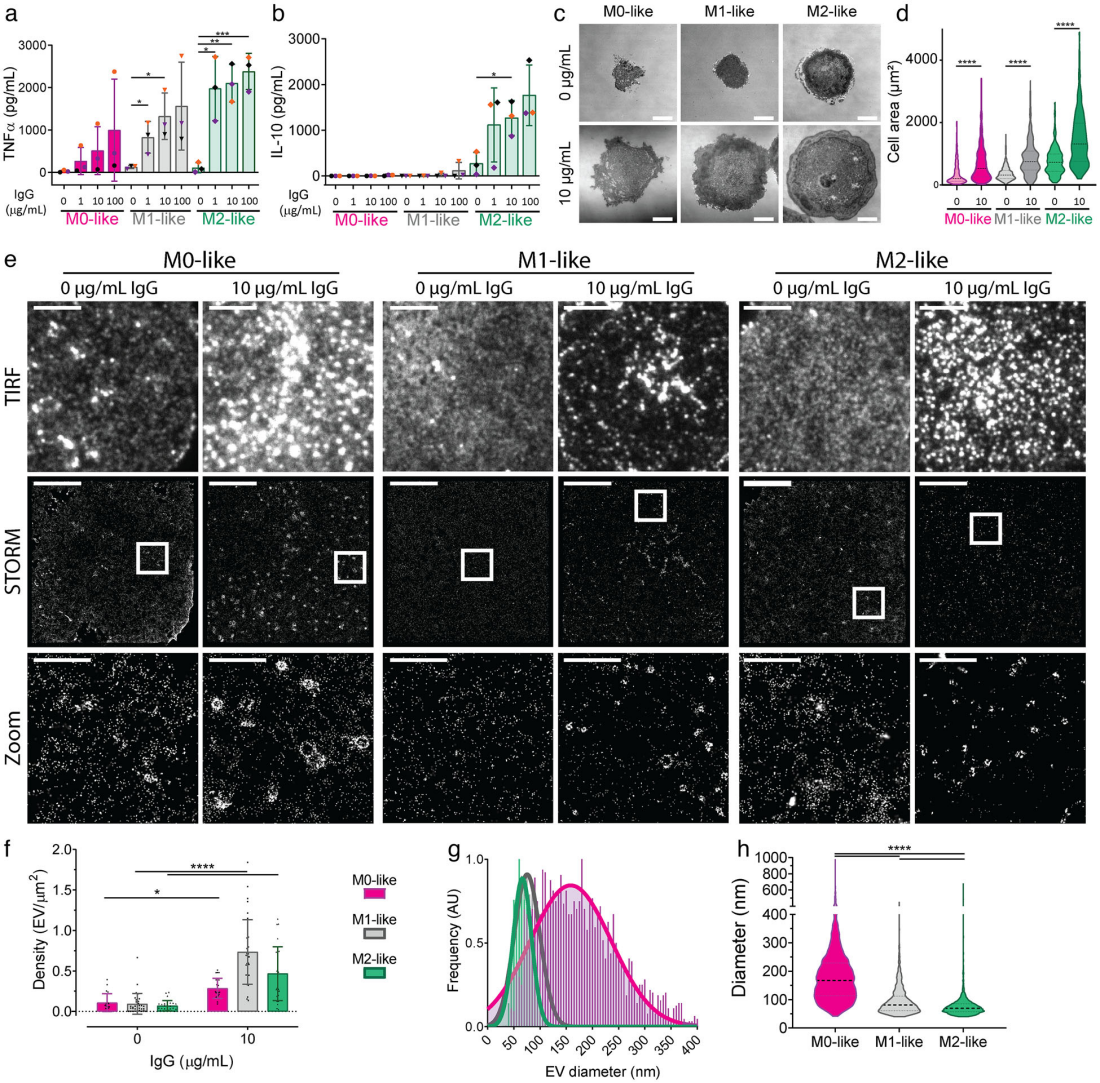
CD81 is enriched in EVs secreted at the surface membrane of activated macrophages. (a,b) Macrophages were incubated on glass slides coated with PLL and indicated concentrations of IgG from human serum for 20 h and supernatants were analysed for TNFα (a) and IL‐10 (b) by ELISA. *n* = 3 individual donors and experiments, mean ± SD. (c,d) Macrophages were incubated for 15 min on glass slides coated with 0.01% PLL only (0 μg/ml IgG) or 0.01% PLL and IgG (10 μg/ml IgG) and fixed. (c) Representative interference reflection microscopy (IRM) images (Scale bars; 10 μm). (d) Cell area distributions. Representative of three individual donors and experiments. (e–h) Macrophages were incubated on glass slides coated with 0.01% PLL or 0.01% PLL + 10 μg/ml IgG from human serum for 15 min, fixed, blocked and stained with anti‐CD81‐AF647 mAb, then phagocytic synapses were imaged using STORM. (e) Representative images, with and without activation are shown for M0‐, M1‐ and M2‐like macrophages (Scale bar; 10 μm, White box; STORM Zoom). (f) Density of EVs secreted from macrophages. (g) Relative frequency of EV diameter (10 nm binning). (h) Distribution of EV diameters. *n* = 3 individual donors and experiments. *, *p* ≤ 0.05; **, *p* ≤ 0.01; ***, *p* ≤ 0.001; ****, *p* ≤ 0.0001. Statistical significance assessed by Mann‐Whitney test (A,B) or Kruskal‐Wallis test (D, F–H)

Next, the functional response to Fc receptor ligation on IgG‐containing lipid bilayers or opsonised glass slides was assessed by measuring release of the multifunctional cytokine TNFα, which is associated with M1, and the M2 macrophage subtypes M2b and M2d, or IL‐10 release, an M2‐associated cytokine (Wang et al., 2019). We observed TNFα secretion, from M0‐, M1‐ and M2‐like macrophages while IL‐10 was only secreted from M2‐like macrophages (Figure 1a and b and Supplementary Figure S5D), both in a concentration‐dependent manner. The ability of differentiated macrophages to respond to IgG was further confirmed using IRM, with all macrophages spreading and forming larger contacts with activating surfaces, indicative of a frustrated phagocytic synapse (Figure 1c and d). These data demonstrate that human monocytes were differentiated to produce distinct macrophage phenotypes (M0, M1 and M2) that activate in a dose‐dependent manner upon stimulation via Fc receptors.

### 3.2 Activated macrophages secrete CD81 enriched vesicles following activation

EVs are an important form of intercellular communication at cellular synapses that are identifiable by their transmembrane enrichment of tetraspanins (Choudhuri et al., 2014; Saliba et al., 2019; Tetta et al., 2013). We sought to investigate whether EVs were secreted upon Fc receptor ligation using IgG‐coated glass slides. This created a flattened model of the phagocytic synapse that was optimal for TIRF imaging. Macrophages were activated for 15 min on IgG‐coated glass slides and stained for the tetraspanin CD81. Without activation, CD81 was homogenously distributed throughout the membrane (Figure 1e, 0 μg/ml IgG). However, upon activation, bright spots of CD81 were observed at macrophage synapses using TIRF microscopy (Figure 1e, 10 μg/ml IgG). The super‐resolution technique STORM was used to resolve these bright spots, which revealed CD81 to be configured in nanoscale rings (Supplementary Figure S6), as has previously been observed when analysing EVs (Han et al., 2021; Jiang et al., 2021).

The density of EVs increased upon activation, with M1‐like macrophages displaying the highest (0.74 ± 0.40 EV/μm^2^) and M0‐like the lowest density (0.29 ± 0.12 EV/μm^2^) (Figure 1f). Following stimulation, EV density continued to increase for 120 min for all three macrophage subtypes. However, some level of EV secretion was evident for M0‐like macrophages even without IgG activation during prolonged incubations (Supplementary Figure S7). The EVs at the membrane of activated M0‐like macrophages were largest with a modal diameter of 159 ± 78 nm and a mean diameter of 182 ± 95 nm, whilst M1‐like and M2‐like EV were smaller with modal diameters of 75 ± 24 nm and 66 ± 18 nm and mean diameters of 94 ± 47 nm and 77 ± 41 nm, respectively (Figure 1g and h).

**FIGURE 2:**
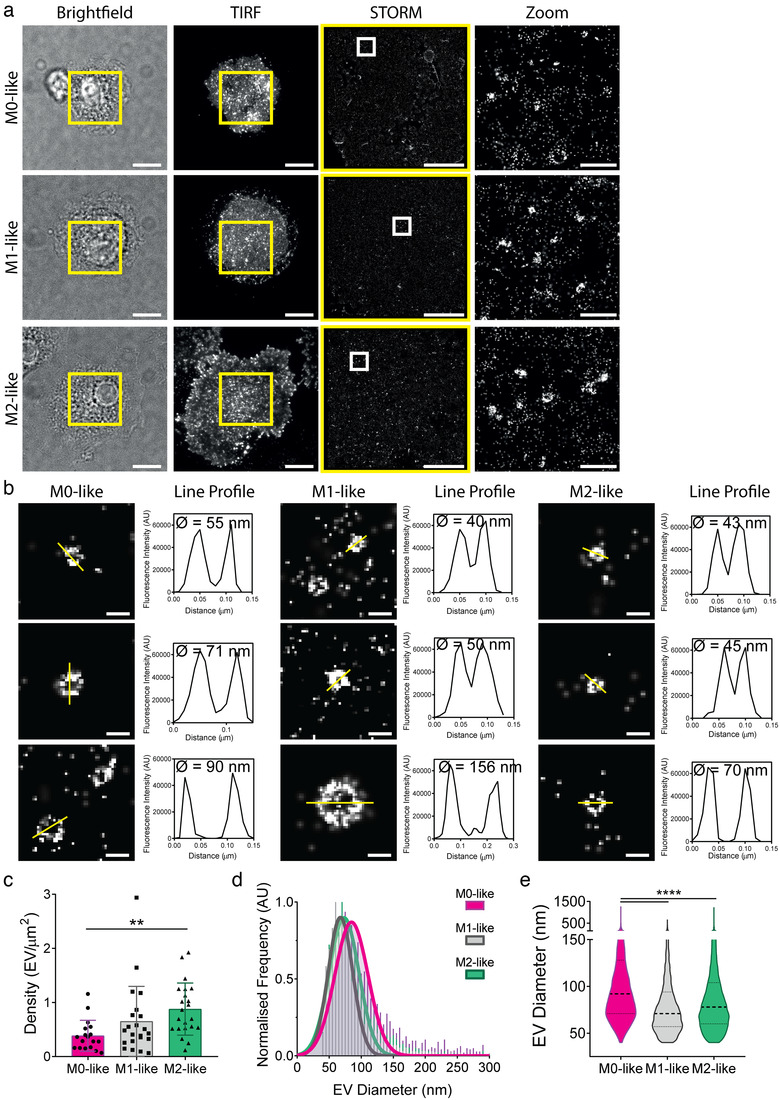
Macrophages activated on lipid bilayers secrete CD81‐enriched EV. M0‐, M1‐ and M2‐like macrophages were plated on planar lipid bilayers containing 10 μg/ml IgG for 20 min, fixed, blocked and stained with anti‐CD81‐AF647 mAb before imaging by STORM. (a) Representative brightfield, TIRF, STORM and Zoom images (Scale bar; 5 μm, Zoom; 1 μm, Yellow box; TIRF crop for STORM, White box; STORM Zoom). (b) Representative zoom images of EVs with line profiles. Scale bar; 100 nm. (c–e) Quantitative analysis of EVs including density, defined as the number of EVs per μm^2^ (c), a histogram of detected diameters normalised to the mode of each histogram (d) and violin plots of the detected EV diameters (e). *n* = 3 individual donors and experiments. **, *p* ≤ 0.01; ****, *p* ≤ 0.0001; Statistical significance assessed by Kruskal‐Wallis test

To mimic cell membrane interactions in the absence of the potential activating effects of PLL (Santos et al., 2018), we generated planar lipid bilayers containing human IgG and incubated M0‐, M1‐ and M2‐like macrophages on them, for 20 min, to stimulate EV release. We again observed bright spots of CD81 in TIRF images that corresponded to nanoscale rings of CD81 when resolved with STORM (Figure 2a). Line profiles verified that CD81 has a ring‐like structure (Figure 2b). EV density was again lowest at the membrane of M0‐like macrophages, 0.39 ± 0.29 EV/μm^2^, while M1‐ and M2‐like macrophages had higher mean densities of 0.65 ± 0.65 and 0.88 ± 0.48 EV/ μm^2^, respectively (Figure 2c). M2‐like EV secretion was increased when activated on planar lipid bilayers compared to glass (Figure 1f). In addition, EVs from M0‐like macrophages had a smaller modal and mean diameter (84 ± 26 nm and 115 ± 75 nm, respectively), when stimulated by ligand‐rich bilayers compared to 159 ± 78 nm (modal) and 182 ± 95 nm (mean) on glass (Figure 2d and e). M1‐ and M2‐like macrophages secreted EVs with similar diameters on both surfaces. M1‐like macrophages secreted EVs with a modal diameter of 68 ± 19 nm and mean diameter of 87 ± 56 nm while M2‐like EVs had a modal diameter of 73 ± 23 nm and mean diameter of 94 ± 64 nm following activation on planar lipid bilayers. Overall, stimulation of different macrophage types through Fc receptors was found to trigger significant EV secretion.

### 3.3 Secreted extracellular vesicles are retained in situ following macrophage removal

**FIGURE 3:**
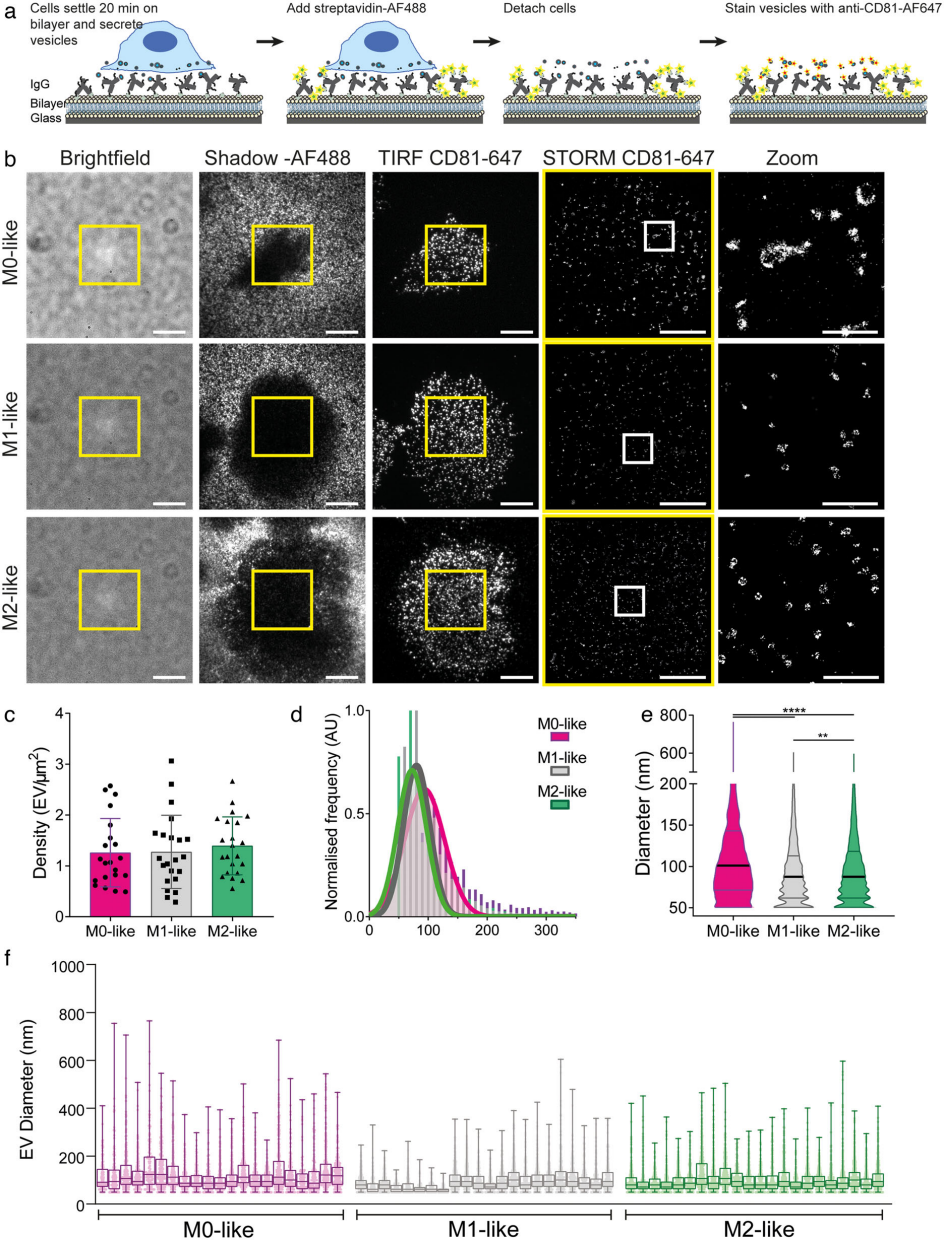
Macrophage secreted EVs are retained in situ after cellular removal. (a) Macrophages were incubated on IgG‐containing lipid bilayers for 20 min, pulse stained with streptavidin‐AF488, detached, fixed and stained with anti‐CD81 mAb conjugated to AF647. (b) Representative Brightfield images, images of the bilayer stained with AF488 showing shadows where cells have been (Shadow‐AF488), TIRF and STORM images of CD81 stained with AF647 (TIRF CD81‐647 and STORM CD81‐647) and zoomed‐in regions of the STORM image (Zoom; 3 × 3 μm). Scale bars: Brightfield and TIRF; 10 μm, STORM; 5 μm, Zoom; 1 μm. Yellow boxes indicate TIRF crop for STORM. White boxes indicate STORM Zoom. C‐E) Quantitative analysis of EVs including the vesicle density (number of vesicles per μm^2^) (c), a normalised diameter histogram (d) and violin plots of the detected diameters (e). (f) Diameters of individual EVs from each cell analysed. One dot represents one cell or one EV, *n* = 4 individual donors and experiments. Mean ± SD. **, *p* ≤ 0.01; ****, *p* ≤ 0.0001; Statistical significance assessed by Kruskal‐Wallis test

To confirm that EVs were fully detached from cells, and to analyse them in the absence of background signal from the cell membrane, we removed macrophages from the IgG‐containing lipid bilayers before imaging. Macrophages were incubated on planar lipid bilayers generated using 10 μg/ml IgG for 20 min, after which slides were pulsed with streptavidin‐AF488 (Ambrose et al., 2020). Cells were then removed using lidocaine and EDTA, leaving regions devoid of streptavidin‐AF488, which effectively appear as ‘shadows’ where cells had been positioned, and then cellular secretions were stained with anti‐CD81 mAb conjugated with AF647 (Figure 3a).

Within shadows, we observed CD81‐positive EVs, which had been retained in situ, clarifying that EVs observed in Figure 1 were secreted and did not remain attached to the cell (Figure 3b). The density of secreted EVs was similar in M0‐, M1‐ and M2‐like macrophages (1.26 ± 0.67, 1.28 ± 0.72 and 1.39 ± 0.57 EV/μm^2^ respectively; Figure 3c). These densities of EVs were 3.3 (M0‐like), 1.96 (M1‐like) and 1.59 (M2‐like) times greater than observed with the cell in place, likely reflecting the difficulty of accurately quantifying EVs with the fluorescence of the cell membrane present (Supplementary Figure S8).

All three macrophage types produced EVs of similar size, although EVs from M0‐like macrophages were slightly larger (Figure 3d and e). When we analysed the EV size distribution on a per cell basis we found that M1‐like macrophages had the lowest per‐cell variability, with a mean difference between the smallest and largest EV from each cell of 261 ± 110 nm compared to 439 ± 137 nm and 323 ± 89 nm for M0‐like and M2‐like macrophages, respectively (Figure 3f). This tighter EV size distribution was further evidenced by a lower co‐efficient of variation, 42.7 ± 8.9% for M1‐like derived EVs compared to 49.8 ± 7.4% and 58.4 ± 9.7% for M2‐ and M0‐like EVs respectively. Figure 3f identifies eight cells that drive this reduction in EV size range for M1‐like EVs, demonstrating that analysing EVs on a single‐cell basis is important for fully understanding the heterogeneity of the population.

To further test whether the CD81 rings were secreted vesicles rather than sheared cellular debris, macrophages were activated in solution using beads opsonised with human IgG. This created a suspension of EVs that we separated into large and small EVs by differential ultracentrifugation (Théry et al., 2006). Whilst it is known that EVs are generated through both direct membrane budding and multi‐vesicular bodies, producing microvesicles and exosomes respectively, there is a distinct lack of markers for these differential routes of biogenesis. We therefore describe the different fractions as large and small EVs to reflect their known physical characteristics (Théry et al., 2019). Each fraction was added to PLL coated slides and stained for CD81 which confirmed that large and small EVs had been separated effectively (Supplementary Figure S9).

The retention of EVs in situ on planar lipid bilayers following the removal of macrophages raises the question of how they are retained. To study this, supernatants containing EVs were generated using differential centrifugation to remove cells and cell‐debris before adding them to chambered coverslips coated with 0.01% PLL, protein‐rich lipid bilayers containing IgG, BSA or 0.01% PLL with anti‐CD9, anti‐CD63 and anti‐CD81 mAbs. The use of anti‐tetraspanin mAbs would specifically capture EVs and serve as a positive control for EV retention. When compared to the anti‐tetraspanin capture, BSA prevented almost all EV capture (8 ± 4%) while PLL alone captured 78 ± 19% of the EVs. IgG‐containing lipid bilayers were able to capture 89 ± 42% of the EVs suggesting this surface is capable of capturing most EVs (Supplementary Figure S10A). There was no difference in the size profile of the EVs captured on any of the surfaces, suggesting that sub‐types of EVs were not selectively captured across these conditions (Supplementary Figure S10B). We also tested whether washing lipid bilayers with a PBS solution containing 10 mM lidocaine hydrochloride and 0.5 mM EDTA would remove significant numbers of EVs. However, we found that there was no significant difference between the number and size profile of EVs captured by washed and unwashed lipid bilayers (Supplementary Figure S10C and D).

In contrast, we found that the number of EVs retained on bilayers was affected by incubation at an acidic pH. Briefly, M1‐like macrophages were incubated on IgG‐containing lipid bilayers before being detached, and the resulting secretions were incubated in pH calibrated ddH_2_O at pH 7, 6, 5 or 4 for 1 h at RT. As the pH became more acidic, the number of EVs that were retained on bilayers decreased, but this did not affect the size of the retained EVs (Supplementary Figure S10E and F). Together, these data indicate that EVs adhere to ligand‐rich planar lipid bilayers following secretion and macrophage detachment. However, EVs can be removed using an acidic pH. Overall, these data demonstrate that the EVs are secreted by macrophages and that they can be retained in situ on lipid bilayers for analysis of individual EVs on a per‐cell basis.

### 3.4 EVs are enriched at holes in the actin mesh and cambinol inhibits their secretion

**FIGURE 4:**
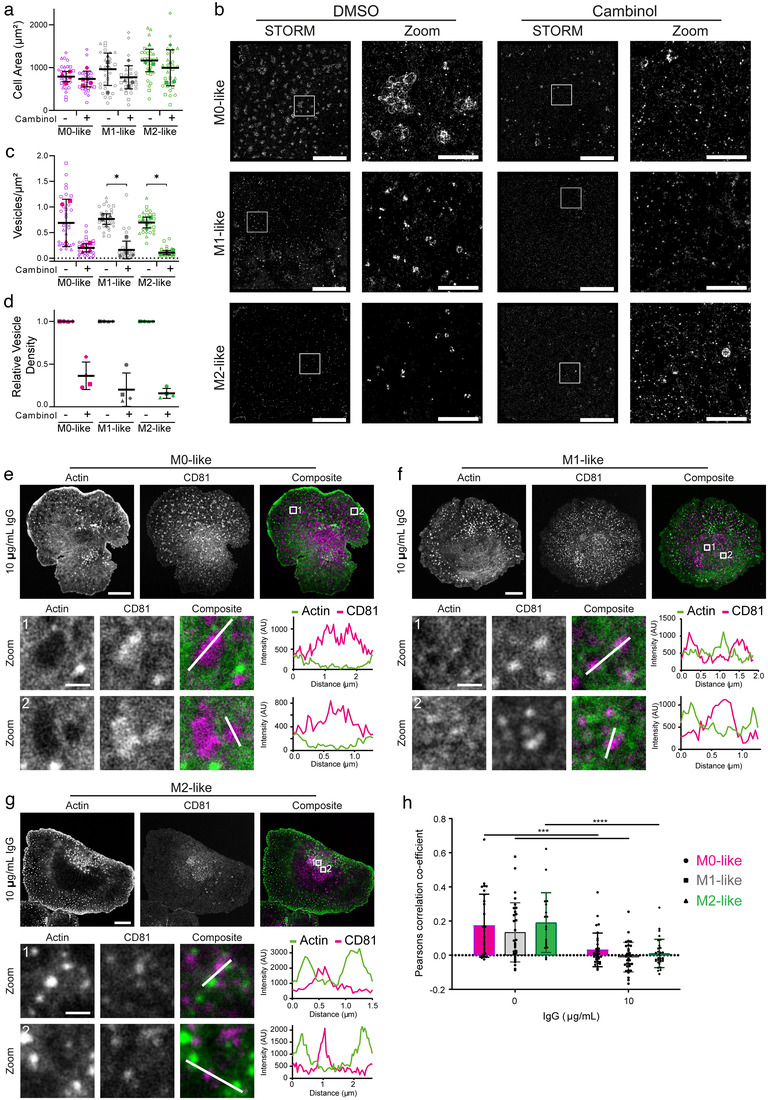
Extracellular vesicles colocalise with holes in the actin mesh and can be inhibited by cambinol. (a–d) Macrophages were treated for 120 min with either 2 μM Cambinol (+) or DMSO (−), incubated for 15 min on glass slides coated with 0.01% PLL and 10 μg/ml IgG, fixed, blocked and stained with anti‐CD81‐AF647 mAb. (a) Area of spread cells. (b) Representative STORM and Zoom (5 × 5 μm) images (Scale bar; 5 μm, Zoom; 1 μm, White boxes; STORM Zoom). (c) Vesicle density defined as the number of vesicles per μm^2^. (d) Vesicle density relative to control condition from matched donors. Open dots represent single cells and closed dots represent mean donor values. *n* = 4 individual donors and experiments, mean ± SD. (e–h) Macrophages were plated on glass slides coated with 0.01% PLL only (0 μg/ml IgG) or 0.01% PLL and IgG (10 μg/ml IgG) for 15 min, fixed, permeabilised, blocked and stained with anti‐CD81‐AF647 mAb and phalloidin‐AF488. The actin mesh was imaged with STED and CD81 using confocal microscopy. Shown are representative images and composites with two indicated zoom regions (3 × 3 μm) with matched line profiles for M0‐like (e), M1‐like (f) and M2‐like (g) macrophages (Scale bars; 10 μm, Zoom; 1 μm, White boxes; Zoomed region). (h) Pearson correlation co‐efficient for staining of actin and CD81. *n *= 3 individual donors and experiments. Each dot represents one cell. Mean ± SD. *, *p* ≤ 0.05; ***, *p* ≤ 0.001; ****, *p* ≤ 0.0001; Statistical significance assessed by unpaired *t* test (A, C, H)

Having observed that macrophages secrete EVs following stimulation with IgG we next investigated their mechanism of release. Larger EVs, such as microvesicles, are secreted following direct budding from the membrane, but smaller vesicles, like those observed here, are more characteristic of exosomes. A key mechanism of exosome biogenesis is the hydrolysation of sphingomyelin to produce ceramide that leads to EV formation in an endosomal sorting complex required for transport (ESCRT) independent process (Trajkovic et al., 2008). Sphingomyelin hydrolysation is controlled by neutral sphingomyelinases, so we set out to inhibit neutral sphingomyelinase 2 (nSMase2) using cambinol (Figuera, 2015). Macrophages were incubated with cambinol (2 μM) for 2 h, plated onto IgG‐coated slides for 15 min and then stained with anti‐CD81 mAb. Importantly, inhibition with cambinol at this concentration did not inhibit cell spreading, suggesting that the inhibition of EV release was not due to off‐target effects on early cellular activation processes (Figure 4a). STORM revealed that EV release was substantially reduced following incubation with cambinol (Figure 4b). We observed significant decreases in EV density at the membrane for M1‐ and M2‐like macrophages (Figure 4c), although this change was masked for M0‐like macrophages until they were normalised for donor variability (Figure 4d). The extent of the reduction following incubation with cambinol suggests that EVs are predominantly secreted from macrophages via an nSMase2‐dependent mechanism.

Under the cell surface membrane, cytoskeletal rearrangement has been shown to be critical for vesicle and granule permissibility (Carisey et al., 2018; Catalano & O'Driscoll, 2020). Therefore, imaging the synaptic actin cytoskeleton using super‐resolution STED microscopy, alongside CD81, knowing that bright spots correspond to EVs, allowed examination of the interplay between EVs and the cortical actin mesh (Figure 4e–g). Line profiles of actin and CD81 fluorescence on IgG activated M0‐, M1‐, and M2‐like macrophages display an alternating signal between actin and CD81, suggesting that EVs are distinctly localised from the actin cytoskeleton. Furthermore, a significant reduction in CD81 and actin co‐localisation occurs following activation (Figure 4h). These data demonstrate that active remodelling of the actin cortex occurs following macrophage activation and that EVs are present in the gaps created. This indicates a commonality with the cytoskeletal processes involved in the secretion of lytic granules at cytolytic immune synapses (Brown et al., 2011; Carisey et al., 2018; Rak et al., 2011).

### 3.5 Proteomic analysis of EVs released by distinct macrophage subtypes

**FIGURE 5:**
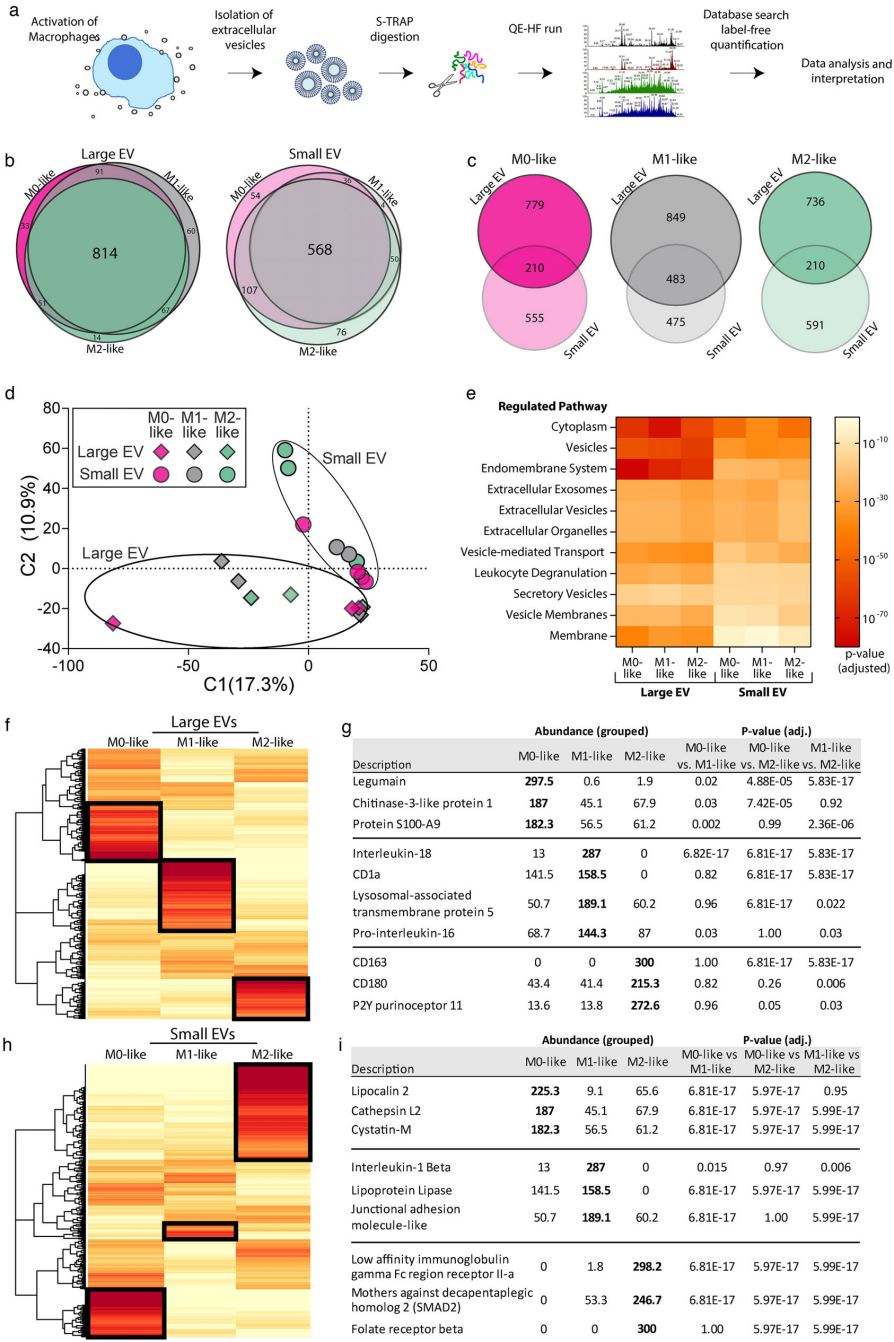
Proteomic analysis of extracellular vesicles reveals distinct contents for different macrophages. (a) Monocyte‐derived macrophages were stimulated in solution with IgG‐coated beads for 1 h to stimulate EV secretion. Large and small EVs were isolated via differential ultracentrifugation, digested, analysed by mass spectrometry and label free quantification performed. (b,c) Proportional Venn diagrams showing the number of shared proteins between the different macrophage types (b) or different vesicle types (c). (d) Principal component analysis of all identified proteins in each sample. (e) Heatmap of selected significantly regulated pathways, identified using a weighted query in G:Profiler, from large and small EV, colour coded for their adjusted p‐value as indicated. (f–i) Heatmaps of all significantly regulated proteins with tables highlighting selected proteins for large EVs (f–g) and small EVs (h–i). *n* = 3 individual donors and experiments for all panels

To identify proteins which were differentially expressed on EVs secreted from different macrophage subtypes, we profiled the proteome of EVs secreted from M0‐, M1‐ and M2‐like macrophages activated in solution via IgG‐coated beads. In brief, macrophages were incubated with IgG‐coated beads for 1 h and secreted EVs were isolated by differential centrifugation. Cells were first removed (at 350 × *g*), followed by cell‐debris (2000 × *g*), large EVs (100,00 × *g*) and finally small EVs (100,000 × *g*). EV pellets were then subjected to digestion and proteomic analysis (Figure 5a). To a large extent, all small EVs or all large EVs expressed many of the same proteins, regardless of the macrophage type they were derived from (Figure 5b). However, for each type of macrophage, protein contents were dramatically different between large and small EVs (Figure 5c). This was further evidenced through a principal components analysis that distinguished large and small EVs using the first two components with further clustering generated by donor variability (Figure 5d).

When all proteins from each EV population were subjected to a pathway analysis, weighted on protein abundance, the pathways that contained the highest proportion of regulated proteins were predominantly involved in the secretion of vesicles (Figure 5e). By considering the amount of each protein expressed on M0‐, M1‐ or M2‐like macrophage EVs, relative to the total from all three, a group of proteins that were significantly upregulated for each macrophage subtype were identified (Figure 5f and h). For example, large EVs from M1‐like macrophages were associated with IL‐18, a pro‐inflammatory cytokine, Pro‐IL‐16, a CD4^+^ immune cell chemoattractant (Center & Cruikshank, 1982), CD1a, a Class I MHC related protein, and lysosomal‐associated protein transmembrane five, a positive modulator of inflammatory pathways in macrophages (Glowacka et al., 2012). Large EVs derived from M2‐like macrophages were associated with the M2 markers, CD163 and CD180, and the purinoceptor P2Y11 that is involved in resolving inflammation (Gruenbacher et al., 2021) (Figure 5g). We found that small EVs from M0‐like macrophages were enriched with Lipocalin 2, a macrophage polarisation regulator (Guo et al., 2014), while M1‐like macrophage small EVs were associated with the inflammatory mediator IL‐1β and the junction adhesion molecule‐like protein, which mediates the migration and adhesion of immune cells to regulate inflammation (Fang et al., 2021). With small EVs from M2‐like macrophages we observed significant enrichment of the activating Fc receptor, low affinity immunoglobulin gamma Fc region receptor II‐a, the TGFβ mediating protein, SMAD2 and folate receptor beta, which is an M2 polarisation marker (Puig‐Kröger et al., 2009) (Figure 5i). Overall, the proteomic analysis demonstrated significant differences in protein profiles between large and small EVs from each type of macrophage. In addition, EVs from different macrophage types incorporated discrete signature proteins.

### 3.6 In situ analysis of single cell secretions finds M1‐like EVs enriched in HLA‐DR

**FIGURE 6:**
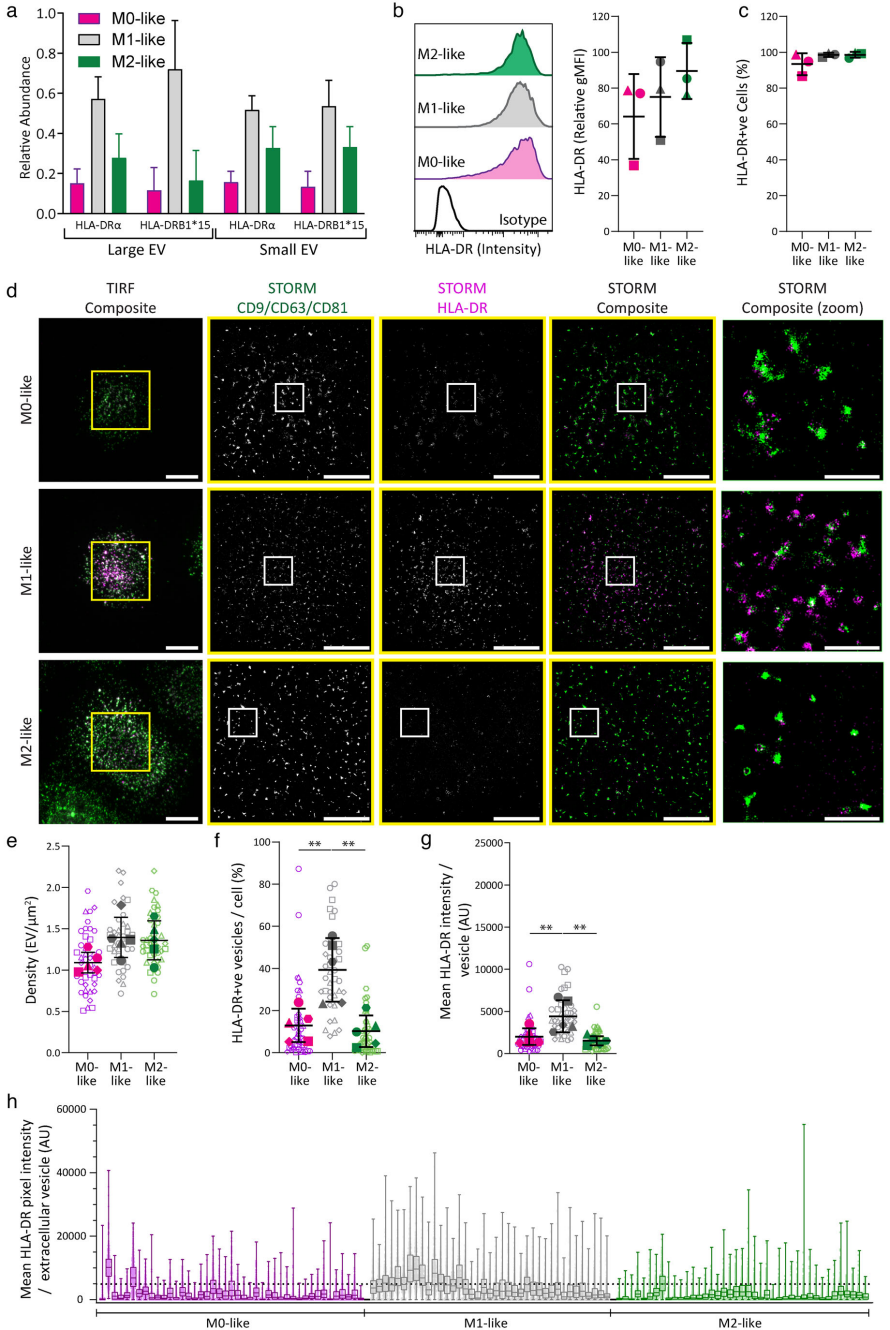
EVs secreted by M1‐like macrophages display higher levels of MHC class II protein. (a) Relative expression of HLA‐DRα and HLA‐DRB1*15 across macrophage subtypes in small and large EVs as measured by proteomic analysis. *n* = 3 individual donors and experiments, mean ± SD. (b–c) Differentiated macrophages were assessed by flow cytometry for cell surface expression of HLA‐DRα, with the relative gMFI indicated (b) and percentage of positive cells (c). *n* = 3 individual donors and experiments, mean ± SD. D‐H) M0‐, M1‐ and M2‐like macrophages were incubated on planar lipid bilayers containing IgG from human serum for 20 min, detached, fixed, blocked and stained with anti‐CD9‐AF488 mAb, anti‐CD63‐AF488 mAb, anti‐CD81‐AF488 mAb and anti‐HLA‐DR‐AF647 mAb. (d) Representative images of secreted EVs visualised using STORM (Scale bars; 5 μm, Zoom; 1 μm, Yellow boxes; TIRF crop for STORM, white boxes; STORM Zoom). (e) Density of vesicles secreted from each cell analysed. (f) Percentage of HLA‐DRα‐positive vesicles per individual cell. (g) Mean pixel fluorescence intensity of HLA‐DRα on individual EV, summarised for each cell. (h) Box and whisker plots displaying the mean pixel intensity of HLA‐DR on each individual vesicle, separated as profiles for individual cells with median and quartiles indicated. (e–g) Open symbols represent single cells, with filled symbols showing each donor's mean, *n* = 5 individual donors and experiments, mean ± SD. **, *p* ≤ 0.01; Statistical significance assessed by one‐way ANOVA (E–G)

Bulk proteomic analysis of macrophage EVs demonstrated that each subset upregulated a unique group of proteins. However, this type of analysis is unable to reveal EV heterogeneity between individual cells or the EV profile within an individual cell. In the proteomic analysis, the MHC class II protein subunit, HLA‐DRα, was enriched in both small and large EVs from M1‐like macrophages, with higher abundance in large EVs (Figure 5a). HLA‐DRα associates with HLA‐DRβ, but due to extensive polymorphism in HLA‐DRβ, mapping alleles using proteomics is challenging. However, several HLA‐DRβ variants had increased expression in small and large EVs derived from M1‐like macrophages. One variant expressed by all three donors tested, HLA‐DRB1*15, showed increased expression on small and large EVs from M1‐like macrophages (Figure 6a). Interestingly, cell membrane expression, assessed by flow cytometry, suggested that almost all macrophages expressed HLA‐DR, and variation in expression was greater between donors than between macrophage subtypes (Figure 6b and c). Thus, demonstrating that EV expression is not merely a direct reflection of surface membrane expression.

To visualise HLA‐DR on vesicles, macrophages were activated on IgG‐containing planar lipid bilayers for 15 min, cells were detached, and samples blocked before staining using anti‐CD9‐AF488, anti‐CD63‐AF488 and anti‐CD81‐AF488 to ensure comprehensive coverage of the EV tetraspanins alongside anti‐HLA‐DR‐AF647. EVs secreted from individual cells were visualised using STORM microscopy (Figure 6d). M1‐ and M2‐like macrophages secreted the highest density of EVs per μm^2^, 1.39 ± 0.24 and 1.36 ± 0.24 EV/μm^2^ respectively, which was more than M0‐like macrophages, 1.09 ± 0.12 EV/μm^2^ (Figure 6e).

EVs larger than 60 nm were identified using CD9, CD63 and CD81 staining and HLA‐DR expression at these sites was assessed. HLA‐DR was present on EVs secreted by all macrophage types, but its expression was higher and more frequent on secretions from M1‐like macrophages (Figure 6d and Supplementary Figure S11). M1‐like macrophages had a significantly higher percentage of HLA‐DR^+^ EVs per cell (39 ± 15%) compared to M0‐ and M2‐like macrophages; 13 ± 8 and 10 ± 7% respectively (Figure 6f). Also, the mean pixel intensity of HLA‐DR from individual EVs was significantly increased in M1‐like macrophages compared to M0‐ and M2‐like macrophages (Figure 6g). HLA‐DR intensity was measured as the mean pixel intensity across each EV rather than the sum of HLA‐DR expression on each EV to ensure that EV size did not affect the measurement. When we stratified HLA‐DR expression on EVs by individual cells we found that while the expression on M0‐ and M2‐like EVs was low overall, there were a minority of EVs from every cell that had high HLA‐DR expression. Moreover, there was considerable heterogeneity between individual cells. For example, a minority of cells within the M0‐like macrophage population secreted EVs with HLA‐DR expression profiles like those of M1‐like macrophages (Figure 6h).

### 3.7 Human lung macrophages secrete EVs following activation

**FIGURE 7:**
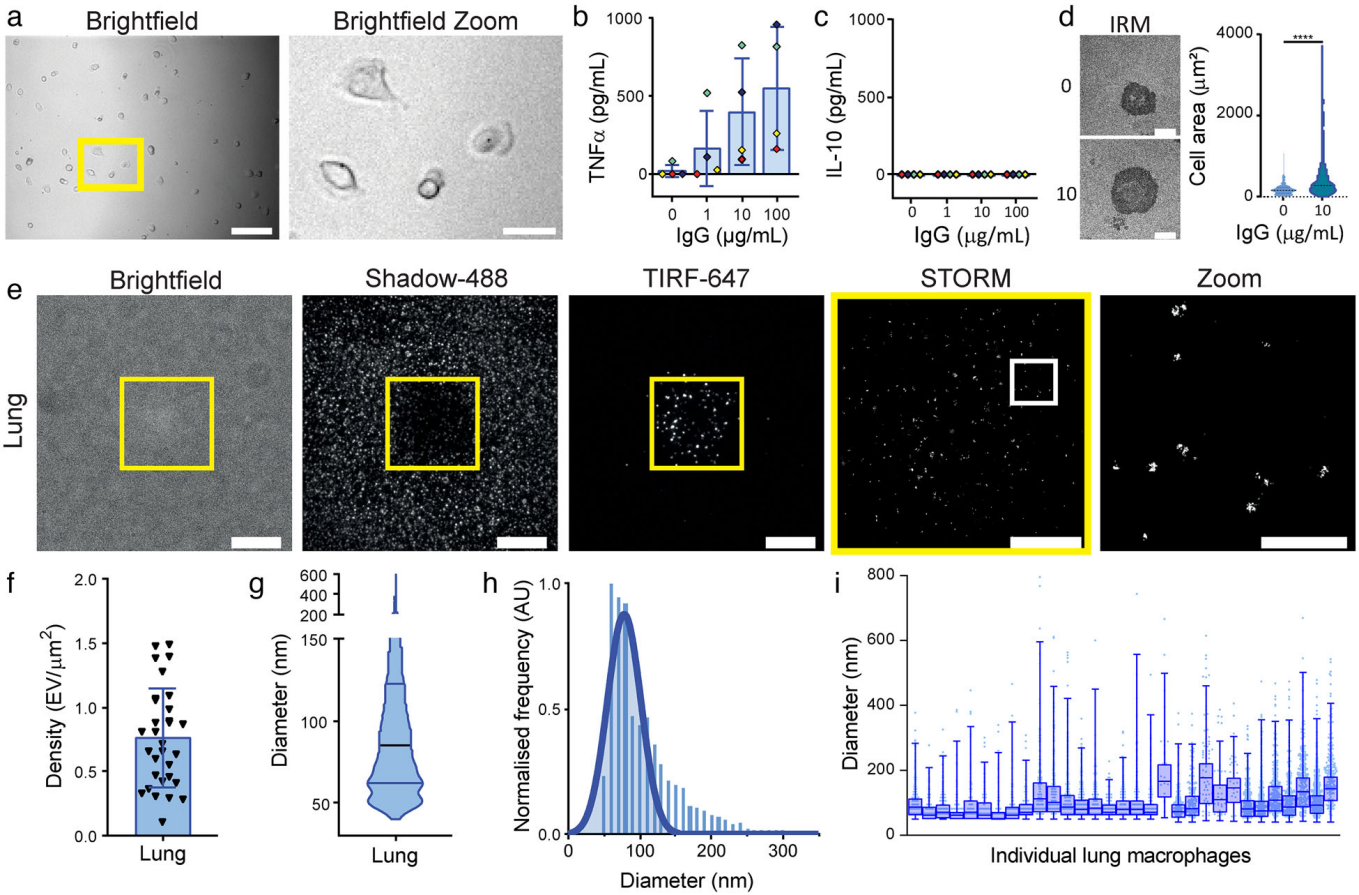
Human lung macrophages secrete EVs upon activation through their FcγRI. Lung macrophages were isolated from human lung resections and selected for by adherence. (a) Representative widefield images of lung macrophages (Scale bars; 100 μm, Zoom; 30 μm, Yellow box; Zoomed region). (b–d) Macrophages were incubated on glass slides coated with 0.01% PLL and indicated concentrations of IgG from human serum for 20 h and supernatants were analysed for TNFα (b) and IL‐10 (c) by ELISA. *n* = 4 individual donors and experiments, mean ± SD. (d) Representative IRM images and corresponding cell contact area quantification. (e–i) Macrophages were incubated on IgG‐containing lipid bilayers for 20 min, pulse stained with streptavidin‐AF488, detached, fixed and stained with anti‐CD81‐AF647. *n* = 4 individual donors and experiments, mean ± SD. (e) Representative Brightfield, TIRF AF488 (Shadows), TIRF AF647, STORM and Zoom (3 × 3 μm) images (Scale bars: Brightfield and TIRF; 10 μm, STORM; 5 μm, Zoom; 1 μm, Yellow boxes; TIRF crop for STORM, white boxes; STORM Zoom). (f) Secreted EV density. (g) EV diameter violin plot. (h) Normalised EV diameter histograms. (i) Diameters of every EV for individual lung macrophages, displayed as box and whisker plots with median and quartiles indicated. **** **, p ≤ 0.0001; Statistical significance assessed by unpaired t test

Research on human macrophages is predominantly carried out using monocyte‐derived macrophages in vitro due to the difficulty accessing tissue resident macrophage populations in humans. While every effort is taken for monocyte‐derived macrophages to recapitulate the macrophages found in vivo, tissue‐derived macrophages are likely to have specific properties which vary from their blood‐derived counterparts. Therefore, using lung macrophages from non‐malignant tissue, proximal to lung tumour resections, we could compare our monocyte‐derived macrophage results to a physiologically relevant tissue‐resident macrophage population. We separated lung macrophages from other lung‐derived cells using adherence and imaged them to characterise their morphology. In the absence of activation, lung macrophages were large and rounded (Figure 7a). Lung macrophages could not be analysed by flow cytometry on account of having high autofluorescence due to their exposure to environmental contaminants, and thus we used functional assays to characterise these cells. In response to 20 h incubation on IgG‐coated glass slides, lung macrophages secreted TNFα in a dose‐dependent manner but did not secrete IL‐10 (Figure 7b and c). The activation of lung macrophages in response to Fc receptor ligation was further confirmed by measuring their spreading response following incubation on IgG coated glass slides for 15 min. Activation led to a significant increase in lung macrophage contact area, as measured by IRM (Figure 7d). Lung macrophages spread similarly to monocyte‐derived macrophages without activation. However, their increase in contact area following activation was not as large as monocyte‐derived macrophages (Figure 1d).

We next assessed EV secretion from lung macrophages following 20 min incubation on lipid bilayers containing IgG from human serum. After generating cell shadows with streptavidin‐AF488, we detached the cells and stained for CD81^+^ EVs (Figure 7e). Lung macrophage EVs had a density of 0.89 ± 0.44 EV/μm^2^ (Figure 7f), which was lower than that observed for M0‐, M1‐ and M2‐like blood‐derived macrophages (Figure 3c). The mean diameter of lung macrophage EVs was 101 ± 57 nm (Figure 7g) and the modal diameter was 93 ± 50 nm (Figure 7h), which was similar to monocyte‐derived macrophages (Figure 3d and e). When size distribution was assessed on a per cell basis, lung macrophages were found to be highly variable (Figure 7i) with one subset of cells specialised in secreting a few large EVs. Thus, EV secretion can be used to define subsets of tissue macrophages.

### 3.8 Comparing the EV profiles of monocyte‐derived and lung‐derived macrophages

**FIGURE 8:**
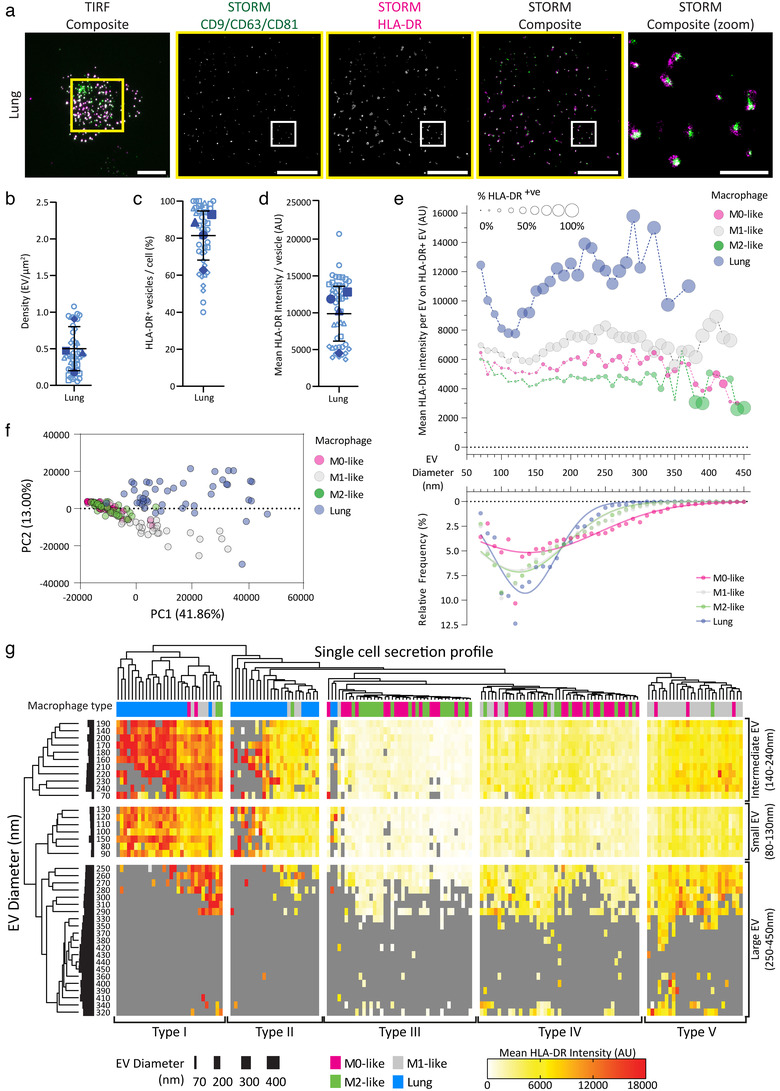
Comparing the EV profiles of individual macrophages. (a–d) Lung macrophages were incubated on IgG containing planar lipid bilayers for 20 min, detached, blocked and stained with anti‐CD9‐AF488 mAb, anti‐CD63‐AF488 mAb, anti‐CD81‐AF488 mAb and anti‐HLA‐DR‐AF647 mAb. *n* = 4 individual donors and experiments, mean ± SD. (a) Representative STORM images of secreted EVs (Scale bars; 5 μm, Zoom; 1 μm, Yellow boxes; TIRF crop for STORM, White boxes; STORM Zoom). (b) Density of EVs secreted from each cell. (c) Percentage of secreted EVs expressing HLA‐DR per cell. (d) Mean fluorescence intensity of HLA‐DRα on individual EVs, summarised for each cell. (e) Histogram with EV diameter binned by 10 nm, showing the effect of EV size on HLA‐DR intensity and the percentage of HLA‐DR positive EVs, comparing different macrophage subtypes. (f) Principal components analysis resolving the heterogeneity of individual EVs for each individual cell with macrophage subtype indicated. (g) A heatmap showing individual cell expression of HLA‐DRα on EVs of different sizes with unbiased hierarchical clustering of individual cells and EV diameter bins (representative of 168 cells from four (lung) or five individual donors)

We next imaged the secretions of lung macrophages incubated on IgG‐containing bilayers and directly visualised HLA‐DR expression (Figure 8a). Lung macrophages secreted EVs at a density of 0.50 ± 0.30 EV/μm^2^ (Figure 8b), which was fewer than seen in corresponding experiments with M0‐, M1‐ and M2‐like macrophages (Figure 6e). However, HLA‐DR was very highly expressed on lung macrophage EVs, with 81 ± 13% of EVs positive for HLA‐DR (Figure 8c), substantially higher than the HLA‐DR enriched EVs of M1‐like macrophages (39 ± 15%; Figure 6f). Further to this, the intensity of HLA‐DR expression on lung macrophage EVs was very high (Figure 8d), over two‐fold greater than EVs from M1‐like macrophages and five times the mean level on EVs from M0‐ and M2‐like macrophages (Figure 6g). These data indicate that lung macrophages secrete similarly sized EVs to monocyte‐derived macrophages but lung macrophage EVs are less numerous and substantially more enriched in MHC class II protein than any monocyte‐derived macrophages.

To further compare lung macrophage EVs to those secreted by monocyte‐derived macrophages we stratified the lung data using 10 nm bins for EVs (Figure 8e). This analysis revealed that larger EVs (>140 nm) were more likely to express HLA‐DR (larger plot symbols), matching the proteomic analysis (Figure 6a). In addition, the mean intensity of HLA‐DR on EVs expressing this protein, was higher on M1‐like and lung macrophages than HLA‐DR positive EVs from M0‐ and M2‐like macrophages. The intensity of HLA‐DR on the largest EVs (>250 nm) was variable, but these had low abundance (Figure 8e). Overall, this data demonstrates that M0‐, M1‐ and M2‐like, as well as lung macrophage EVs have disparate characteristics, with variance in size, frequency and HLA‐DR expression. A principal component analysis (PCA) examining single cell EV profiles showed M0‐ and M2‐like macrophages grouping tightly together with M1‐like macrophages exhibiting a comparatively distinct phenotype (Figure 8f). Interestingly the lung macrophages integrate into this analysis as a separate population, distinct from the M0‐/M2‐like group and the M1‐like macrophages, with both M1‐like and lung macrophages demonstrating the broadest variance.

Analysing the mean HLA‐DR expression of EVs on a per cell secretion basis revealed significant variation from cell‐to‐cell. However, our in situ analysis provided even more detailed information on a per EV basis. Therefore, we analysed whether the expression and intensity of HLA‐DR varied with EV size on individual cells and compared this between macrophage phenotypes (Figure 8g and Supplementary Figure S12). Hierarchical clustering based on HLA‐DR expression identified three groups of EV diameters. The smallest group comprised vesicles with diameters between 80 and 130 nm with lower HLA‐DR expression compared to the rest of that cell's overall EV population. An intermediate EV population from 140 to 240 nm that had high HLA‐DR expression and a third, with all EVs larger than 250 nm, had variable HLA‐DR expression (Figure 8g).

This analysis also separated the secretion profiles of individual macrophages into five sub‐types. Lung macrophage secretions were distinct and divided into two types. Type I comprised EV profiles with the highest concentrations of HLA‐DR and Type II had intermediate HLA‐DR expression. Interestingly, Type I secretions, driven by high HLA‐DR expression, also contained a subset of monocyte‐derived macrophage secretions that were largely M1‐like. Notably, the fact that some monocyte‐derived macrophage EV profiles fit Type I secretions implies that a subpopulation of those cells secrete EVs that are highly enriched in HLA‐DR and very similar to those of lung macrophages. This suggests that there is an enrichment of a specific macrophage sub‐population, identified by its EV secretion, in the lung tissue of cancer patients.

Similar to the PCA analysis, the M0‐ and M2‐like macrophage EVs were largely indistinguishable. Two sub‐types were identified, however, Type III, with almost zero or sporadic HLA‐DR expression, and Type IV with low HLA‐DR expression across all EV diameters. Finally, Type V secretions comprised the majority of M1‐like macrophage secretions forming a homogenous group with consistent intermediate HLA‐DR expression on the smallest EVs and high HLA‐DR expression on all larger EVs.

To avoid potential bias, we repeated the characterisation of M0‐, M1‐, M2‐like and lung secretions in a second, blinded version of the same experiment (Supplementary Figure S13). All samples were mixed and coded to ensure that the person conducting the experiment did not know the origin of each sample. From this analysis, monocyte‐derived macrophages secreted more EVs than lung macrophages, with M1‐ and M2‐like macrophages secreting the most (Supplementary Figure S13A), comparable to the first cohort (Figure 6e and 8b). M1‐like macrophages secreted EVs that frequently expressed HLA‐DR (52 ± 15%), which was over twice the frequency of those secreted by M0‐ and M2‐like macrophages (17 ± 16% and 10 ± 8%, respectively). Lung macrophages secreted substantially more HLA‐DR‐positive EVs than all monocyte‐derived macrophages (80 ± 9%; Supplementary Figure S13B), closely matching our initial findings (Figures 6f and 8c). Moreover, the mean intensity of HLA‐DR expression on EVs was comparable to initial findings, with M1‐like EVs expressing over double the amount of HLA‐DR found on M0‐ and M2‐like EVs. Lung macrophage‐derived EVs expressed double the level of HLA‐DR observed on EVs secreted by M1‐like macrophages (Supplementary Figure S13C).

When this dataset was stratified by EV diameter using 10 nm bins we were again able to compare single cell secretion profiles. A PCA tightly clustered M0‐ and M2‐like single cell secretions, with M1‐like secretions largely separating from this population while lung macrophages had a largely distinct phenotype (Supplementary Figure S13D). Combining all data obtained from the blinded and un‐blinded experiments again identified five secretion subtypes that matched those from Figure 8g, with each EV type comprising samples from each cohort (Supplementary Figure S13E). There was one further subtype identified from this blinded analysis: a group of lung macrophages from a single patient sample that displayed extremely high HLA‐DR expression on all EVs detected. Overall, this demonstrates that the macrophage phenotype is a major driver for the EVs secreted, which reflects known differences in the function of their parental cells. However, there is clear heterogeneity within the secretions of all macrophage subtypes, which we were able to classify into distinct sub‐types that did not simply separate by commonlymacrophage phenotype.

## 4 DISCUSSION

Despite recent intensive research, many questions regarding EV composition and function remain. Here, we investigated EVs secreted from monocyte‐derived and lung macrophages. Crucially, super‐resolution microscopy enabled us to characterise individual EVs, retained within separate secretions from single cells. Guided by proteomics and super‐resolution imaging, we revealed high cell‐to‐cell and EV‐to‐EV variability in size and HLA‐DR expression and found that HLA‐DR was enriched on secretions from M1‐like macrophages, despite equivalent membrane expression on all monocyte‐derived macrophages. Human lung‐derived macrophages secreted fewer EVs than monocyte‐derived macrophages, but these EVs expressed the highest level of HLA‐DR at a much greater frequency. Single‐cell secretions could be classified into five distinct sub‐types that demonstrated the heterogeneity of EV secretions between and within different macrophage phenotypes.

A persistent challenge in EV research is the detailed classification of EV populations, with significant work already carried out on suitable isolation methods and bulk characterisation. However, a lack of consensus remains and there is significant evidence that even minor modifications to isolation protocols can affect EV characterisation (La Shu et al., 2020; Tian et al., 2020). Here we used a minimally invasive approach to accurately quantify EV populations from single cells. Whilst the technology exists to measure physical and molecular features of EVs, many of these characteristics need to be assessed independently. For example, nanoparticle tracking analysis (NTA) rapidly provides detailed size measurements but cannot measure other features (Soo et al., 2012), while flow cytometry has limited ability to analyse the properties of the smallest EV. Fluorescent microscopy offers the opportunity to study both size and protein composition (Hisada et al., 2017; Verweij et al., 2021), however the diffraction limit restricts its application to larger EVs only. The use of super‐resolution microscopy methods, such as STORM, moves beyond the diffraction limit to resolve smaller structures, making it ideal for the simultaneous and accurate analysis of EV size and molecular composition. Accurate size quantification is key as this is frequently used to distinguish different EV populations. Nizamudeen *et al*. found EV size measurement with NTA and Tunable Resistive Pulse Sensing provided mean EV diameters approximately three times larger than STORM (Nizamudeen et al., 2018).

Here, we have taken super‐resolution microscopy and paired it with a non‐destructive approach for the study and characterisation of EVs. By activating cells and retaining EVs in situ we preserved EVs for analysis in a minimally disruptive way and allowed for their study on a single‐cell basis. The use of other techniques such as ultracentrifugation and size exclusion chromatography have their own impact on samples and lead to significantly inconsistent yields and the co‐isolation of variable contaminants from matched samples (La Shu et al., 2020; Théry et al., 2019). Indeed, ultracentrifugation has also been shown to isolate EVs with reduced functional activity compared to those isolated by size exclusion chromatography. This suggests that EV molecular composition may be altered by isolation procedures and therefore render subsequent characterisation inaccurate. Therefore, taking the approach of retaining EVs upon secretion, as demonstrated here, yields a native sample that can be accurately characterised. The mechanism by which the EVs are retained is not completely clear and may rely on charge for adherence to PLL coated glass slides or ligands in bilayers, which suggests that careful consideration needs to be given to whether the observed population reflects all EVs. However, we were able to demonstrate that the capture of EVs following secretion on bilayers is equivalent to that captured by specific mAbs which bind EV‐enriched tetraspanin proteins. We also found that EVs are tethered in a manner strong enough to resist washing, which is only perturbed by incubations at acidic pH. EV‐associated glycans may contribute to EV uptake and binding through charge and electrostatic effects (Williams et al., 2019). Also, macrophages can secrete matrix proteins (Simões et al., 2020) which may aid EV retention.

Over recent years, the field of biomedical research has moved from investigating and understanding the importance of a cell type, to subsets of those cells and onto single cells. Single cell analyses have covered cellular components from RNA to membrane proteins and while imaging has always specialised in the analysis of individual cells the study of EVs from individual cells is in its infancy. With the majority of EV analyses carried out on bulk populations, the nuance of EVs from each cell is lost in the milieu of millions of cells and with recent evidence of communication via EV secretion at the immune synapse, understanding the exchange of EVs between individual cells is of great importance (Choudhuri et al., 2014; Hoen et al., 2009; Saliba et al., 2019; Tetta et al., 2013). Microfluidic devices have been effectively employed to isolate single cells and subsequently their EVs, but are comparatively complex, only utilise low‐resolution imaging, are not applicable to all cell types and cannot directly observe the events of secretion (Ji et al., 2019; Nikoloff et al., 2021; Son et al., 2016).

Through proteomic analysis we identified significant differences between large and small EVs and also between EVs from different macrophage types. Some differentially expressed proteins matched with the known phenotypes of M1 and M2 macrophages for example, M1‐like EVs associated with pro‐inflammatory cytokines and M2‐like EVs with the M2 surface markers, CD163 and CD180. One group of proteins highlighted by the proteomic analysis of macrophage EVs was the MHC class II molecules. MHC class II molecules on EVs derived from antigen presenting cells (APCs) have previously been shown to be important for T‐cell activation (Hoen et al., 2009; Théry et al., 2019; Tkach et al., 2017; Zeng & Morelli, 2018). This T‐cell activation has been observed via multiple mechanisms, with EVs directly activating T cells or acting via EV‐mediated MHC cross‐dressing, whereby APC‐derived EVs attach to the membrane of other APCs and present MHC class II preloaded with peptide, to activate T cells (Montecalvo et al., 2008; Segura et al., 2007; Zeng & Morelli, 2018). While MHC class II positive EVs trigger weaker responses than their whole cell counterparts, they still illicit functional immune responses. Indeed, their potency is significantly increased when acting via MHC cross‐dressing on APCs (Zeng & Morelli, 2018).

The enrichment of MHC class II on M1‐like macrophage derived EVs is surprising as M0‐, M1‐ and M2‐like macrophages all expressed similar levels on their membranes. This implies that HLA‐DR expression on EVs is controlled by specific loading processes (Buschow et al., 2009; Clancy et al., 2015). The increased expression of HLA‐DR on EVs secreted by pro‐inflammatory, M1‐like macrophages matches with the function of M1 macrophages in propagating appropriate immune responses to foreign particles (Bertani et al., 2017; Hesketh et al., 2017; Vogel et al., 2014). High HLA‐DR on secretions from lung‐derived macrophages is important and could reflect their isolation from the inflammatory environment found in the lungs of cancer patients. Cigarette smoke is associated with 90% of lung cancer (Ozlü & Bülbül, 2005) and persistent cigarette smoke polarises lung macrophages to an M1 phenotype via a Wnt family member 5a dependent pathway (Feller et al., 2018). This M1 polarisation could be a driver for lung macrophages to secrete HLA‐DR positive EVs that could play a significant role in propagating pro‐inflammatory responses in the lungs of cancer patients.

The combination of super‐resolution microscopy and in situ analysis of macrophage‐derived EVs enabled us to discover the diversity of EVs and how this varied from cell‐to‐cell. Hierarchical clustering robustly defined similarities between single‐cells secretions, revealing five secretion sub‐types. Almost all M0‐ and M2‐like macrophages released EVs with similar characteristics and HLA‐DR expression, whereas the profile for M1‐like macrophage EVs was disparate and lung macrophage EVs unique again. Interestingly, ∼20% of M1‐like macrophage secretions appeared more similar to lung macrophage secretions, demonstrating the existence of a sub‐population of M1‐like macrophage EVs that are highly enriched in HLA‐DR. This also implies that a specific population of macrophages, characterised by their EV secretion are enriched, through expansion or recruitment, in the lung tissue of cancer patients.

In summary, the analysis of EVs from single cells can reveal whether subsets of cellular populations release unique sets of EVs and using super‐resolution microscopy enables us to accurately quantify multiple characteristics simultaneously. This is vital for addressing many of the remaining questions in basic EV biology and for understanding EV heterogeneity.

## CONFLICT OF INTEREST

G.M.T. is employed by G.S.K. and D.M.D. is a consultant and advisor to G.S.K.

## AUTHOR CONTRIBUTIONS

S.D., A.R.A., J.D.W. and J.M.E.L.: acquisition and analysis of data. A.R.A., S.D., G.M.T. and D.M.D.: conceived the project and interpreted data. A.R.A., S.D., J.D.W. and D.M.D.: wrote the manuscript. S.L., T.H., R.S., M.A.M. and A.M.Q. provided reagents and advice. S.D., A.R.A., J.D.W. and D.M.D.: discussed, reviewed and edited the manuscript, conceived the work and all authors approved the submitted version.

## Supporting information

Supporting information

## Data Availability

The original contributions generated in the study are included in the article/Supplementary Material, further inquiries can be directed to the corresponding author.
