# Supplementary material for: Heterogeneity in extracellular vesicle secretion by single human macrophages revealed by super‐resolution microscopy
Source: J Extracell Vesicles. 2022 Apr 12;11(4):e12215. doi: 10.1002/jev2.12215 (PMC9006015; doi:10.1002/jev2.12215)
Supplement: Supplementary file 1 — Supporting information [file JEV2-11-e12215-s001.docx]

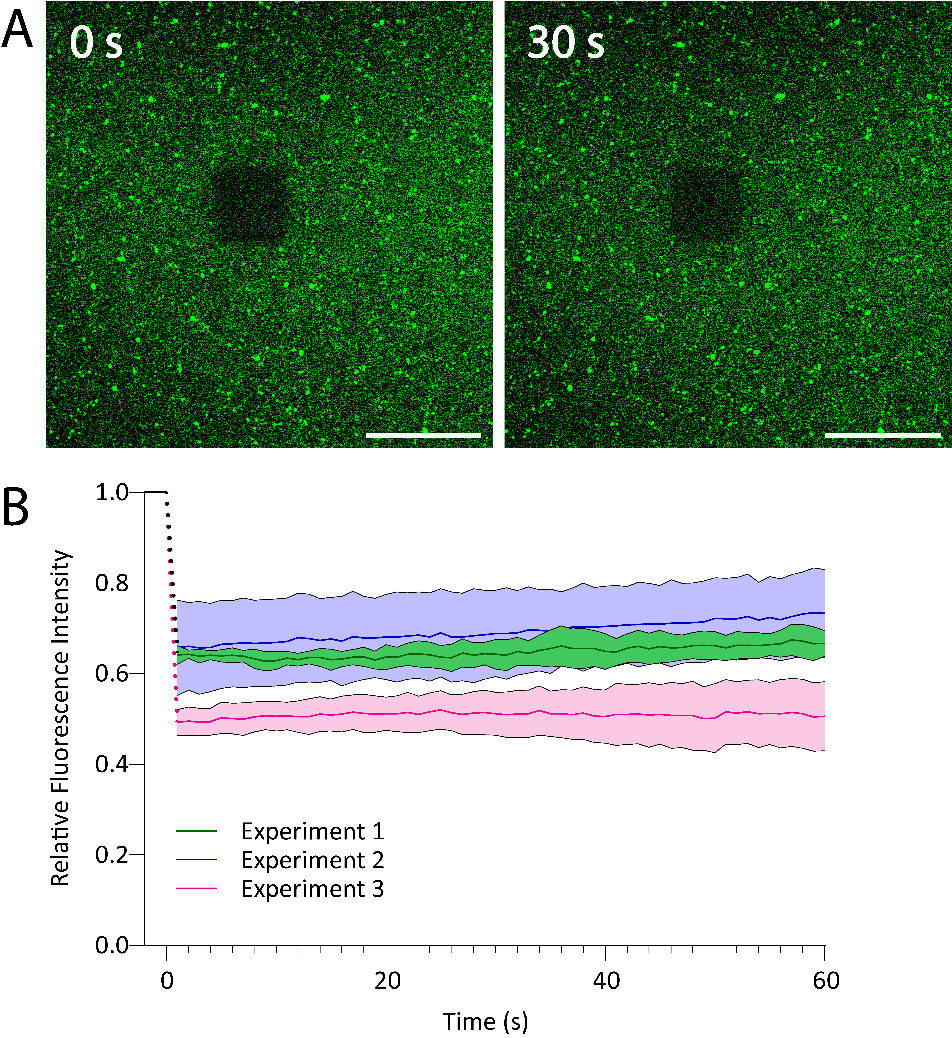


**Supplementary Figure 1: Mobility of lipid bilayers.** Fluorescence recovery after photobleaching (FRAP) experiments of AF488-stained bilayers were carried out using LAS AF FRAP wizard (Leica Microsystems). Regions of interest (ROIs) were photobleached and fluorescence intensity before and after bleaching recorded. **A)** Representative confocal images at 0 and 30 seconds (s) after bleaching (Scale bar; 5 µm). **B)** Relative fluorescence intensity (bleached vs. non-bleached regions) over time. Each coloured line represents the mean of 3 or more ROIs from a single experiment with the standard deviation indicated. n = 3 individual experiments.


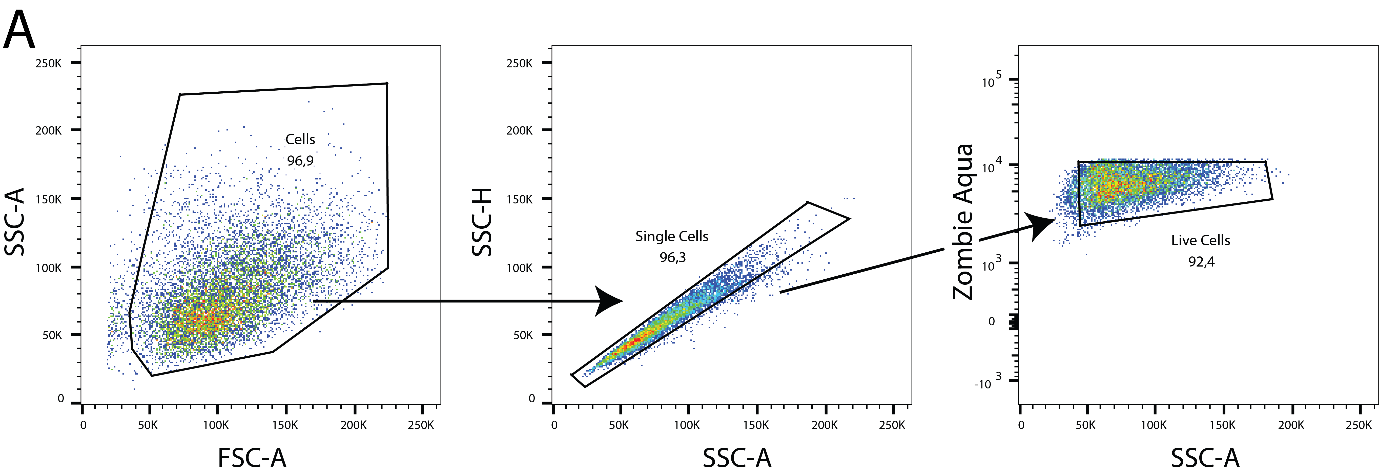


**Supplementary Figure 2:** **Gating strategy of flow cytometry experiments.** Samples were analysed using a FACS Canto II with cells identified using forward vs side scatter, then single cells selected and finally the population of live cells was identified using Live/Dead Zombie Aqua. This population of cells was then analysed using indicated antibodies.


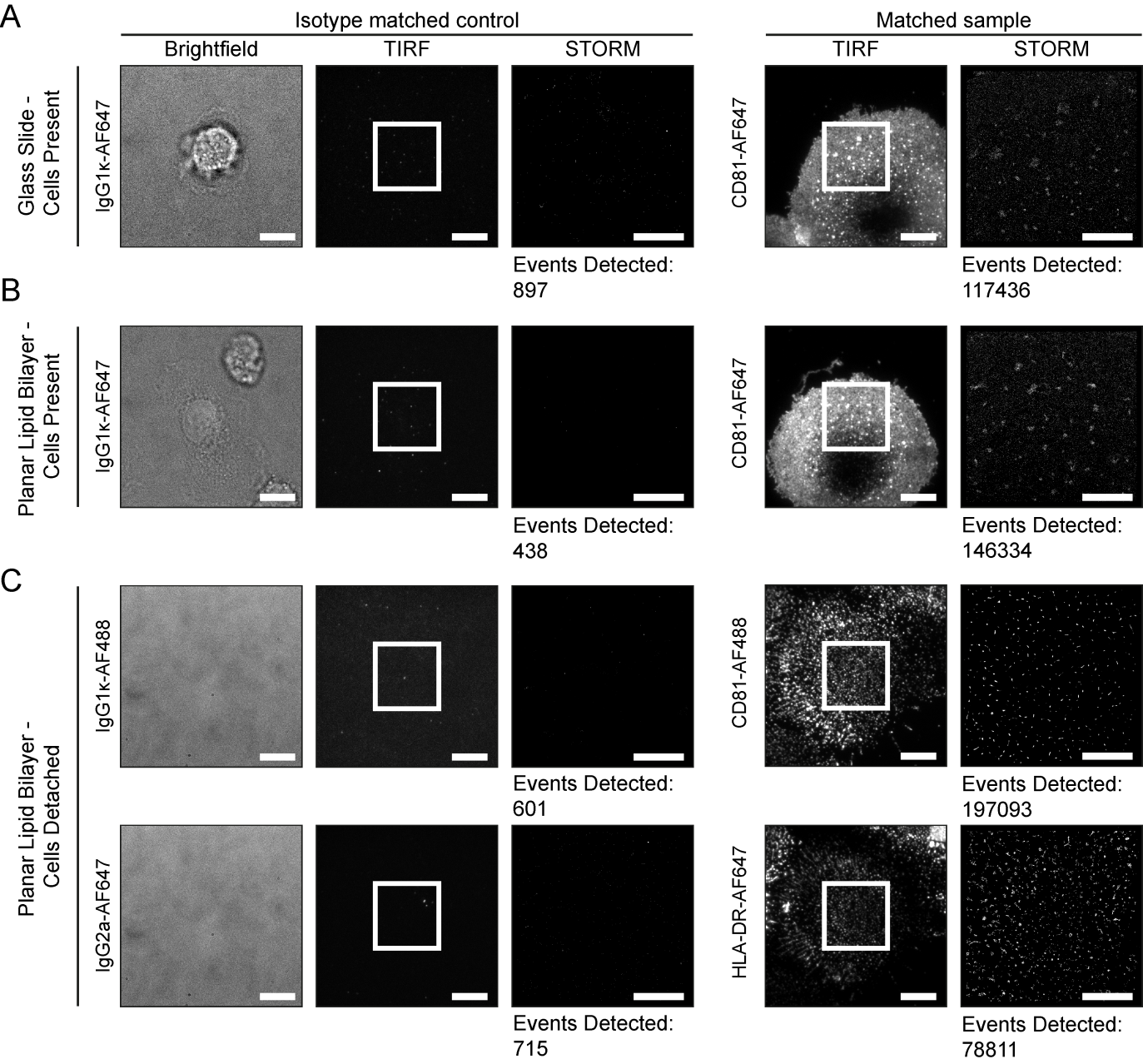
**Supplementary Figure 3: Isotype Control Imaging.** To determine the amount of signal generated by non-specific antibody binding, samples were stained with isotype matched control antibodies or matched to samples stained with CD81-AF647, CD81-AF488 or HLA-DR-AF647. Cells were activated on glass slides coated with 0.01% PLL and 10 µg/mL human IgG (A) or planar lipid bilayers generated using 10 µg/mL biotinylated human IgG with cells remaining (B) or detached (C). Representative brightfield, TIRF and STORM images are shown with the number of events detected, post reconstruction and filtering indicated (Scale bar for brightfield and TIRF; 10 µm, STORM; 5 µm).

**
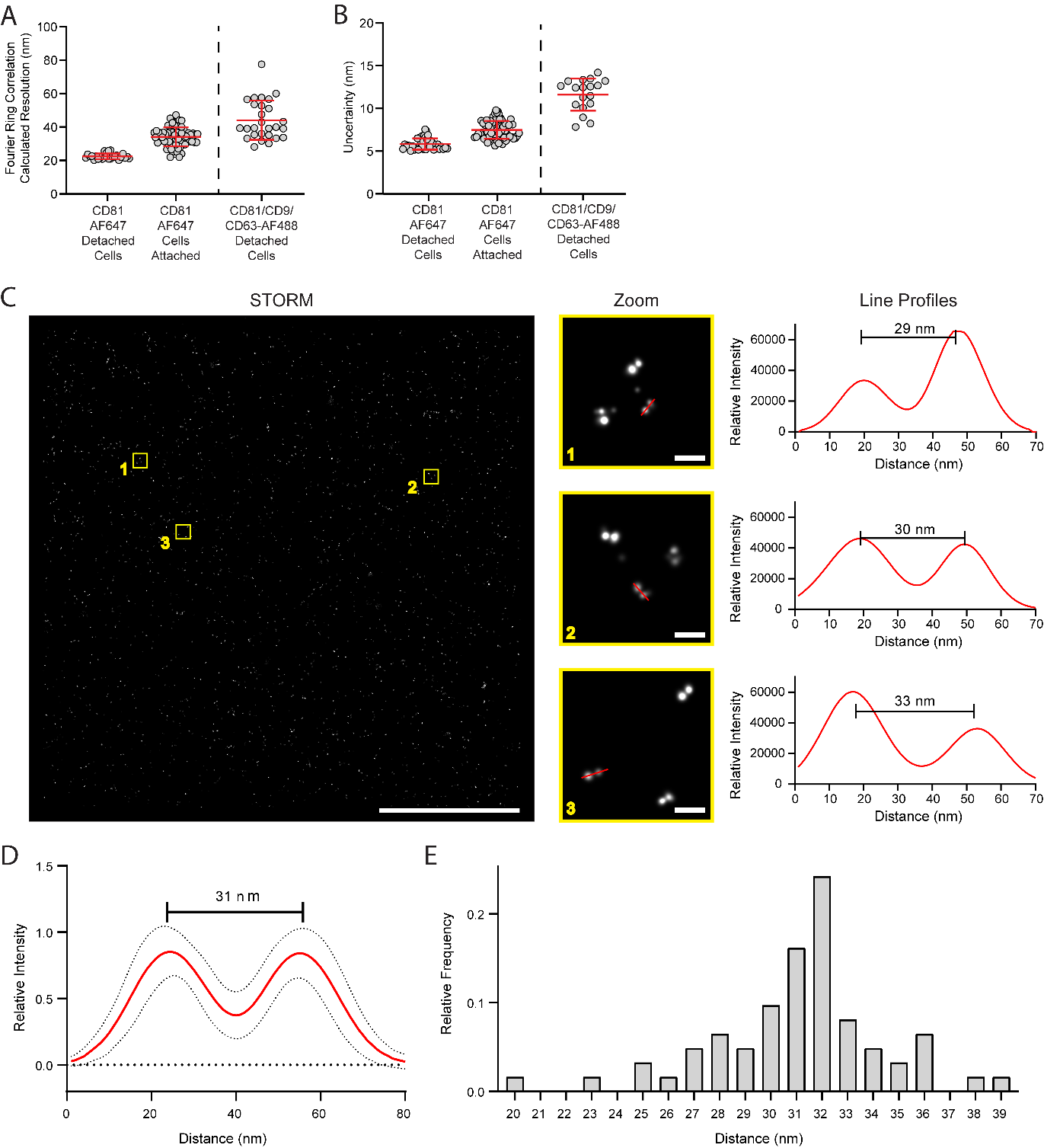
**

**Supplementary Figure 4: Calculating image resolution, localisation uncertainty and testing microscope resolution.** Background corrected datasets were split into odd and even frames from which individual events were detected and then images reconstructed and filtered. Fourier ring correlation was used to compare the degree of correlation between the two images from each dataset in a block-wise manner and determine the local resolution. A) Fourier ring correlation of multiple datasets with AF488 or AF647 conjugated antibodies with and without cells present. B) Lateral uncertainty of individual localisation from datasets calculated using the ImageJ plugin, ThunderSTORM (n = 18 – 71 datasets from individual cells). C) Representative STORM image of AF647 30nm DNA nanorulers, with 3 zoomed regions of interest and indicated line profiles from regions of interest (Scale bar – STORM; 5 µm, Zoom; 100 nm). D) Mean (red line) ± SD of 60 centrally aligned DNA nanoruler line profiles. E) Histogram of the measured length (peak to peak) of DNA nanorulers (n = 60).

**
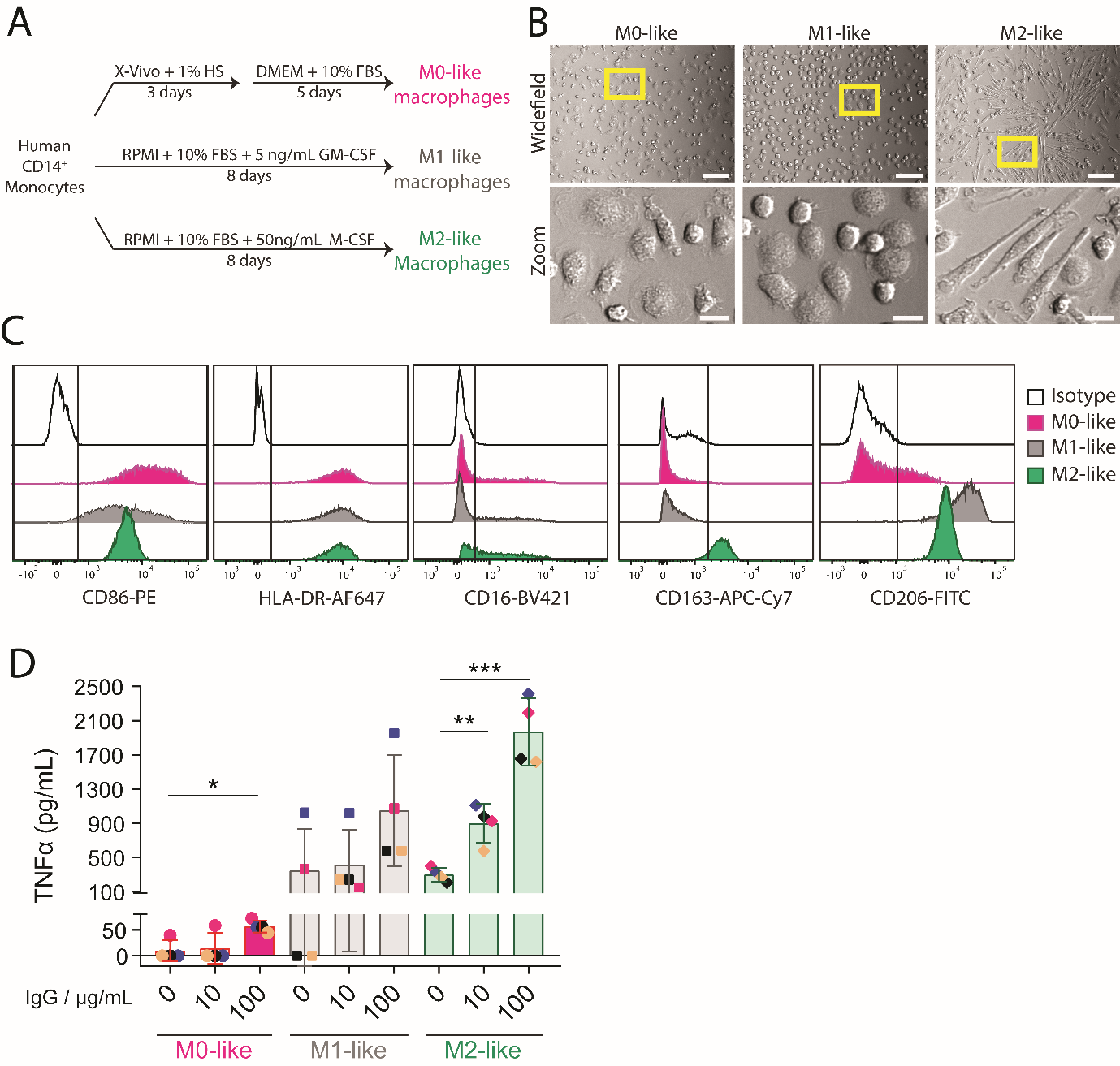
Supplementary Figure 5: Characterisation of monocyte-derived macrophages.** A) Monocytes were isolated from human blood and differentiated, as indicated, for 8 days to generate different phenotypes. B) Representative widefield images of macrophages cultured for 8 days in 6-well tissue culture plates (Scale bars; 100 µm, Zoom; 30 µm, Yellow boxes; Zoomed region). C) Flow cytometric analysis of cell surface markers on differentiated macrophages. Representative histograms from one donor of three are shown. D) M0-like, M1-like or M2-like macrophages were incubated on planar lipid bilayers generated with indicated levels of IgG for 20 h and TNFα concentrations in the supernatants were measured using Human TNFα DuoSet ELISA kits. Each dot represents 1 donor (colour coded for donors). n = 4 - 5 individual donors and experiments, mean ± SD. *, p ≤ 0.1; **, p ≤ 0.01; ***, p ≤ 0.001; Statistical significance assessed by Mann-Whitney test.


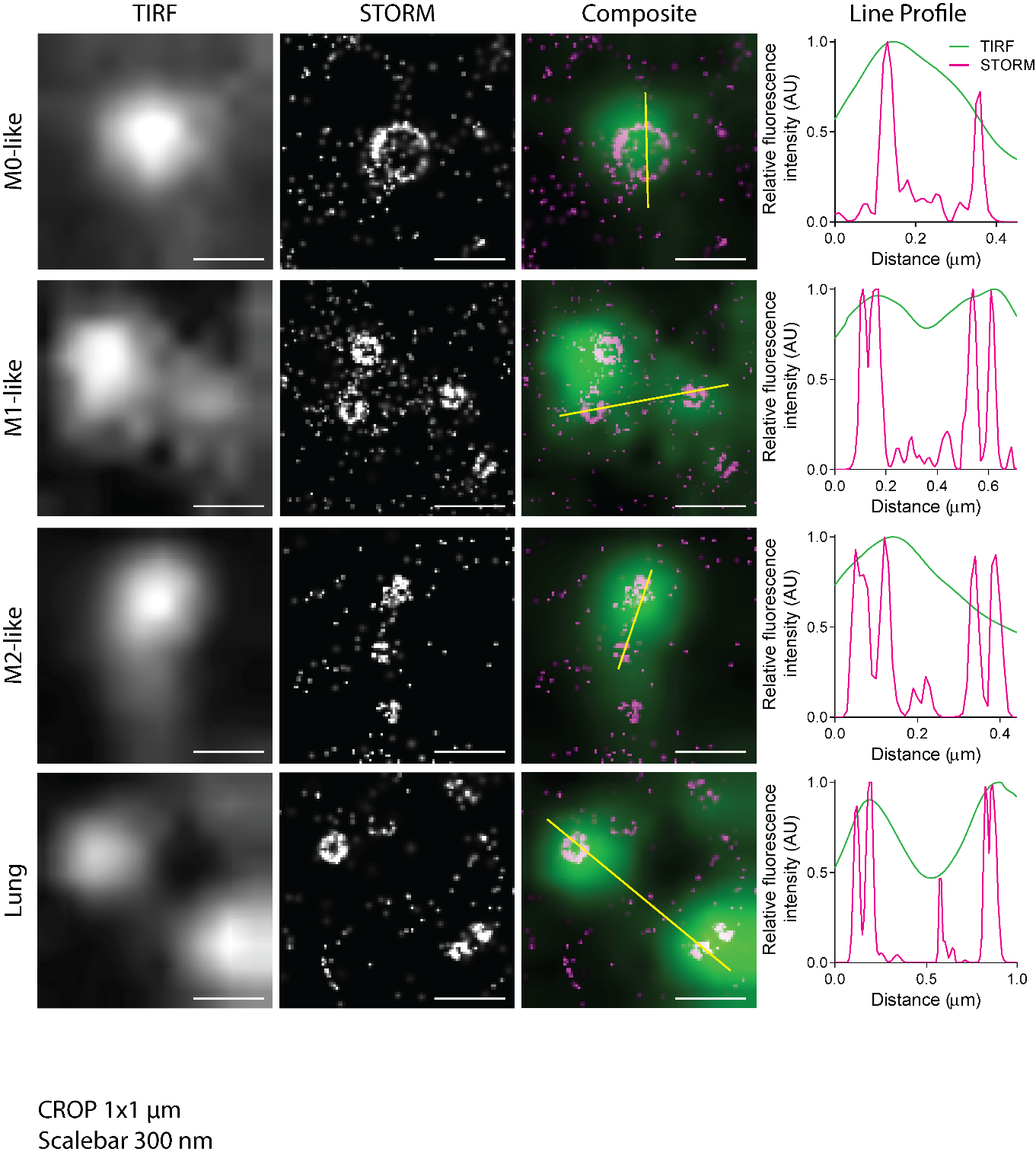


**Supplementary Figure 6: Bright spots of CD81 in TIRF resolve to ring structures using STORM.** Macrophages were incubated on 0.01% PLL + 10 µg/mL IgG from human serum coated glass slides for 15 min, fixed with 4% PFA, blocked with 3% BSA with 1% human serum and stained with CD81-AF647. Samples were then imaged using STORM. Images are representative zoomed sections of bright spots observed in TIRF with corresponding STORM images and associated line profiles (Indicated by yellow lines on composite images). Scale bar: 300 nm.


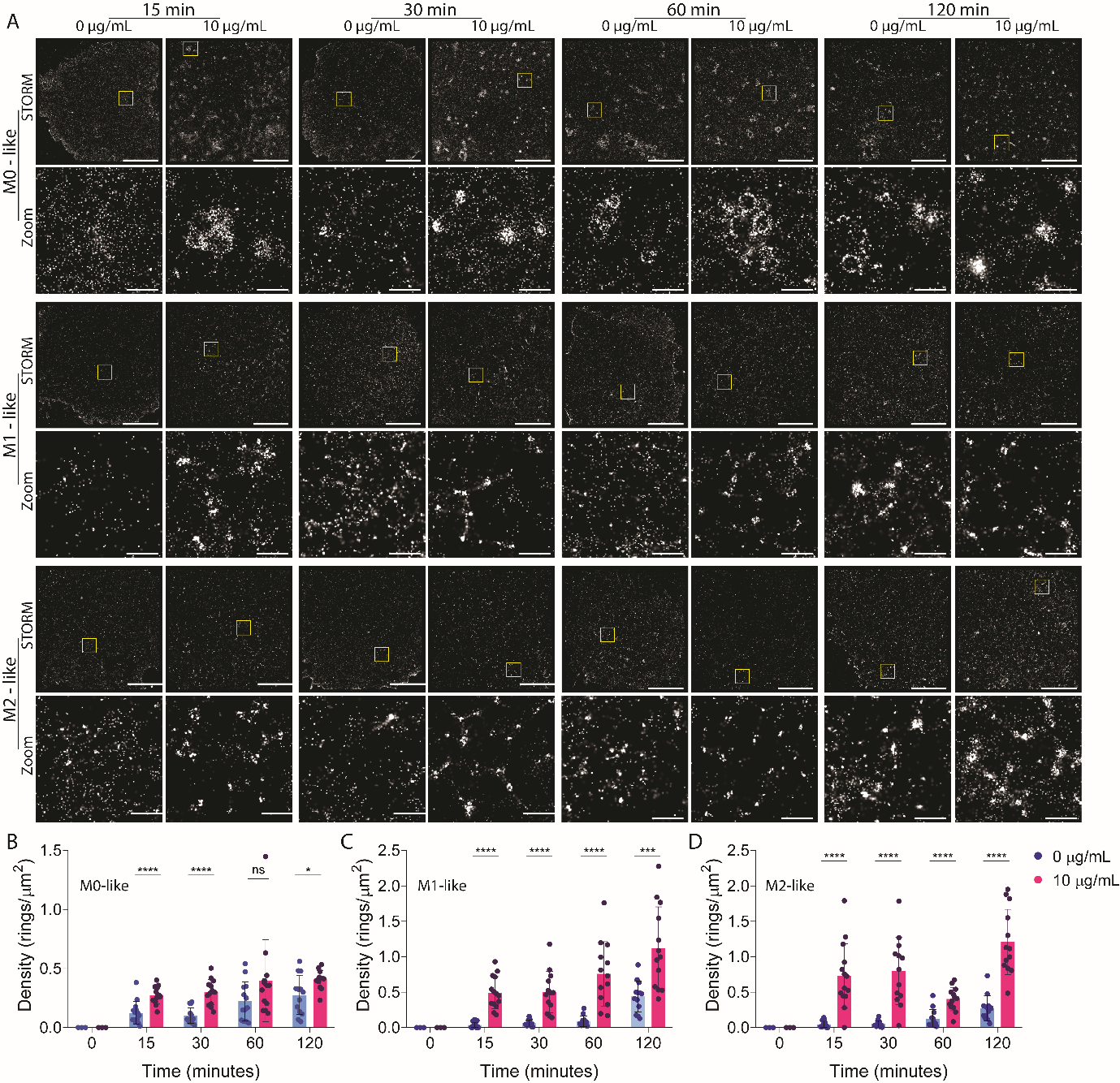


**Supplementary Figure 7: The formation of CD81 nanometer-scale ring structures increases over time.** Macrophages were incubated on glass slides coated with PLL only (0 μg/mL IgG, non-activating condition) or PLL and IgG (10 μg/mL IgG, activating condition) for 15, 30, 60 or 120 min, fixed, blocked and stained with anti-CD81 mAb conjugated with AF647. **A)** Representative STORM images and zoomed in regions (2 x 2 µm) (Scale bar 5 µm, Zoom 0.5 µm, Yellow boxes; STORM Zoom). B) Ring densities of activated and non-activated conditions and all time points. One dot represents one cell. Geometric mean ± SD of n = 12 – 14 cells per condition from 2 individual donors and experiments. ns, not significant; *, p ≤ 0.05; ***, p ≤ 0.001; ****, p ≤ 0.0001; Statistical significance assessed by unpaired t-test.

**
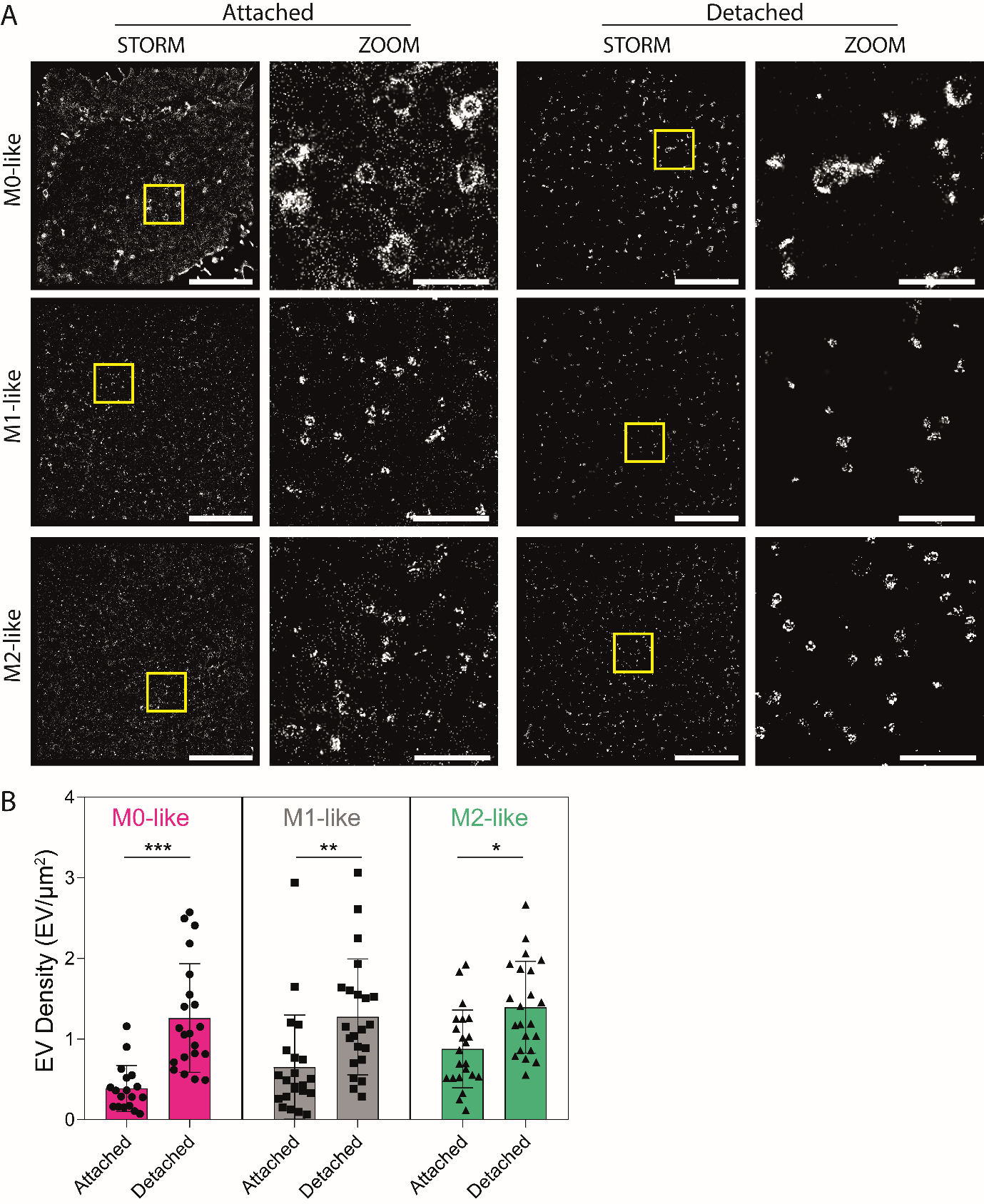
**

**Supplementary Figure 8: Detachment of macrophages leads to higher amounts of EVs being detected**. Macrophages were incubated on IgG-containing lipid bilayers for 20 min and either fixed directly (Attached) or pulse stained with streptavidin-AF488, detached and then fixed (Detached). Macrophages were stained with anti-CD81-AF647 mAb and imaged by STORM. A) Representative STORM images of M0-, M1- and M2-like macrophages with the cell still present (Attached, left) or after the cell was detached (Detached, right). Also shown are zoomed images, represented by the yellow box (Zoom). Scale bar of STORM images 5 µm; Zoom, 1 µm. B) Density of EVs secreted from macrophages. n = 3 individual donors and experiments. *, p ≤ 0.05; **, p ≤ 0.01; ***, p ≤ 0.001; Statistical significance assessed by one-way ANOVA.

**
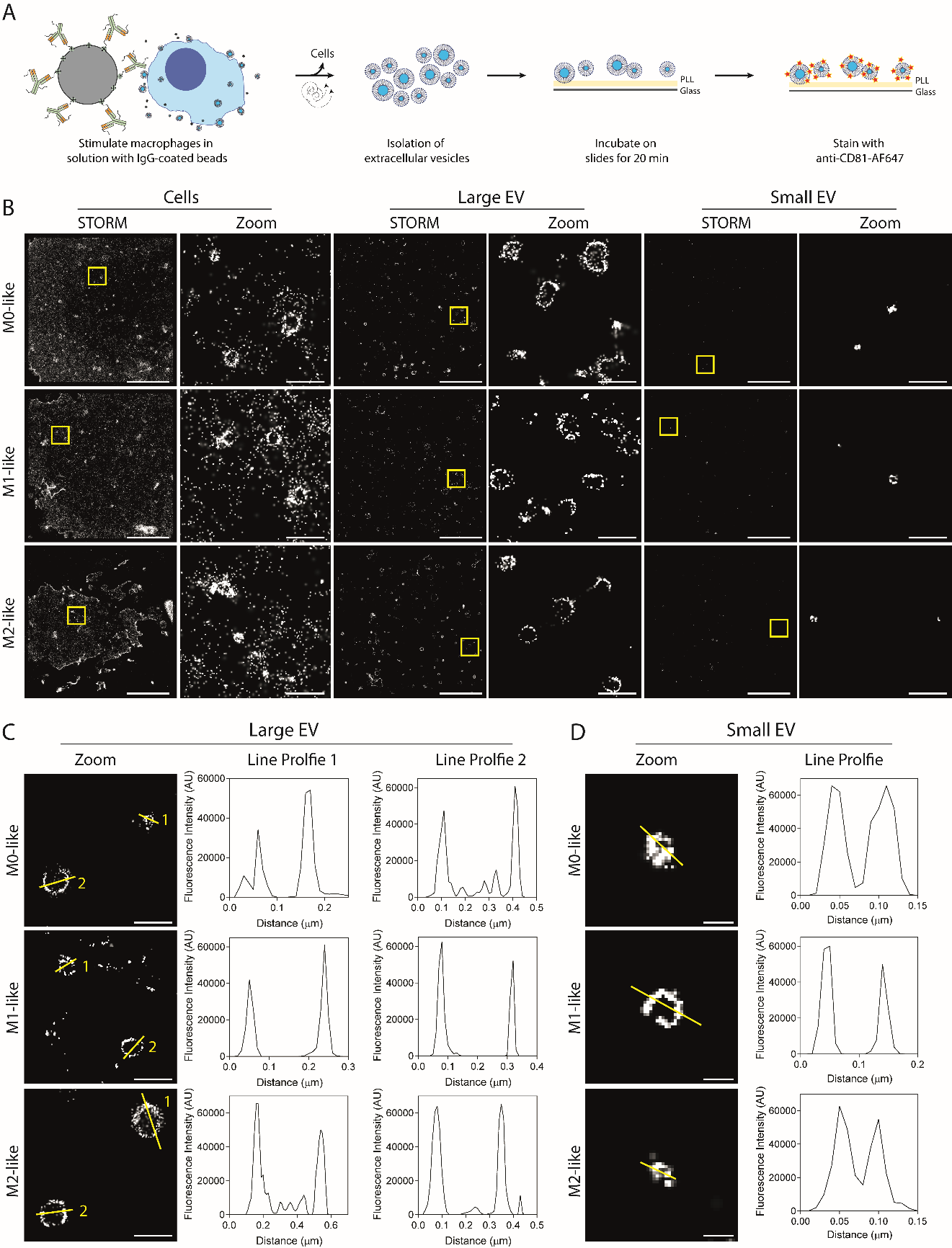
Supplementary Figure 9: Extracellular vesicles were separated into small and large EVs by ultracentrifugation**. A) Macrophages were stimulated in solution by IgG-coated beads to trigger EV secretion. Cells, large EV and small EV were isolated via differential ultracentrifugation, plated onto 0.01% PLL-coated glass slides for 20 min, fixed, blocked and stained with anti-CD81-AF647 mAb. B) Representative STORM and Zoom (2 x 2 µm) images of cells, large and small EVs (Scale bars STORM; 5 µm, Zoom; 0.5 µm, Yellow boxes; STORM Zoom). C) Representative images of large EVs with selected line profiles (Scale bars; 0.5 µm). C) Representative images of the small EV fraction with selected line profiles (Scale bars; 0.1 µm). Images representative of n = 3 individual donors and experiments.


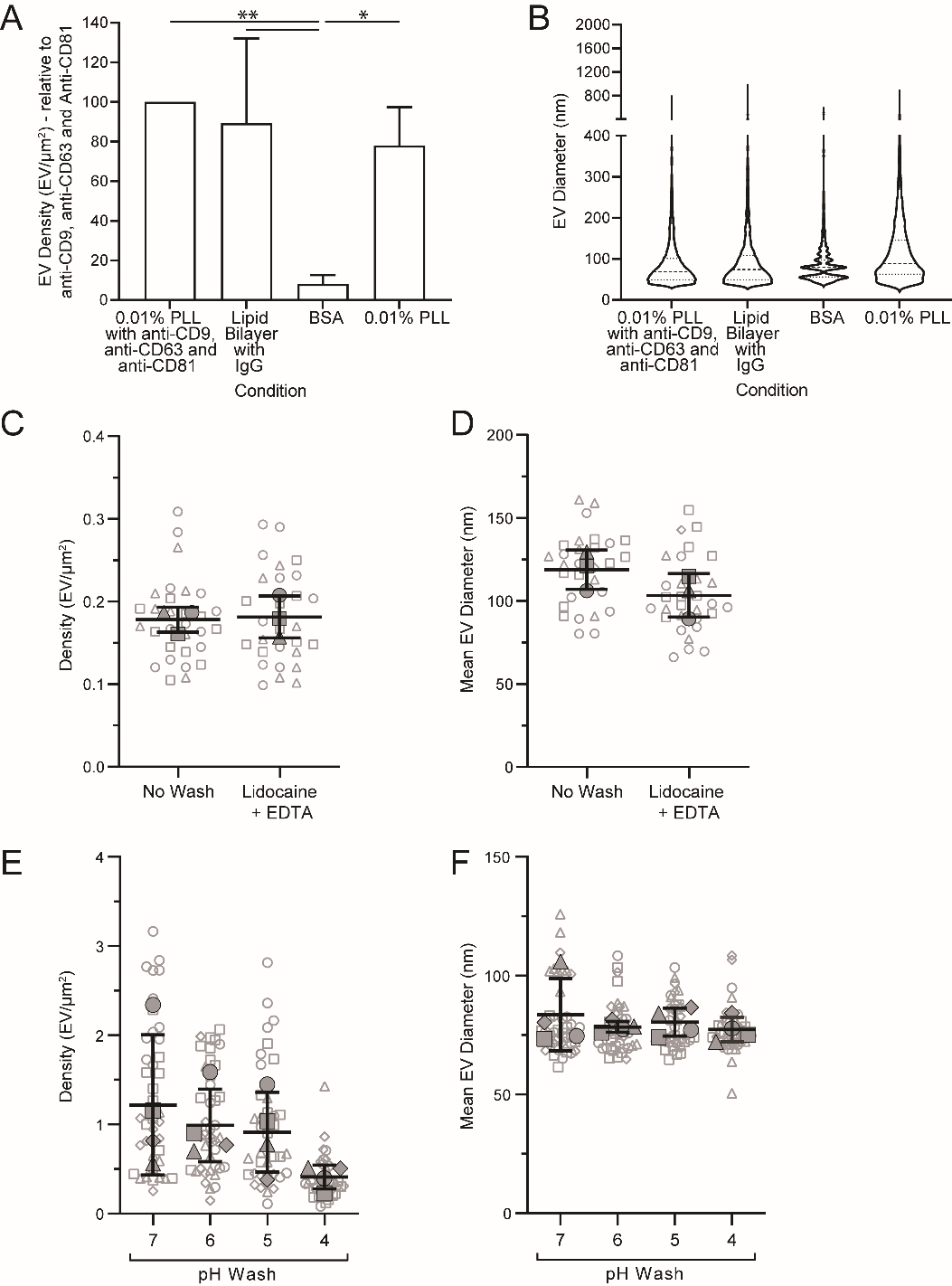


**Supplementary Figure 10: Assessing extracellular vesicle capture**. A-B). M1-like macrophages were stimulated in solution and EV-rich supernatants, generated by differential centrifugation, added to glass chambered coverslips coated with 0.01% PLL with anti-CD9, anti-CD63 and anti-CD81 (5 μg/mL of each), lipid bilayers generated using 10 μg/mL IgG, 3% BSA or 0.01% PLL for 1 h. Samples were washed, blocked and stained with anti-CD9-AF647, anti-CD63-AF647 and anti-CD81-AF647 before imaging by STORM. The EV density relative to anti-tetraspanin capture (A) and EV diameter (B) were measured. n = 4 individual donors and experiments, mean ± SD. C-D) EV-rich supernatants from M1-like macrophages were coated onto lipid bilayers generated with 10 μg/mL IgG, blocked and then stained with anti-CD9-AF647, anti-CD63-AF647 and anti-CD81-AF647. The samples were imaged using STORM, EV density (C) and EV diameter (D) were assessed with and without washing using a lidocaine and EDTA solution. n=3, mean ± SD. M1-like macrophages were activated on lipid bilayers generated using 10 μg/mL IgG for 20 min and detached. *In situ* secretions were incubated at pH 7, 6, 5 or 4 for 1 h before blocking and staining with anti-CD9-AF647, anti-CD63-AF647 and anti-CD81-AF647. Samples were imaged by STORM microscopy, EV density (E) and EV diameter (F) were assessed. n = 4 individual donors and experiments, mean ± SD. *, p ≤ 0.05; **, p ≤ 0.01. Statistical significance assessed by one-way ANOVA (A, B, E, F) or paired t test (C, D).


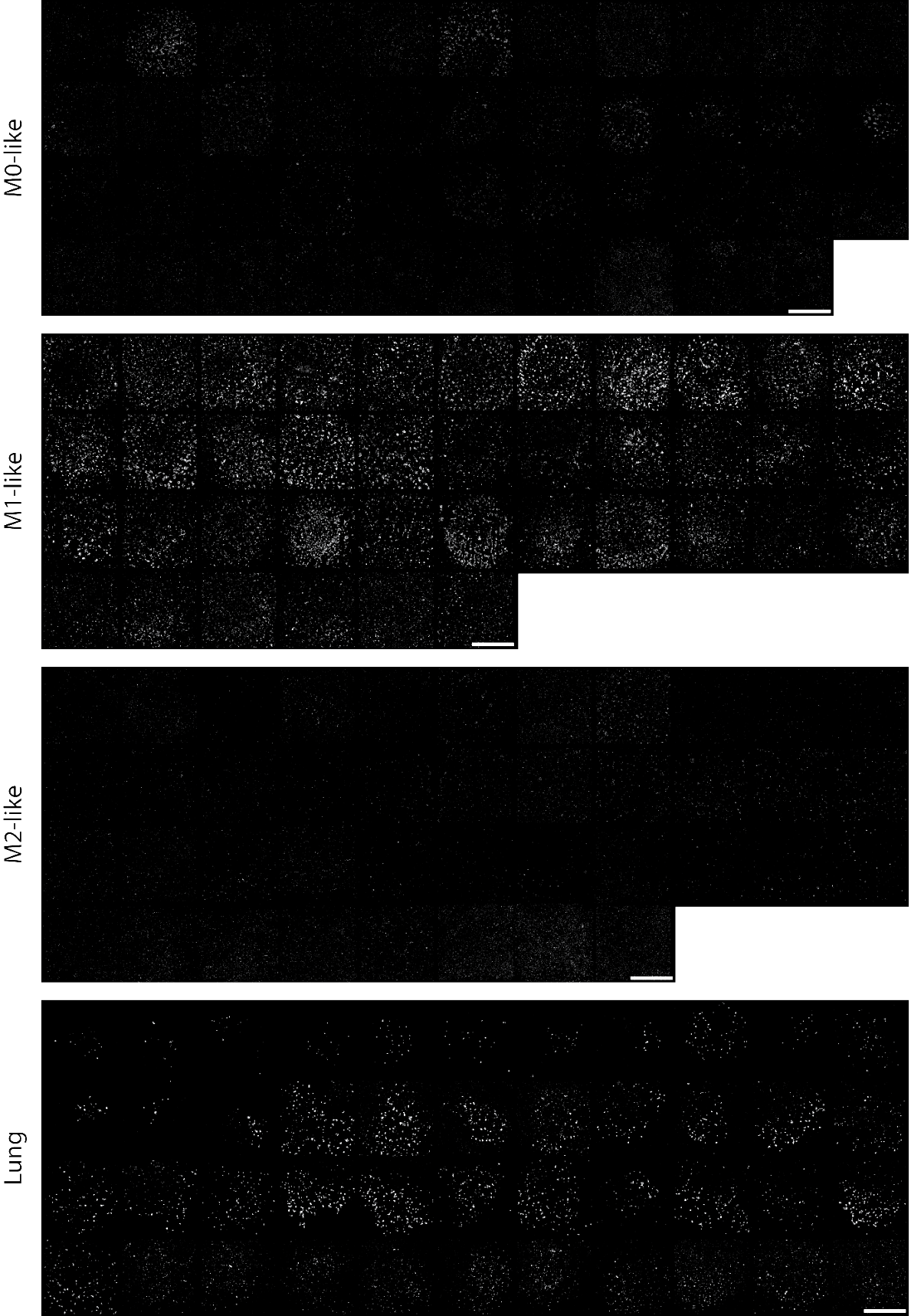


**Supplementary Figure 11: HLA-DR alpha expression montage.** Macrophages were incubated on IgG-containing lipid bilayers for 20 min, detached, fixed, blocked and EV stained with anti-HLA-DR-AF647 mAb before imaging using STORM. Images show montages of all cells from each macrophage group used for Figure 6 and 8 (Scale bar; 10 µm). n = 5 (M0-, M1-, M2-like macrophages) or 4 (lung macrophages) individual donors and experiments.


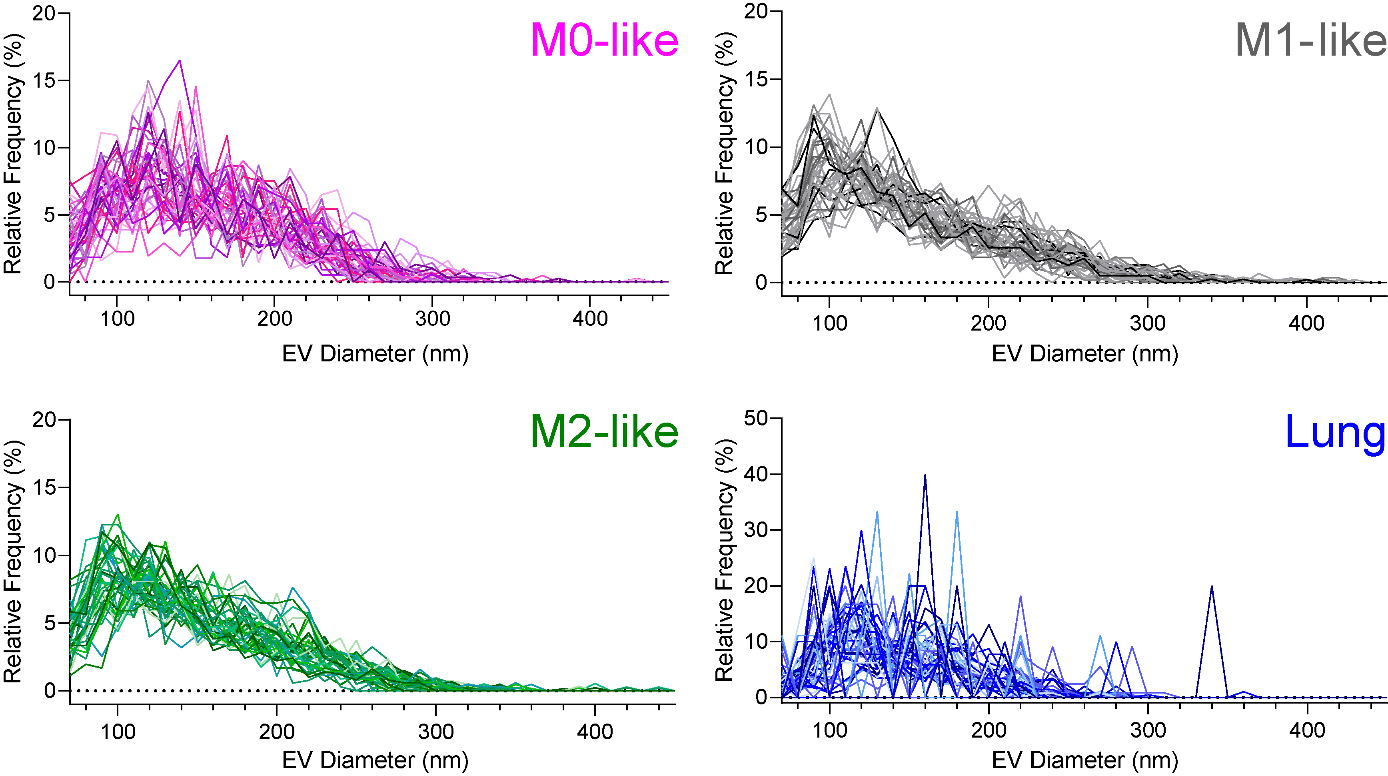


**Supplementary Figure 12: Histograms of EV diameters from individual cells.** Macrophages were incubated on IgG-containing lipid bilayers for 20 min, detached, fixed, blocked and EV stained with anti-CD81-AF488, anti-CD63-AF488 and anti-CD9-AF488 mAbs before imaging using STORM. Images were then analysed to detect EV and their diameters measured and a histogram for each cell plotted with 10 nm binning. n = 39-44 cells from 5 (M0-, M1-, M2-like macrophages) or 4 (lung macrophages) individual donors and experiments

**
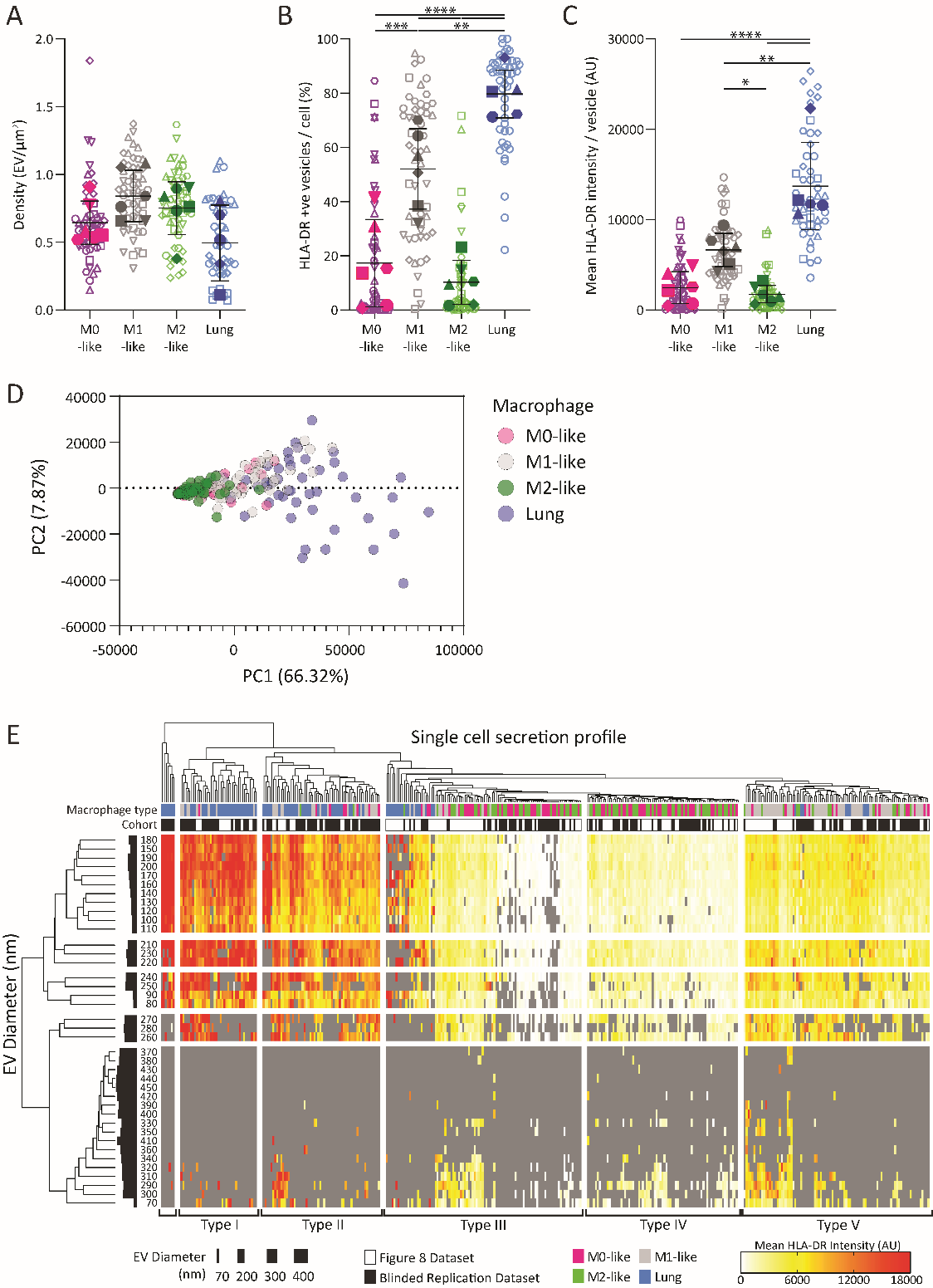
**

**Supplementary Figure 13: Comparing the EV profiles of individual macrophages in a second cohort, analysed in a blinded experiment.** M0-, M1-, M2-like and lung macrophage samples were blinded and then activated on lipid bilayers generated using 10 µg/mL IgG for 20 mins, blocked and stained with anti-CD9-AF488, anti-CD63-AF488, anti-CD81-AF647 and anti-HLA-DR-AF647 before imaging by STORM. A) Density of EV secreted from each cell. B) Percentage of secreted EVs expressing HLA-DR per cell. C) Mean fluorescence intensity of HLA-DR on individual EVs, summarised for each cell. D) Principal components analysis resolving the heterogeneity of individual EVs for each individual cell with macrophage subtype indicated. E) A heatmap combining the blinded and un-blinded datasets of individual cell secretion profiles of HLA-DR on EV of different sizes with unbiased hierarchical clustering of individual cells and EV diameter bins. Representative of 378 cells sourced from 11 (M0-, M1- and M2-like macrophages) or 9 (lung macrophages) individual donors. *, p ≤ 0.05; **, p ≤ 0.01; ***, p ≤ 0.001; ****, p ≤ 0.0001. Statistical significance assessed by one-way ANOVA.

**Supplementary Table 1: Lung tissue donor demographics.** Patient demographics and clinical characteristics broken down by individual figures and summarised for all samples used in the paper. Data is presented as mean ± SD or n (%).

| **Figure(s)** | **Number of patient samples** | **Gender** | | **Age** | **Smoking history** | | |
| --- | --- | --- | --- | --- | --- | --- | --- |
|  |  | **Female** | **Male** |  | **Never** | **Ex** | **Current** |
| Figure 7 | 10 | 5 (50%) | 5 (50%) | 74.2 ± 6.8 | 1 (10%) | 5 (50%) | 4 (40%) |
| Figure 8 and Supplementary Figures 10 & 11 | 4 | 2 (50%) | 2 (50%) | 73.0 ± 6.1 | 1 (25%) | 3 (75%) | 0 (0%) |
| Supplementary Figure 6 | 1 | 0 (0%) | 1 (100%) | 75.0 ± 0.0 | 0 (0%) | 0 (0%) | 1 (100%) |
| Supplementary Figure 13 A-D | 5 | 3 (60%) | 2 (40%) | 73.0 ± 4.2 | 0 (0%) | 4 (80%) | 1 (20%) |
| Supplementary Figure 13 E | 9 | 5 (55.6%) | 4 (44.4%) | 73.6 ± 6.1 | 1 (11.1%) | 7 (77.8%) | 1 (11.1%) |
| Overall | 29 | 15 (51.7%) | 14 (48.3%) | 74.0 ± 5.9 | 3 (10.3%) | 19 (65.5%) | 7  (24.2%) |

**Supplementary Table 2: Data overview.** All measurements from the manuscript are summarized, indicating the figure, macrophage type, experimental condition, parameter that was measured, unit, mean, mode, SD and number of measurements.

| Fig. | Macrophage | | Condition | Measurement | Unit | Mean | Mode | SD | n |
| --- | --- | --- | --- | --- | --- | --- | --- | --- | --- |
| 1 A | M0-like | | 0 µg/mL IgG | TNFα | pg/mL | 26 | - | 27 | 3 |
| 1 A | M0-like | | 1 µg/mL IgG | TNFα | pg/mL | 267 | - | 321 | 3 |
| 1 A | M0-like | | 10 µg/mL IgG | TNFα | pg/mL | 514 | - | 564 | 3 |
| 1 A | M0-like | | 100 µg/mL IgG | TNFα | pg/mL | 997 | - | 1204 | 3 |
| 1 A | M1-like | | 0 µg/mL IgG | TNFα | pg/mL | 125 | - | 38 | 3 |
| 1 A | M1-like | | 1 µg/mL IgG | TNFα | pg/mL | 827 | - | 376 | 3 |
| 1 A | M1-like | | 10 µg/mL IgG | TNFα | pg/mL | 1323 | - | 548 | 3 |
| 1 A | M1-like | | 100 µg/mL IgG | TNFα | pg/mL | 1563 | - | 1039 | 3 |
| 1 A | M2-like | | 0 µg/mL IgG | TNFα | pg/mL | 110 | - | 119 | 3 |
| 1 A | M2-like | | 1 µg/mL IgG | TNFα | pg/mL | 1978 | - | 755 | 3 |
| 1 A | M2-like | | 10 µg/mL IgG | TNFα | pg/mL | 2103 | - | 446 | 3 |
| 1 A | M2-like | | 100 µg/mL IgG | TNFα | pg/mL | 2381 | - | 426 | 3 |
| 1 B | M0-like | | 0 µg/mL IgG | IL-10 | pg/mL | not detected | | | 3 |
| 1 B | M0-like | | 1 µg/mL IgG | IL-10 | pg/mL | not detected | | | 3 |
| 1 B | M0-like | | 10 µg/mL IgG | IL-10 | pg/mL | not detected | | | 3 |
| 1 B | M0-like | | 100 µg/mL IgG | IL-10 | pg/mL | 13 | - | 11 | 3 |
| 1 B | M1-like | | 0 µg/mL IgG | IL-10 | pg/mL | not detected | | | 3 |
| 1 B | M1-like | | 1 µg/mL IgG | IL-10 | pg/mL | 1 | - | 1 | 3 |
| 1 B | M1-like | | 10 µg/mL IgG | IL-10 | pg/mL | 21 | - | 37 | 3 |
| 1 B | M1-like | | 100 µg/mL IgG | IL-10 | pg/mL | 114 | - | 181 | 3 |
| 1 B | M2-like | | 0 µg/mL IgG | IL-10 | pg/mL | 267 | - | 248 | 3 |
| 1 B | M2-like | | 1 µg/mL IgG | IL-10 | pg/mL | 1116 | - | 813 | 3 |
| 1 B | M2-like | | 10 µg/mL IgG | IL-10 | pg/mL | 1270 | - | 381 | 3 |
| 1 B | M2-like | | 100 µg/mL IgG | IL-10 | pg/mL | 1765 | - | 665 | 3 |
| 1 D | M0-like | | 0 µg/mL IgG | Cell area | µm² | 366.9 | - | 359.7 | 675 |
| 1 D | M0-like | | 10 µg/mL IgG | Cell area | µm² | 670.0 | - | 533.1 | 393 |
| 1 D | M1-like | | 0 µg/mL IgG | Cell area | µm² | 362.0 | - | 224.9 | 420 |
| 1 D | M1-like | | 10 µg/mL IgG | Cell area | µm² | 842.7 | - | 549.7 | 390 |
| 1 D | M2-like | | 0 µg/mL IgG | Cell area | µm² | 759.7 | - | 442.1 | 158 |
| 1 D | M2-like | | 10 µg/mL IgG | Cell area | µm² | 1473.0 | - | 858.8 | 160 |
| 1 F | M0-like | | 0 µg/mL IgG | Vesicle density | EV/µm² | 0.11 | - | 0.11 | 20 |
| 1 F | M0-like | | 10 µg/mL IgG | Vesicle density | EV/µm² | 0.29 | - | 0.12 | 24 |
| 1 F | M1-like | | 0 µg/mL IgG | Vesicle density | EV/µm² | 0.09 | - | 0.13 | 34 |
| 1 F | M1-like | | 10 µg/mL IgG | Vesicle density | EV/µm² | 0.74 | - | 0.40 | 33 |
| 1 F | M2-like | | 0 µg/mL IgG | Vesicle density | EV/µm² | 0.07 | - | 0.06 | 26 |
| 1 F | M2-like | | 10 µg/mL IgG | Vesicle density | EV/µm² | 0.47 | - | 0.33 | 27 |
| 1 G | M0-like | | 10 µg/mL IgG | Diameter | nm | - | 159 | 78 | 2142 |
| 1 G | M1-like | | 10 µg/mL IgG | Diameter | nm | - | 75 | 24 | 2139 |
| 1 G | M2-like | | 10 µg/mL IgG | Diameter | nm | - | 66 | 18 | 3162 |
| 1 H | M0-like | | 10 µg/mL IgG | Diameter | nm | 182 | - | 95 | 2142 |
| 1 H | M1-like | | 10 µg/mL IgG | Diameter | nm | 94 | - | 47 | 2139 |
| 1 H | M2-like | | 10 µg/mL IgG | Diameter | nm | 77 | - | 41 | 3162 |
| Fig. | Macrophage | | Condition | Measurement | Unit | Mean | Mode | SD | n |
| 2 C | M0-like | | 10 µg/mL IgG | Vesicle density | EV/µm² | 0.39 | - | 0.29 | 18 |
| 2 C | M1-like | | 10 µg/mL IgG | Vesicle density | EV/µm² | 0.65 | - | 0.65 | 22 |
| 2 C | M2-like | | 10 µg/mL IgG | Vesicle density | EV/µm² | 0.88 | - | 0.48 | 22 |
| 2 D | M0-like | | 10 µg/mL IgG | Diameter | nm | - | 85 | 26 | 1933 |
| 2 D | M1-like | | 10 µg/mL IgG | Diameter | nm | - | 68 | 19 | 4174 |
| 2 D | M2-like | | 10 µg/mL IgG | Diameter | nm | - | 73 | 23 | 4733 |
| 2 E | M0-like | | 10 µg/mL IgG | Diameter | nm | 115 | - | 75 | 1933 |
| 2 E | M1-like | | 10 µg/mL IgG | Diameter | nm | 87 | - | 56 | 4174 |
| 2 E | M2-like | | 10 µg/mL IgG | Diameter | nm | 94 | - | 64 | 4733 |
| 3 C | M0-like | | 10 µg/mL IgG | Vesicle density | EV/µm² | 1.26 | - | 0.67 | 21 |
| 3 C | M1-like | | 10 µg/mL IgG | Vesicle density | EV/µm² | 1.28 | - | 0.72 | 22 |
| 3 C | M2-like | | 10 µg/mL IgG | Vesicle density | EV/µm² | 1.39 | - | 0.57 | 22 |
| 3 D | M0-like | | 10 µg/mL IgG | Diameter | nm | - | 93 | 34 | 5177 |
| 3 D | M1-like | | 10 µg/mL IgG | Diameter | nm | - | 80 | 23 | 9074 |
| 3 D | M2-like | | 10 µg/mL IgG | Diameter | nm | - | 72 | 25 | 10030 |
| 3 E | M0-like | | 10 µg/mL IgG | Diameter | nm | 122 | - | 76 | 5177 |
| 3 E | M1-like | | 10 µg/mL IgG | Diameter | nm | 96 | - | 46 | 9074 |
| 3 E | M2-like | | 10 µg/mL IgG | Diameter | nm | 100 | - | 52 | 10030 |
| 3 F | M0-like | | 10 µg/mL IgG | EV diameter | nm | 116 | - | 64 | 136 |
| 3 F | M0-like | | 10 µg/mL IgG | EV diameter | nm | 124 | - | 97 | 146 |
| 3 F | M0-like | | 10 µg/mL IgG | EV diameter | nm | 135 | - | 90 | 259 |
| 3 F | M0-like | | 10 µg/mL IgG | EV diameter | nm | 116 | - | 64 | 302 |
| 3 F | M0-like | | 10 µg/mL IgG | EV diameter | nm | 164 | - | 121 | 139 |
| 3 F | M0-like | | 10 µg/mL IgG | EV diameter | nm | 149 | - | 94 | 294 |
| 3 F | M0-like | | 10 µg/mL IgG | EV diameter | nm | 137 | - | 83 | 264 |
| 3 F | M0-like | | 10 µg/mL IgG | EV diameter | nm | 98 | - | 48 | 268 |
| 3 F | M0-like | | 10 µg/mL IgG | EV diameter | nm | 101 | - | 46 | 181 |
| 3 F | M0-like | | 10 µg/mL IgG | EV diameter | nm | 101 | - | 53 | 184 |
| 3 F | M0-like | | 10 µg/mL IgG | EV diameter | nm | 99 | - | 51 | 344 |
| 3 F | M0-like | | 10 µg/mL IgG | EV diameter | nm | 105 | - | 54 | 376 |
| 3 F | M0-like | | 10 µg/mL IgG | EV diameter | nm | 131 | - | 67 | 376 |
| 3 F | M0-like | | 10 µg/mL IgG | EV diameter | nm | 106 | - | 57 | 253 |
| 3 F | M0-like | | 10 µg/mL IgG | EV diameter | nm | 98 | - | 37 | 298 |
| 3 F | M0-like | | 10 µg/mL IgG | EV diameter | nm | 149 | - | 103 | 317 |
| 3 F | M0-like | | 10 µg/mL IgG | EV diameter | nm | 119 | - | 73 | 223 |
| 3 F | M0-like | | 10 µg/mL IgG | EV diameter | nm | 112 | - | 76 | 95 |
| 3 F | M0-like | | 10 µg/mL IgG | EV diameter | nm | 122 | - | 84 | 160 |
| 3 F | M0-like | | 10 µg/mL IgG | EV diameter | nm | 147 | - | 93 | 294 |
| 3 F | M0-like | | 10 µg/mL IgG | EV diameter | nm | 128 | - | 66 | 268 |
| 3 F | M1-like | | 10 µg/mL IgG | EV diameter | nm | 87 | - | 32 | 629 |
| 3 F | M1-like | | 10 µg/mL IgG | EV diameter | nm | 76 | - | 33 | 229 |
| 3 F | M1-like | | 10 µg/mL IgG | EV diameter | nm | 85 | - | 32 | 311 |
| 3 F | M1-like | | 10 µg/mL IgG | EV diameter | nm | 69 | - | 20 | 124 |
| 3 F | M1-like | | 10 µg/mL IgG | EV diameter | nm | 74 | - | 30 | 312 |
| 3 F | M1-like | | 10 µg/mL IgG | EV diameter | nm | 68 | - | 26 | 94 |
| Fig. | Macrophage | | Condition | Measurement | Unit | Mean | Mode | SD | n |
| 3 F | M1-like | | 10 µg/mL IgG | EV diameter | nm | 69 | - | 22 | 168 |
| 3 F | M1-like | | 10 µg/mL IgG | EV diameter | nm | 59 | - | 12 | 155 |
| 3 F | M1-like | | 10 µg/mL IgG | EV diameter | nm | 100 | - | 43 | 735 |
| 3 F | M1-like | | 10 µg/mL IgG | EV diameter | nm | 101 | - | 44 | 1000 |
| 3 F | M1-like | | 10 µg/mL IgG | EV diameter | nm | 98 | - | 46 | 242 |
| 3 F | M1-like | | 10 µg/mL IgG | EV diameter | nm | 78 | - | 29 | 384 |
| 3 F | M1-like | | 10 µg/mL IgG | EV diameter | nm | 98 | - | 43 | 290 |
| 3 F | M1-like | | 10 µg/mL IgG | EV diameter | nm | 108 | - | 50 | 851 |
| 3 F | M1-like | | 10 µg/mL IgG | EV diameter | nm | 97 | - | 49 | 524 |
| 3 F | M1-like | | 10 µg/mL IgG | EV diameter | nm | 104 | - | 50 | 536 |
| 3 F | M1-like | | 10 µg/mL IgG | EV diameter | nm | 100 | - | 43 | 497 |
| 3 F | M1-like | | 10 µg/mL IgG | EV diameter | nm | 117 | - | 76 | 375 |
| 3 F | M1-like | | 10 µg/mL IgG | EV diameter | nm | 107 | - | 51 | 468 |
| 3 F | M1-like | | 10 µg/mL IgG | EV diameter | nm | 97 | - | 43 | 492 |
| 3 F | M1-like | | 10 µg/mL IgG | EV diameter | nm | 92 | - | 47 | 364 |
| 3 F | M1-like | | 10 µg/mL IgG | EV diameter | nm | 109 | - | 57 | 294 |
| 3 F | M2-like | | 10 µg/mL IgG | EV diameter | nm | 94 | - | 48 | 340 |
| 3 F | M2-like | | 10 µg/mL IgG | EV diameter | nm | 90 | - | 60 | 233 |
| 3 F | M2-like | | 10 µg/mL IgG | EV diameter | nm | 86 | - | 34 | 339 |
| 3 F | M2-like | | 10 µg/mL IgG | EV diameter | nm | 83 | - | 40 | 382 |
| 3 F | M2-like | | 10 µg/mL IgG | EV diameter | nm | 91 | - | 36 | 760 |
| 3 F | M2-like | | 10 µg/mL IgG | EV diameter | nm | 95 | - | 43 | 385 |
| 3 F | M2-like | | 10 µg/mL IgG | EV diameter | nm | 132 | - | 75 | 459 |
| 3 F | M2-like | | 10 µg/mL IgG | EV diameter | nm | 101 | - | 58 | 490 |
| 3 F | M2-like | | 10 µg/mL IgG | EV diameter | nm | 123 | - | 62 | 475 |
| 3 F | M2-like | | 10 µg/mL IgG | EV diameter | nm | 105 | - | 51 | 181 |
| 3 F | M2-like | | 10 µg/mL IgG | EV diameter | nm | 101 | - | 51 | 280 |
| 3 F | M2-like | | 10 µg/mL IgG | EV diameter | nm | 96 | - | 45 | 257 |
| 3 F | M2-like | | 10 µg/mL IgG | EV diameter | nm | 103 | - | 61 | 246 |
| 3 F | M2-like | | 10 µg/mL IgG | EV diameter | nm | 112 | - | 63 | 389 |
| 3 F | M2-like | | 10 µg/mL IgG | EV diameter | nm | 83 | - | 33 | 280 |
| 3 F | M2-like | | 10 µg/mL IgG | EV diameter | nm | 99 | - | 47 | 604 |
| 3 F | M2-like | | 10 µg/mL IgG | EV diameter | nm | 97 | - | 36 | 611 |
| 3 F | M2-like | | 10 µg/mL IgG | EV diameter | nm | 89 | - | 42 | 505 |
| 3 F | M2-like | | 10 µg/mL IgG | EV diameter | nm | 101 | - | 63 | 630 |
| 3 F | M2-like | | 10 µg/mL IgG | EV diameter | nm | 110 | - | 52 | 733 |
| 3 F | M2-like | | 10 µg/mL IgG | EV diameter | nm | 91 | - | 42 | 646 |
| 3 F | M2-like | | 10 µg/mL IgG | EV diameter | nm | 105 | - | 52 | 672 |
| 4 A | M0-like | | - | Cell Area | µm² | 792.3 | - | 117.7 | 4 |
| 4 A | M0-like | | Cambinol | Cell Area | µm² | 736.2 | - | 182.7 | 4 |
| 4 A | M1-like | | - | Cell Area | µm² | 963.1 | - | 378.3 | 4 |
| 4 A | M1-like | | Cambinol | Cell Area | µm² | 773.5 | - | 269.7 | 4 |
| 4 A | M2-like | | - | Cell Area | µm² | 1167.4 | - | 262.8 | 4 |
| 4 A | M2-like | | Cambinol | Cell Area | µm² | 995.5 | - | 420.4 | 4 |
|  |  | |  |  |  |  |  |  |  |
| Fig. | Macrophage | | Condition | Measurement | Unit | Mean | Mode | SD | n |
| 4 C | M0-like | | - | Vesicle density | EV/µm² | 0.69 | - | 0.46 | 4 |
| 4 C | M0-like | | Cambinol | Vesicle density | EV/µm² | 0.20 | - | 0.08 | 4 |
| 4 C | M1-like | | - | Vesicle density | EV/µm² | 0.76 | - | 0.10 | 4 |
| 4 C | M1-like | | Cambinol | Vesicle density | EV/µm² | 0.16 | - | 0.17 | 4 |
| 4 C | M2-like | | - | Vesicle density | EV/µm² | 0.70 | - | 0.10 | 4 |
| 4 C | M2-like | | Cambinol | Vesicle density | EV/µm² | 0.11 | - | 0.04 | 4 |
| 4 D | M0-like | | - | Vesicle density | Relative change | 1.00 | - | 0.00 | 4 |
| 4 D | M0-like | | Cambinol | Vesicle density | Relative change | 0.36 | - | 0.16 | 4 |
| 4 D | M1-like | | - | Vesicle density | Relative change | 1.00 | - | 0.00 | 4 |
| 4 D | M1-like | | Cambinol | Vesicle density | Relative change | 0.20 | - | 0.20 | 4 |
| 4 D | M2-like | | - | Vesicle density | Relative change | 1.00 | - | 0.00 | 4 |
| 4 D | M2-like | | Cambinol | Vesicle density | Relative change | 0.16 | - | 0.06 | 4 |
| 4 H | M0-like | | 0 µg/mL IgG | Pearsons correlation co-efficient | AU | 0.173 | - | 0.185 | 28 |
| 4 H | M0-like | | 10 µg/mL IgG | Pearsons correlation co-efficient | AU | 0.031 | - | 0.099 | 31 |
| 4 H | M1-like | | 0 µg/mL IgG | Pearsons correlation  co-efficient | AU | 0.134 | - | 0.173 | 30 |
| 4 H | M1-like | | 10 µg/mL IgG | Pearsons correlation  co-efficient | AU | -0.011 | - | 0.087 | 33 |
| 4 H | M2-like | | 0 µg/mL IgG | Pearsons correlation  co-efficient | AU | 0.191 | - | 0.175 | 18 |
| 4 H | M2-like | | 10 µg/mL IgG | Pearsons correlation  co-efficient | AU | 0.010 | - | 0.083 | 31 |
| 6 A | M0-like | | Large EV - HLA-DRα | Relative Abundance | AU | 0.15 | - | 0.07 | 3 |
| 6 A | M0-like | | Large EV - HLA-DRB1*15 | Relative Abundance | AU | 0.12 | - | 0.11 | 3 |
| 6 A | M0-like | | Small EV - HLA-DRα | Relative Abundance | AU | 0.16 | - | 0.06 | 3 |
| 6 A | M0-like | | Small EV - HLA-DRB1*15 | Relative Abundance | AU | 0.13 | - | 0.08 | 3 |
| 6 A | M1-like | | Large EV - HLA-DRα | Relative Abundance | AU | 0.57 | - | 0.11 | 3 |
| 6 A | M1-like | | Large EV - HLA-DRB1*15 | Relative Abundance | AU | 0.72 | - | 0.24 | 3 |
| 6 A | M1-like | | Small EV - HLA-DRα | Relative Abundance | AU | 0.52 | - | 0.07 | 3 |
| Fig. | Macrophage | | Condition | Measurement | Unit | Mean | Mode | SD | n |
| 6 A | M1-like | | Small EV - HLA-DRB1*15 | Relative Abundance | AU | 0.54 | - | 0.13 | 3 |
| 6 A | M2-like | | Large EV - HLA-DRα | Relative Abundance | AU | 0.28 | - | 0.12 | 3 |
| 6 A | M2-like | | Large EV - HLA-DRB1*15 | Relative Abundance | AU | 0.16 | - | 0.15 | 3 |
| 6 A | M2-like | | Small EV - HLA-DRα | Relative Abundance | AU | 0.33 | - | 0.11 | 3 |
| 6 A | M2-like | | Small EV - HLA-DRB1*15 | Relative Abundance | AU | 0.33 | - | 0.10 | 3 |
| 6 B | M0-like | | 10 µg/mL IgG | HLA-DR (relative gMFI) | AU | 64.19 | - | 23.64 | 3 |
| 6 B | M1-like | | 10 µg/mL IgG | HLA-DR (relative gMFI) | AU | 74.99 | - | 22.18 | 3 |
| 6 B | M2-like | | 10 µg/mL IgG | HLA-DR (relative gMFI) | AU | 89.53 | - | 15.67 | 3 |
| 6 C | M0-like | | 10 µg/mL IgG | HLA-DR+ve cells | % | 93.3 | - | 6.1 | 3 |
| 6 C | M1-like | | 10 µg/mL IgG | HLA-DR+ve cells | % | 98.5 | - | 1.1 | 3 |
| 6 C | M2-like | | 10 µg/mL IgG | HLA-DR+ve cells | % | 98.6 | - | 1.6 | 3 |
| 6 E | M0-like | | 10 µg/mL IgG | Vesicle density | EV/µm² | 1.09 | - | 0.12 | 5 |
| 6 E | M1-like | | 10 µg/mL IgG | Vesicle density | EV/µm² | 1.39 | - | 0.24 | 5 |
| 6 E | M2-like | | 10 µg/mL IgG | Vesicle density | EV/µm² | 1.36 | - | 0.24 | 5 |
| 6 F | M0-like | | 10 µg/mL IgG | HLA-DR+ve EV | % | 12.9 | - | 7.9 | 5 |
| 6 F | M1-like | | 10 µg/mL IgG | HLA-DR+ve EV | % | 39.3 | - | 15.1 | 5 |
| 6 F | M2-like | | 10 µg/mL IgG | HLA-DR+ve EV | % | 10.2 | - | 7.5 | 5 |
| 6 G | M0-like | | 10 µg/mL IgG | HLA-DR intensity per EV | AU | 1995 | - | 987 | 5 |
| 6 G | M1-like | | 10 µg/mL IgG | HLA-DR intensity per EV | AU | 4409 | - | 1894 | 5 |
| 6 G | M2-like | | 10 µg/mL IgG | HLA-DR intensity per EV | AU | 1515 | - | 536 | 5 |
| 6 H | M0-Like | | 10 µg/mL IgG | Mean HLA-DR intensity per EV | AU | 411 | - | 1632 | 244 |
| 6 H | M0-Like | | 10 µg/mL IgG | Mean HLA-DR intensity per EV | AU | 10612 | - | 5496 | 321 |
| 6 H | M0-Like | | 10 µg/mL IgG | Mean HLA-DR intensity per EV | AU | 1601 | - | 1919 | 283 |
| 6 H | M0-Like | | 10 µg/mL IgG | Mean HLA-DR intensity per EV | AU | 933 | - | 1219 | 292 |
| 6 H | M0-Like | | 10 µg/mL IgG | Mean HLA-DR intensity per EV | AU | 1593 | - | 1531 | 463 |
| 6 H | M0-Like | | 10 µg/mL IgG | Mean HLA-DR intensity per EV | AU | 7611 | - | 4976 | 343 |
| 6 H | M0-Like | | 10 µg/mL IgG | Mean HLA-DR intensity per EV | AU | 2325 | - | 2049 | 348 |
| 6 H | M0-Like | | 10 µg/mL IgG | Mean HLA-DR intensity per EV | AU | 3104 | - | 2234 | 383 |
| Fig. | Macrophage | | Condition | Measurement | Unit | Mean | Mode | SD | n |
| 6 H | M0-Like | | 10 µg/mL IgG | Mean HLA-DR intensity per EV | AU | 1429 | - | 2544 | 109 |
| 6 H | M0-Like | | 10 µg/mL IgG | Mean HLA-DR intensity per EV | AU | 1109 | - | 1380 | 89 |
| 6 H | M0-Like | | 10 µg/mL IgG | Mean HLA-DR intensity per EV | AU | 908 | - | 1210 | 295 |
| 6 H | M0-Like | | 10 µg/mL IgG | Mean HLA-DR intensity per EV | AU | 2515 | - | 2373 | 306 |
| 6 H | M0-Like | | 10 µg/mL IgG | Mean HLA-DR intensity per EV | AU | 534 | - | 877 | 444 |
| 6 H | M0-Like | | 10 µg/mL IgG | Mean HLA-DR intensity per EV | AU | 2790 | - | 2374 | 292 |
| 6 H | M0-Like | | 10 µg/mL IgG | Mean HLA-DR intensity per EV | AU | 1417 | - | 1590 | 456 |
| 6 H | M0-Like | | 10 µg/mL IgG | Mean HLA-DR intensity per EV | AU | 494 | - | 881 | 393 |
| 6 H | M0-Like | | 10 µg/mL IgG | Mean HLA-DR intensity per EV | AU | 1807 | - | 1731 | 386 |
| 6 H | M0-Like | | 10 µg/mL IgG | Mean HLA-DR intensity per EV | AU | 1656 | - | 1970 | 396 |
| 6 H | M0-Like | | 10 µg/mL IgG | Mean HLA-DR intensity per EV | AU | 4526 | - | 3990 | 240 |
| 6 H | M0-Like | | 10 µg/mL IgG | Mean HLA-DR intensity per EV | AU | 2504 | - | 2727 | 208 |
| 6 H | M0-Like | | 10 µg/mL IgG | Mean HLA-DR intensity per EV | AU | 1915 | - | 2455 | 154 |
| 6 H | M0-Like | | 10 µg/mL IgG | Mean HLA-DR intensity per EV | AU | 4336 | - | 4526 | 206 |
| 6 H | M0-Like | | 10 µg/mL IgG | Mean HLA-DR intensity per EV | AU | 2060 | - | 2391 | 125 |
| 6 H | M0-Like | | 10 µg/mL IgG | Mean HLA-DR intensity per EV | AU | 913 | - | 1402 | 173 |
| 6 H | M0-Like | | 10 µg/mL IgG | Mean HLA-DR intensity per EV | AU | 581 | - | 948 | 371 |
| 6 H | M0-Like | | 10 µg/mL IgG | Mean HLA-DR intensity per EV | AU | 2793 | - | 2636 | 351 |
| 6 H | M0-Like | | 10 µg/mL IgG | Mean HLA-DR intensity per EV | AU | 235 | - | 665 | 187 |
| 6 H | M0-Like | | 10 µg/mL IgG | Mean HLA-DR intensity per EV | AU | 1976 | - | 1991 | 207 |
| 6 H | M0-Like | | 10 µg/mL IgG | Mean HLA-DR intensity per EV | AU | 1483 | - | 2004 | 310 |
| 6 H | M0-Like | | 10 µg/mL IgG | Mean HLA-DR intensity per EV | AU | 808 | - | 1767 | 141 |
| 6 H | M0-Like | | 10 µg/mL IgG | Mean HLA-DR intensity per EV | AU | 1170 | - | 1866 | 266 |
| 6 H | M0-Like | | 10 µg/mL IgG | Mean HLA-DR intensity per EV | AU | 929 | - | 2099 | 522 |
| 6 H | M0-Like | | 10 µg/mL IgG | Mean HLA-DR intensity per EV | AU | 419 | - | 805 | 114 |
| Fig. | Macrophage | | Condition | Measurement | Unit | Mean | Mode | SD | n |
| 6 H | M0-Like | | 10 µg/mL IgG | Mean HLA-DR intensity per EV | AU | 868 | - | 1154 | 183 |
| 6 H | M0-Like | | 10 µg/mL IgG | Mean HLA-DR intensity per EV | AU | 1375 | - | 1440 | 453 |
| 6 H | M0-Like | | 10 µg/mL IgG | Mean HLA-DR intensity per EV | AU | 1835 | - | 2128 | 113 |
| 6 H | M0-Like | | 10 µg/mL IgG | Mean HLA-DR intensity per EV | AU | 2326 | - | 2602 | 296 |
| 6 H | M0-Like | | 10 µg/mL IgG | Mean HLA-DR intensity per EV | AU | 719 | - | 3288 | 55 |
| 6 H | M0-Like | | 10 µg/mL IgG | Mean HLA-DR intensity per EV | AU | 1609 | - | 1887 | 207 |
| 6 H | M0-Like | | 10 µg/mL IgG | Mean HLA-DR intensity per EV | AU | 2089 | - | 2230 | 222 |
| 6 H | M0-Like | | 10 µg/mL IgG | Mean HLA-DR intensity per EV | AU | 1107 | - | 1328 | 435 |
| 6 H | M0-Like | | 10 µg/mL IgG | Mean HLA-DR intensity per EV | AU | 2028 | - | 2283 | 301 |
| 6 H | M0-Like | | 10 µg/mL IgG | Mean HLA-DR intensity per EV | AU | 381 | - | 695 | 168 |
| 6 H | M1-Like | | 10 µg/mL IgG | Mean HLA-DR intensity per EV | AU | 4446 | - | 3748 | 232 |
| 6 H | M1-Like | | 10 µg/mL IgG | Mean HLA-DR intensity per EV | AU | 4808 | - | 3559 | 442 |
| 6 H | M1-Like | | 10 µg/mL IgG | Mean HLA-DR intensity per EV | AU | 5698 | - | 4427 | 456 |
| 6 H | M1-Like | | 10 µg/mL IgG | Mean HLA-DR intensity per EV | AU | 5229 | - | 3810 | 379 |
| 6 H | M1-Like | | 10 µg/mL IgG | Mean HLA-DR intensity per EV | AU | 7674 | - | 4768 | 336 |
| 6 H | M1-Like | | 10 µg/mL IgG | Mean HLA-DR intensity per EV | AU | 5331 | - | 3803 | 321 |
| 6 H | M1-Like | | 10 µg/mL IgG | Mean HLA-DR intensity per EV | AU | 10004 | - | 6105 | 316 |
| 6 H | M1-Like | | 10 µg/mL IgG | Mean HLA-DR intensity per EV | AU | 10264 | - | 5968 | 406 |
| 6 H | M1-Like | | 10 µg/mL IgG | Mean HLA-DR intensity per EV | AU | 8128 | - | 5438 | 381 |
| 6 H | M1-Like | | 10 µg/mL IgG | Mean HLA-DR intensity per EV | AU | 3982 | - | 3837 | 417 |
| 6 H | M1-Like | | 10 µg/mL IgG | Mean HLA-DR intensity per EV | AU | 9670 | - | 6878 | 354 |
| 6 H | M1-Like | | 10 µg/mL IgG | Mean HLA-DR intensity per EV | AU | 5763 | - | 4135 | 446 |
| 6 H | M1-Like | | 10 µg/mL IgG | Mean HLA-DR intensity per EV | AU | 5254 | - | 4421 | 354 |
| 6 H | M1-Like | | 10 µg/mL IgG | Mean HLA-DR intensity per EV | AU | 4797 | - | 3152 | 415 |
| 6 H | M1-Like | | 10 µg/mL IgG | Mean HLA-DR intensity per EV | AU | 7814 | - | 5615 | 407 |
| Fig. | Macrophage | | Condition | Measurement | Unit | Mean | Mode | SD | n |
| 6 H | M1-Like | | 10 µg/mL IgG | Mean HLA-DR intensity per EV | AU | 4319 | - | 3714 | 587 |
| 6 H | M1-Like | | 10 µg/mL IgG | Mean HLA-DR intensity per EV | AU | 2187 | - | 2222 | 401 |
| 6 H | M1-Like | | 10 µg/mL IgG | Mean HLA-DR intensity per EV | AU | 1717 | - | 2516 | 377 |
| 6 H | M1-Like | | 10 µg/mL IgG | Mean HLA-DR intensity per EV | AU | 4743 | - | 4140 | 448 |
| 6 H | M1-Like | | 10 µg/mL IgG | Mean HLA-DR intensity per EV | AU | 2512 | - | 2645 | 479 |
| 6 H | M1-Like | | 10 µg/mL IgG | Mean HLA-DR intensity per EV | AU | 3648 | - | 3549 | 367 |
| 6 H | M1-Like | | 10 µg/mL IgG | Mean HLA-DR intensity per EV | AU | 3847 | - | 4221 | 250 |
| 6 H | M1-Like | | 10 µg/mL IgG | Mean HLA-DR intensity per EV | AU | 3906 | - | 4103 | 434 |
| 6 H | M1-Like | | 10 µg/mL IgG | Mean HLA-DR intensity per EV | AU | 3331 | - | 3609 | 481 |
| 6 H | M1-Like | | 10 µg/mL IgG | Mean HLA-DR intensity per EV | AU | 2444 | - | 2166 | 563 |
| 6 H | M1-Like | | 10 µg/mL IgG | Mean HLA-DR intensity per EV | AU | 5065 | - | 3545 | 620 |
| 6 H | M1-Like | | 10 µg/mL IgG | Mean HLA-DR intensity per EV | AU | 3894 | - | 3670 | 456 |
| 6 H | M1-Like | | 10 µg/mL IgG | Mean HLA-DR intensity per EV | AU | 3752 | - | 3338 | 592 |
| 6 H | M1-Like | | 10 µg/mL IgG | Mean HLA-DR intensity per EV | AU | 2628 | - | 2917 | 521 |
| 6 H | M1-Like | | 10 µg/mL IgG | Mean HLA-DR intensity per EV | AU | 3259 | - | 3468 | 678 |
| 6 H | M1-Like | | 10 µg/mL IgG | Mean HLA-DR intensity per EV | AU | 4113 | - | 4046 | 543 |
| 6 H | M1-Like | | 10 µg/mL IgG | Mean HLA-DR intensity per EV | AU | 1680 | - | 2250 | 282 |
| 6 H | M1-Like | | 10 µg/mL IgG | Mean HLA-DR intensity per EV | AU | 3342 | - | 3630 | 389 |
| 6 H | M1-Like | | 10 µg/mL IgG | Mean HLA-DR intensity per EV | AU | 3073 | - | 3329 | 297 |
| 6 H | M1-Like | | 10 µg/mL IgG | Mean HLA-DR intensity per EV | AU | 3603 | - | 3493 | 487 |
| 6 H | M1-Like | | 10 µg/mL IgG | Mean HLA-DR intensity per EV | AU | 1741 | - | 2075 | 707 |
| 6 H | M1-Like | | 10 µg/mL IgG | Mean HLA-DR intensity per EV | AU | 2485 | - | 3089 | 483 |
| 6 H | M1-Like | | 10 µg/mL IgG | Mean HLA-DR intensity per EV | AU | 2078 | - | 2212 | 750 |
| 6 H | M1-Like | | 10 µg/mL IgG | Mean HLA-DR intensity per EV | AU | 1560 | - | 1920 | 631 |
| 6 H | M2-Like | | 10 µg/mL IgG | Mean HLA-DR intensity per EV | AU | 463 | - | 972 | 209 |
| Fig. | Macrophage | | Condition | Measurement | Unit | Mean | Mode | SD | n |
| 6 H | M2-Like | | 10 µg/mL IgG | Mean HLA-DR intensity per EV | AU | 737 | - | 1157 | 392 |
| 6 H | M2-Like | | 10 µg/mL IgG | Mean HLA-DR intensity per EV | AU | 489 | - | 1166 | 317 |
| 6 H | M2-Like | | 10 µg/mL IgG | Mean HLA-DR intensity per EV | AU | 2092 | - | 1959 | 355 |
| 6 H | M2-Like | | 10 µg/mL IgG | Mean HLA-DR intensity per EV | AU | 463 | - | 933 | 387 |
| 6 H | M2-Like | | 10 µg/mL IgG | Mean HLA-DR intensity per EV | AU | 1584 | - | 2207 | 283 |
| 6 H | M2-Like | | 10 µg/mL IgG | Mean HLA-DR intensity per EV | AU | 2514 | - | 2049 | 335 |
| 6 H | M2-Like | | 10 µg/mL IgG | Mean HLA-DR intensity per EV | AU | 5552 | - | 3824 | 314 |
| 6 H | M2-Like | | 10 µg/mL IgG | Mean HLA-DR intensity per EV | AU | 505 | - | 1034 | 392 |
| 6 H | M2-Like | | 10 µg/mL IgG | Mean HLA-DR intensity per EV | AU | 563 | - | 895 | 436 |
| 6 H | M2-Like | | 10 µg/mL IgG | Mean HLA-DR intensity per EV | AU | 540 | - | 861 | 422 |
| 6 H | M2-Like | | 10 µg/mL IgG | Mean HLA-DR intensity per EV | AU | 702 | - | 1021 | 380 |
| 6 H | M2-Like | | 10 µg/mL IgG | Mean HLA-DR intensity per EV | AU | 997 | - | 1317 | 450 |
| 6 H | M2-Like | | 10 µg/mL IgG | Mean HLA-DR intensity per EV | AU | 946 | - | 1215 | 389 |
| 6 H | M2-Like | | 10 µg/mL IgG | Mean HLA-DR intensity per EV | AU | 1162 | - | 1432 | 382 |
| 6 H | M2-Like | | 10 µg/mL IgG | Mean HLA-DR intensity per EV | AU | 2272 | - | 2369 | 291 |
| 6 H | M2-Like | | 10 µg/mL IgG | Mean HLA-DR intensity per EV | AU | 1760 | - | 1807 | 366 |
| 6 H | M2-Like | | 10 µg/mL IgG | Mean HLA-DR intensity per EV | AU | 2744 | - | 2562 | 354 |
| 6 H | M2-Like | | 10 µg/mL IgG | Mean HLA-DR intensity per EV | AU | 2254 | - | 2276 | 600 |
| 6 H | M2-Like | | 10 µg/mL IgG | Mean HLA-DR intensity per EV | AU | 2934 | - | 2625 | 523 |
| 6 H | M2-Like | | 10 µg/mL IgG | Mean HLA-DR intensity per EV | AU | 3082 | - | 2755 | 434 |
| 6 H | M2-Like | | 10 µg/mL IgG | Mean HLA-DR intensity per EV | AU | 3164 | - | 3329 | 440 |
| 6 H | M2-Like | | 10 µg/mL IgG | Mean HLA-DR intensity per EV | AU | 1659 | - | 2141 | 580 |
| 6 H | M2-Like | | 10 µg/mL IgG | Mean HLA-DR intensity per EV | AU | 772 | - | 1382 | 503 |
| 6 H | M2-Like | | 10 µg/mL IgG | Mean HLA-DR intensity per EV | AU | 2095 | - | 2411 | 402 |
| 6 H | M2-Like | | 10 µg/mL IgG | Mean HLA-DR intensity per EV | AU | 264 | - | 572 | 673 |
| Fig. | Macrophage | | Condition | Measurement | Unit | Mean | Mode | SD | n |
| 6 H | M2-Like | | 10 µg/mL IgG | Mean HLA-DR intensity per EV | AU | 789 | - | 1739 | 428 |
| 6 H | M2-Like | | 10 µg/mL IgG | Mean HLA-DR intensity per EV | AU | 753 | - | 1422 | 398 |
| 6 H | M2-Like | | 10 µg/mL IgG | Mean HLA-DR intensity per EV | AU | 1116 | - | 1835 | 327 |
| 6 H | M2-Like | | 10 µg/mL IgG | Mean HLA-DR intensity per EV | AU | 923 | - | 1760 | 425 |
| 6 H | M2-Like | | 10 µg/mL IgG | Mean HLA-DR intensity per EV | AU | 1269 | - | 3182 | 428 |
| 6 H | M2-Like | | 10 µg/mL IgG | Mean HLA-DR intensity per EV | AU | 1505 | - | 2267 | 359 |
| 6 H | M2-Like | | 10 µg/mL IgG | Mean HLA-DR intensity per EV | AU | 948 | - | 1361 | 495 |
| 6 H | M2-Like | | 10 µg/mL IgG | Mean HLA-DR intensity per EV | AU | 484 | - | 904 | 514 |
| 6 H | M2-Like | | 10 µg/mL IgG | Mean HLA-DR intensity per EV | AU | 641 | - | 1135 | 636 |
| 6 H | M2-Like | | 10 µg/mL IgG | Mean HLA-DR intensity per EV | AU | 1639 | - | 1800 | 417 |
| 6 H | M2-Like | | 10 µg/mL IgG | Mean HLA-DR intensity per EV | AU | 3153 | - | 2886 | 571 |
| 6 H | M2-Like | | 10 µg/mL IgG | Mean HLA-DR intensity per EV | AU | 1918 | - | 1829 | 519 |
| 6 H | M2-Like | | 10 µg/mL IgG | Mean HLA-DR intensity per EV | AU | 1762 | - | 1731 | 508 |
| 6 H | M2-Like | | 10 µg/mL IgG | Mean HLA-DR intensity per EV | AU | 1727 | - | 1845 | 626 |
| 6 H | M2-Like | | 10 µg/mL IgG | Mean HLA-DR intensity per EV | AU | 1150 | - | 1340 | 442 |
| 7 B | Lung | | 0 µg/mL IgG | TNFα | pg/mL | 21 | - | 42 | 4 |
| 7 B | Lung | | 1 µg/mL IgG | TNFα | pg/mL | 164 | - | 242 | 4 |
| 7 B | Lung | | 10 µg/mL IgG | TNFα | pg/mL | 401 | - | 343 | 4 |
| 7 B | Lung | | 100 µg/mL IgG | TNFα | pg/mL | 551 | - | 399 | 4 |
| 7 C | Lung | | 0 µg/mL IgG | IL-10 | pg/mL | not detected | | | 4 |
| 7 C | Lung | | 1 µg/mL IgG | IL-10 | pg/mL | not detected | | | 4 |
| 7 C | Lung | | 10 µg/mL IgG | IL-10 | pg/mL | not detected | | | 4 |
| 7 C | Lung | | 100 µg/mL IgG | IL-10 | pg/mL | not detected | | | 4 |
| 7 D | Lung | | 0 µg/mL IgG | Cell area | µm² | 178.3 | - | 131.4 | 260 |
| 7 D | Lung | | 10 µg/mL IgG | Cell area | µm² | 430.7 | - | 473.2 | 370 |
| 7 F | Lung | | 10 µg/mL IgG | Vesicle density | EV/µm² | 0.76 | - | 0.39 | 31 |
| 7 G | Lung | | 10 µg/mL IgG | Diameter | nm | 101 | - | 57 | 5889 |
| 7 H | Lung | | 10 µg/mL IgG | Diameter | nm | - | 78 | 23 | 5889 |
| 7 I | Lung | | 10 µg/mL IgG | EV Diameter | nm | 94 | - | 42 | 255 |
| 7 I | Lung | | 10 µg/mL IgG | EV Diameter | nm | 73 | - | 28 | 92 |
| 7 I | Lung | | 10 µg/mL IgG | EV Diameter | nm | 82 | - | 34 | 140 |
| 7 I | Lung | | 10 µg/mL IgG | EV Diameter | nm | 73 | - | 37 | 130 |
| 7 I | Lung | | 10 µg/mL IgG | EV Diameter | nm | 92 | - | 55 | 92 |
| Fig. | Macrophage | | Condition | Measurement | Unit | Mean | Mode | SD | n |
| 7 I | Lung | | 10 µg/mL IgG | EV Diameter | nm | 84 | - | 41 | 67 |
| 7 I | Lung | | 10 µg/mL IgG | EV Diameter | nm | 70 | - | 38 | 91 |
| 7 I | Lung | | 10 µg/mL IgG | EV Diameter | nm | 81 | - | 47 | 96 |
| 7 I | Lung | | 10 µg/mL IgG | EV Diameter | nm | 82 | - | 38 | 70 |
| 7 I | Lung | | 10 µg/mL IgG | EV Diameter | nm | 135 | - | 97 | 120 |
| 7 I | Lung | | 10 µg/mL IgG | EV Diameter | nm | 124 | - | 71 | 239 |
| 7 I | Lung | | 10 µg/mL IgG | EV Diameter | nm | 105 | - | 60 | 406 |
| 7 I | Lung | | 10 µg/mL IgG | EV Diameter | nm | 92 | - | 37 | 317 |
| 7 I | Lung | | 10 µg/mL IgG | EV Diameter | nm | 94 | - | 49 | 134 |
| 7 I | Lung | | 10 µg/mL IgG | EV Diameter | nm | 81 | - | 26 | 53 |
| 7 I | Lung | | 10 µg/mL IgG | EV Diameter | nm | 91 | - | 37 | 231 |
| 7 I | Lung | | 10 µg/mL IgG | EV Diameter | nm | 95 | - | 57 | 254 |
| 7 I | Lung | | 10 µg/mL IgG | EV Diameter | nm | 84 | - | 39 | 386 |
| 7 I | Lung | | 10 µg/mL IgG | EV Diameter | nm | 193 | - | 113 | 21 |
| 7 I | Lung | | 10 µg/mL IgG | EV Diameter | nm | 83 | - | 41 | 156 |
| 7 I | Lung | | 10 µg/mL IgG | EV Diameter | nm | 98 | - | 48 | 145 |
| 7 I | Lung | | 10 µg/mL IgG | EV Diameter | nm | 181 | - | 101 | 68 |
| 7 I | Lung | | 10 µg/mL IgG | EV Diameter | nm | 128 | - | 74 | 35 |
| 7 I | Lung | | 10 µg/mL IgG | EV Diameter | nm | 149 | - | 62 | 27 |
| 7 I | Lung | | 10 µg/mL IgG | EV Diameter | nm | 88 | - | 38 | 320 |
| 7 I | Lung | | 10 µg/mL IgG | EV Diameter | nm | 90 | - | 47 | 466 |
| 7 I | Lung | | 10 µg/mL IgG | EV Diameter | nm | 118 | - | 56 | 278 |
| 7 I | Lung | | 10 µg/mL IgG | EV Diameter | nm | 100 | - | 55 | 233 |
| 7 I | Lung | | 10 µg/mL IgG | EV Diameter | nm | 142 | - | 71 | 283 |
| 7 I | Lung | | 10 µg/mL IgG | EV Diameter | nm | 102 | - | 48 | 479 |
| 7 I | Lung | | 10 µg/mL IgG | EV Diameter | nm | 152 | - | 70 | 205 |
| 8 B | Lung | | 10 µg/mL IgG | Vesicle density | EV/µm² | 0.50 | - | 0.30 | 4 |
| 8 C | Lung | | 10 µg/mL IgG | HLA-DR+ve EV | % | 81.4 | - | 13.3 | 4 |
| 8 D | Lung | | 10 µg/mL IgG | HLA-DR intensity per EV | AU | 9869 | - | 3721 | 4 |
| S1 B | | N/A | 10 µg/mL IgG | Relative Fluorescence Intensity | AU | 0.664 | - | 0.077 | 3 |
| S1 B | | N/A | 10 µg/mL IgG | Relative Fluorescence Intensity | AU | 0.708 | - | 0.069 | 3 |
| S1 B | | N/A | 10 µg/mL IgG | Relative Fluorescence Intensity | AU | 0.532 | - | 0.106 | 8 |
| S4 A | | N/A | Cells detached AF647 | Fourier Ring Correlation calculated resolution | nm | 22 | - | 2 | 26 |
| S4 A | | N/A | Cells attached AF647 | Fourier Ring Correlation calculated resolution | nm | 34 | - | 6 | 69 |
| Fig. | | Macrophage | Condition | Measurement | Unit | Mean | Mode | SD | n |
| S4 A | | N/A | Cells detached AF488 | Fourier Ring Correlation calculated resolution | nm | 44 | - | 12 | 27 |
| S4 B | | N/A | Cells detached AF647 | Uncertainty | nm | 6 | - | 1 | 26 |
| S4 B | | N/A | Cells attached AF647 | Uncertainty | nm | 7 | - | 1 | 69 |
| S4 B | | N/A | Cells detached AF488 | Uncertainty | nm | 12 | - | 2 | 27 |
| S5 D | | M0-like | 0 µg/mL IgG | TNFα | pg/mL | 10 | - | 20 | 4 |
| S5 D | | M0-like | 10 µg/mL IgG | TNFα | pg/mL | 15 | - | 29 | 4 |
| S5 D | | M0-like | 100 µg/mL IgG | TNFα | pg/mL | 57 | - | 12 | 4 |
| S5 D | | M1-like | 0 µg/mL IgG | TNFα | pg/mL | 349 | - | 484 | 4 |
| S5 D | | M1-like | 10 µg/mL IgG | TNFα | pg/mL | 415 | - | 407 | 4 |
| S5 D | | M1-like | 100 µg/mL IgG | TNFα | pg/mL | 1048 | - | 648 | 4 |
| S5 D | | M2-like | 0 µg/mL IgG | TNFα | pg/mL | 301 | - | 81 | 4 |
| S5 D | | M2-like | 10 µg/mL IgG | TNFα | pg/mL | 898 | - | 228 | 4 |
| S5 D | | M2-like | 100 µg/mL IgG | TNFα | pg/mL | 1969 | - | 397 | 4 |
| S7 B | | M0-like | 15 min, 0 µg/mL IgG | Vesicle density | EV/µm² | 0.12 | - | 0.10 | 14 |
| S7 B | | M0-like | 15 min, 10 µg/mL IgG | Vesicle density | EV/µm² | 0.27 | - | 0.07 | 14 |
| S7 B | | M0-like | 30 min, 0 µg/mL IgG | Vesicle density | EV/µm² | 0.10 | - | 0.07 | 13 |
| S7 B | | M0-like | 30 min, 10 µg/mL IgG | Vesicle density | EV/µm² | 0.30 | - | 0.11 | 13 |
| S7 B | | M0-like | 60 min, 0 µg/mL IgG | Vesicle density | EV/µm² | 0.22 | - | 0.17 | 13 |
| S7 B | | M0-like | 60 min, 10 µg/mL IgG | Vesicle density | EV/µm² | 0.40 | - | 0.35 | 13 |
| S7 B | | M0-like | 120 min, 0 µg/mL IgG | Vesicle density | EV/µm² | 0.28 | - | 0.16 | 13 |
| S7 B | | M0-like | 120 min, 10 µg/mL IgG | Vesicle density | EV/µm² | 0.41 | - | 0.08 | 13 |
| S7 C | | M1-like | 15 min, 0 µg/mL IgG | Vesicle density | EV/µm² | 0.04 | - | 0.04 | 14 |
| S7 C | | M1-like | 15 min, 10 µg/mL IgG | Vesicle density | EV/µm² | 0.49 | - | 0.23 | 14 |
| S7 C | | M1-like | 30 min, 0 µg/mL IgG | Vesicle density | EV/µm² | 0.06 | - | 0.04 | 12 |
| S7 C | | M1-like | 30 min, 10 µg/mL IgG | Vesicle density | EV/µm² | 0.50 | - | 0.29 | 13 |
| S7 C | | M1-like | 60 min, 0 µg/mL IgG | Vesicle density | EV/µm² | 0.09 | - | 0.07 | 13 |
| S7 C | | M1-like | 60 min, 10 µg/mL IgG | Vesicle density | EV/µm² | 0.76 | - | 0.45 | 13 |
| S7 C | | M1-like | 120 min, 0 µg/mL IgG | Vesicle density | EV/µm² | 0.44 | - | 0.22 | 13 |
| Fig. | | Macrophage | Condition | Measurement | Unit | Mean | Mode | SD | n |
| S7 C | | M1-like | 120 min, 10 µg/mL IgG | Vesicle density | EV/µm² | 1.12 | - | 0.58 | 13 |
| S7 D | | M2-like | 15 min, 0 µg/mL IgG | Vesicle density | EV/µm² | 0.04 | - | 0.05 | 14 |
| S7 D | | M2-like | 15 min, 10 µg/mL IgG | Vesicle density | EV/µm² | 0.73 | - | 0.46 | 14 |
| S7 D | | M2-like | 30 min, 0 µg/mL IgG | Vesicle density | EV/µm² | 0.05 | - | 0.04 | 13 |
| S7 D | | M2-like | 30 min, 10 µg/mL IgG | Vesicle density | EV/µm² | 0.80 | - | 0.47 | 13 |
| S7 D | | M2-like | 60 min, 0 µg/mL IgG | Vesicle density | EV/µm² | 0.13 | - | 0.13 | 13 |
| S7 D | | M2-like | 60 min, 10 µg/mL IgG | Vesicle density | EV/µm² | 0.40 | - | 0.16 | 13 |
| S7 D | | M2-like | 120 min, 0 µg/mL IgG | Vesicle density | EV/µm² | 0.27 | - | 0.18 | 13 |
| S7 D | | M2-like | 120 min, 10 µg/mL IgG | Vesicle density | EV/µm² | 1.21 | - | 0.46 | 13 |
| S8 B | | M0-like | Attached, 10 µg/mL IgG | Vesicle density | EV/µm² | 0.39 | - | 0.29 | 18 |
| S8 B | | M0-like | Detached, 10 µg/mL IgG | Vesicle density | EV/µm² | 1.26 | - | 0.67 | 21 |
| S8 B | | M1-like | Attached, 10 µg/mL IgG | Vesicle density | EV/µm² | 0.65 | - | 0.65 | 22 |
| S8 B | | M1-like | Detached, 10 µg/mL IgG | Vesicle density | EV/µm² | 1.28 | - | 0.72 | 22 |
| S8 B | | M2-like | Attached, 10 µg/mL IgG | Vesicle density | EV/µm² | 0.88 | - | 0.48 | 22 |
| S8 B | | M2-like | Detached, 10 µg/mL IgG | Vesicle density | EV/µm² | 1.39 | - | 0.57 | 22 |
| S10 A | | M1-like | Anti-tetraspanin | Relative EV Density | % | 100.00 | - | 0.00 | 4 |
| S10 A | | M1-like | Lipid Bilayer with IgG | Relative EV Density | % | 89.36 | - | 42.76 | 4 |
| S10 A | | M1-like | 3% BSA | Relative EV Density | % | 8.10 | - | 4.44 | 4 |
| S10 A | | M1-like | 0.01% PLL | Relative EV Density | % | 78.03 | - | 19.41 | 4 |
| S10 B | | M1-like | Anti-tetraspanin | EV Diameter | nm | 89.05 | - | 62.85 | 4 |
| S10 B | | M1-like | Lipid Bilayer with IgG | EV Diameter | nm | 96.69 | - | 80.83 | 4 |
| S10 B | | M1-like | 3% BSA | EV Diameter | nm | 91.70 | - | 64.03 | 4 |
| S10 B | | M1-like | 0.01% PLL | EV Diameter | nm | 114.6 | - | 78.65 | 4 |
| S10 C | | M1-like | No wash | EV Density | EV/µm² | 0.18 | - | 0.02 | 3 |
| S10 C | | M1-like | Lidocaine + EDTA wash | EV Density | EV/µm² | 0.18 | - | 0.03 | 3 |
| Fig. | | Macrophage | Condition | Measurement | Unit | Mean | Mode | SD | n |
| S10 D | | M1-like | No wash | EV Diameter | nm | 118.8 | - | 11.84 | 3 |
| S10 D | | M1-like | Lidocaine + EDTA wash | EV Density | nm | 103.3 | - | 13.04 | 3 |
| S10 E | | M1-like | pH7 | EV Density | EV/µm² | 1.21 | - | 0.79 | 4 |
| S10 E | | M1-like | pH6 | EV Density | EV/µm² | 0.99 | - | 0.41 | 4 |
| S10 E | | M1-like | pH5 | EV Density | EV/µm² | 0.91 | - | 0.45 | 4 |
| S10 E | | M1-like | pH4 | EV Density | EV/µm² | 0.41 | - | 0.13 | 4 |
| S10 F | | M1-like | pH7 | EV Diameter | nm | 83.67 | - | 15.21 | 4 |
| S10 F | | M1-like | pH6 | EV Diameter | nm | 78.51 | - | 2.29 | 4 |
| S10 F | | M1-like | pH5 | EV Diameter | nm | 80.55 | - | 5.88 | 4 |
| S10 F | | M1-like | pH4 | EV Diameter | nm | 77.42 | - | 5.17 | 4 |
| S13 A | | M0-like | 10 µg/mL IgG | Vesicle density | EV/µm² | 0.65 | - | 0.16 | 6 |
| S13 A | | M1-like | 10 µg/mL IgG | Vesicle density | EV/µm² | 0.84 | - | 0.19 | 6 |
| S13 A | | M2-like | 10 µg/mL IgG | Vesicle density | EV/µm² | 0.75 | - | 0.19 | 6 |
| S13 A | | Lung | 10 µg/mL IgG | Vesicle density | EV/µm² | 0.49 | - | 0.28 | 5 |
| S13 B | | M0-like | 10 µg/mL IgG | HLA-DR+ve EV | % | 17.31 | - | 16.10 | 6 |
| S13 B | | M1-like | 10 µg/mL IgG | HLA-DR+ve EV | % | 52.04 | - | 14.80 | 6 |
| S13 B | | M2-like | 10 µg/mL IgG | HLA-DR+ve EV | % | 10.25 | - | 8.09 | 6 |
| S13 B | | Lung | 10 µg/mL IgG | HLA-DR+ve EV | % | 79.70 | - | 8.79 | 5 |
| S13 C | | M0-like | 10 µg/mL IgG | HLA-DR intensity per EV | AU | 2475 | - | 1776 | 6 |
| S13 C | | M1-like | 10 µg/mL IgG | HLA-DR intensity per EV | AU | 6649 | - | 1833 | 6 |
| S13 C | | M2-like | 10 µg/mL IgG | HLA-DR intensity per EV | AU | 1757 | - | 965 | 6 |
| S13 C | | Lung | 10 µg/mL IgG | HLA-DR intensity per EV | AU | 13710 | - | 4828 | 5 |
